# Impact of Bipyridine and Phenanthroline Incorporation into the Macropa Scaffold on Ba(II)/Ra(II) Chelation

**DOI:** 10.1021/acs.inorgchem.6c02747

**Published:** 2026-07-15

**Authors:** Ileana Merdžo, Clarisse Brossard, Nicolas Lepareur, Carlos Platas-Iglesias, Gabriele Balducci, Raphaël Tripier, Enzo Alessio, Federica Battistin

**Affiliations:** † Department of Chemical and Pharmaceutical Sciences, University of Trieste, Via L. Giorgieri 1, Trieste 34127, Italy; ‡ Univ Rennes, Centre Eugène Marquis, Inrae, Inserm, UMR_A 1341, UMR_S 1241, Institut NUMECAN [(Nutrition, Métabolismes et Cancer)], Avenue de la Bataille Flandres, Dunkerque CS 44229, Rennes Cedex 35042, France; § CICA—Centro Interdisciplinar de Química e Bioloxía and Departamento de Química, Universidade da Coruña, A Coruña 15008, Spain; ∥ UMR-CNRS 6521 CEMCA, Univ Brest, 6 Avenue Victor le Gorgeu, Brest 29238, France

## Abstract

Two new decadentate macrocyclic chelators, **bpycropa** and **phencropa**, were synthesized by incorporating 2,2′-bipyridine and 1,10-phenanthroline units, respectively, into the picolinate-functionalized aza-crown ether scaffold of **macropa**, with the aim of enhancing cavity preorganization for heavy alkaline earth metal complexation. Both ligands and their barium complexes, employed as nonradioactive surrogates for radium-223, were characterized by NMR spectroscopy, mass spectrometry, potentiometric titrations, X-ray crystallography, and DFT calculations. Solid-state structures revealed asymmetric coordination geometries markedly different from the more symmetric arrangement in [Ba­(**macropa**)], reflecting conformational constraints imposed by the aromatic fragments. Under physiologically relevant conditions, **phencropa** exhibited thermodynamic stability toward Ba­(II) comparable to **macropa** (pBa = 11.7 vs 11.6), while **bpycropa** showed lower affinity (pBa = 10.0). Kinetic studies demonstrated enhanced inertness for both systems relative to **macropa**, with dissociation half-lives of 71.3 min for [Ba­(**bpycropa**)] and 160.5 min for [Ba­(**phencropa**)] compared to 21 min for [Ba­(**macropa**)] (20 °C, pH 7.4, 10 mM, *I* = 0.15 M NaCl). Despite these promising results, radiolabeling studies with radium-223 revealed that neither chelator achieved the serum stability of [^223^Ra]­Ra-**macropa**, highlighting the delicate interplay between preorganization, donor atom composition, and kinetic inertness required for effective radium coordination and offering design principles for next-generation chelators.

## Graphical abstract

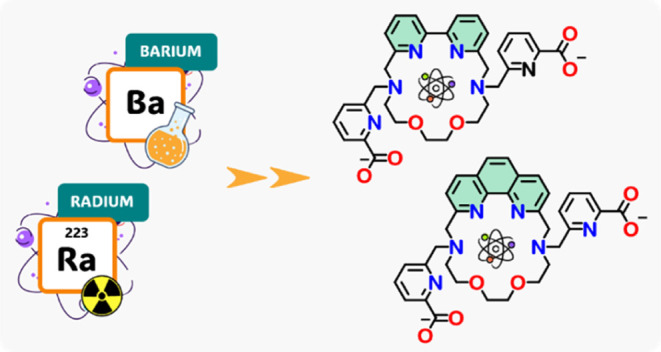

## Introduction

Targeted alpha therapy (TAT) is gaining increasing attention in radiopharmaceutical research as an effective strategy for cancer treatment. The emitted alpha particles have a limited range in biological tissues of approximately 50–100 μm, corresponding to only a few cell diameters. Thus, they deposit large amounts of energy (2–10 MeV) over very short distances and exhibit high linear energy transfer values (LET, 80–100 keV μm^–1^), inducing clustered and largely irreparable DNA double-strand breaks.^1–3^ This spatial constraint makes α-emitting radionuclides particularly well suited for treating small tumors and micrometastatic diseases, where their localized impact translates into reduced off-target toxicity.^1,2,4–6^

Among the α emitters whose physical characteristics are compatible with clinical applications (e.g., actinium-225, bismuth-212/213, terbium-149, thorium-227, and astatine-211), radium-223 (t_1_/_2_ = 11.43 d) occupies a unique position as it is the only α-emitting radionuclide approved by both the U.S. Food and Drug Administration and the European Medicines Agency.^2,6–8^ It is administered as [^223^Ra]­RaCl_2_ (Xofigo) for the palliative treatment of bone metastases associated with castration-resistant prostate cancer.^5,9^ The clinical efficacy of the drug stems from the calcium-mimetic behavior of radium, which enables selective accumulation in osteoblastic lesions through incorporation into the bone hydroxyapatite matrix.^3^

While the natural bone-seeking property of Ra­(II) underpins Xofigo’s success, it also limits broader therapeutic applications. Extending radium-based radiopharmaceuticals to nonosseous tumor targets requires tight sequestration by chelators with appropriate targeting vectors. Besides high selectivity for the target tissue, such systems must combine high thermodynamic stability with sufficient kinetic inertness to preserve radiocomplex integrity under physiological conditions which is a demanding criterion that has proven difficult to satisfy.

Few chelators have been systematically evaluated for heavy alkaline earth metal ions complexation, and available systems generally perform poorly. Conventional chelators such as EDTA and DOTA bind [^223^Ra]­Ra­(II) inefficiently. The coordination number of EDTA is too low for the large ionic radius of Ra­(II), whereas the coordination environment of DOTA does not provide a sufficiently good stereoelectronic match, likely due also to its relative rigidity.^10–14^ Conversely, the decadentate 6,6’-((1,4,10,13-tetraoxa-7,16-diazacyclooctadecane-7,16-diyl)­bis­(methylene))­dipicolinic acid ligand (**macropa**), a derivative of the 18-membered diaza crown ether Kryptofix-22 functionalized with two picolinic acid arms (Figure 1), currently represents the state of the art for ^223^Ra coordination, enabling rapid and efficient radiolabeling and the formation of relatively stable complexes.^10^ However, the bioconjugation of **macropa** to a peptide targeting vector through one of its picolinate rings resulted in decreased in vivo stability of the radium complex, possibly due to interactions between the bifunctional chelator and the linker with the targeting vector fragment.^10^

**Figure 1:**
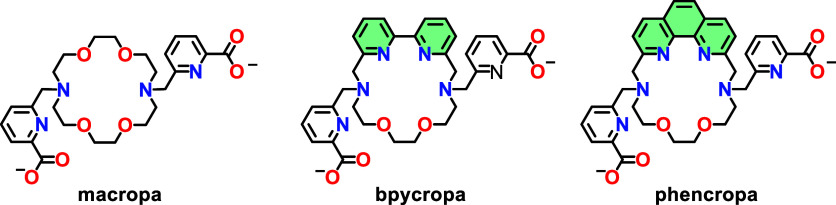
Structures of **macropa** (left) and the chelators developed in this work for the complexation of Ba­(II) and [^223^Ra]­Ra­(II) (**bpycropa** and **phencropa**). The molecules are represented in the fully deprotonated form present in water at pH > 8.

A key structural feature of the Kryptofix-22 platform is the presence of two sp^3^ nitrogen atoms, which serve as versatile functionalization sites for attaching pendant coordinating groups. The nature of the pendant arms has been systematically explored, with malonate, deprotonated 1-hydroxy-2-pyridinone, catecholate, and 2-pyridylphosphonate groups investigated as alternatives to the original picolinate.^15^ The macrocyclic backbone has also been modified, through the introduction of additional nitrogen donors (triaza- and hexaaza-18-crown-6), the use of cyclam-based scaffolds, and the replacement of the macrocyclic core with acyclic 2,6-pyridinedimethanamine-derived frameworks.^16^

However, the original **macropa** continues to remain unchallenged. The insertion of an additional O-ether atom in the macrocycle to make the 21-membered diaza-crown ether chelator named **macropa-XL**, did not afford improved features.^17^

The intensified research activity in this area,^18^ combined with the limitations of current systems, underscores the persistent need for alternative chelator designs that can provide new insights into the coordination chemistry of this demanding metal ion.

In this work, with the aim of expanding the molecular toolbox available for Ra­(II) coordination, we developed two new decadentate **macropa**-like chelators containing either 2,2′-bipyridine or 1,10-phenanthroline in place of an O–CH_2_–CH_2_–O fragment of the macrocycle (**bpycropa** and **phencropa** respectively, Figure 1). The design rationale consists in investigating how aromatic rigidification within the macrocyclic framework influences cavity preorganization, thermodynamic stability, kinetic inertness, and radiolabeling efficiency toward heavy alkaline earth cations, with the aim of understanding whether such structural modifications translate into improved coordination properties. Furthermore, the substitution of two ether oxygens with two sp^2^ nitrogen atoms is expected to improve the directionality of the donor orbitals, which may influence coordination geometry and complex stability through increased donor rigidity and preorganization. In addition, the bipyridine or phenanthroline scaffold might allow positioning the linker for bioconjugation further away from the coordination site, reducing deleterious interactions with the metal ion that could cause its decomplexation.

The newly designed chelators were synthesized and fully characterized. Their coordination properties were investigated using nonradioactive Ba­(II) as a chemical surrogate for Ra­(II), enabling an in-depth thermodynamic, kinetic and structural analysis through a combination of experimental and computational approaches. Finally, radiolabeling experiments with [^223^Ra]­Ra­(II) were performed to evaluate the practical suitability of these systems for targeted alpha therapy applications.

## Results and Discussion

### Synthesis and Characterization of the Ligands

The macrocyclic scaffold **2bpy** was synthesized by following a slightly modified literature procedure: the starting material 2,2′-bipyridine-6,6′-dicarbaldehyde (**1bpy**) was condensed with 1,8-diamino-3,6-dioxaoctane to form a cyclic diimine, followed by NaBH_4_ reduction, to afford the corresponding cyclic diamine (**2bpy**, Scheme 1).^19^ This latter macrocycle was then *N*-alkylated with 6-(chloromethyl)­pyridin-2-carboxylate methyl ester in a microwave reactor to obtain the dimethyl ester intermediate **3bpy**. After chromatographic purification on an alumina column, acidic hydrolysis of the ester groups yielded the final ligand **bpycropa** as its hydrochloride salt. The **phencropa** ligand was similarly obtained as its hydrochloride salt, using 1,10-phenanthroline-2,9-dicarbaldehyde as starting material and following an analogous synthetic procedure (Scheme 1).^20^

**Scheme 1:**
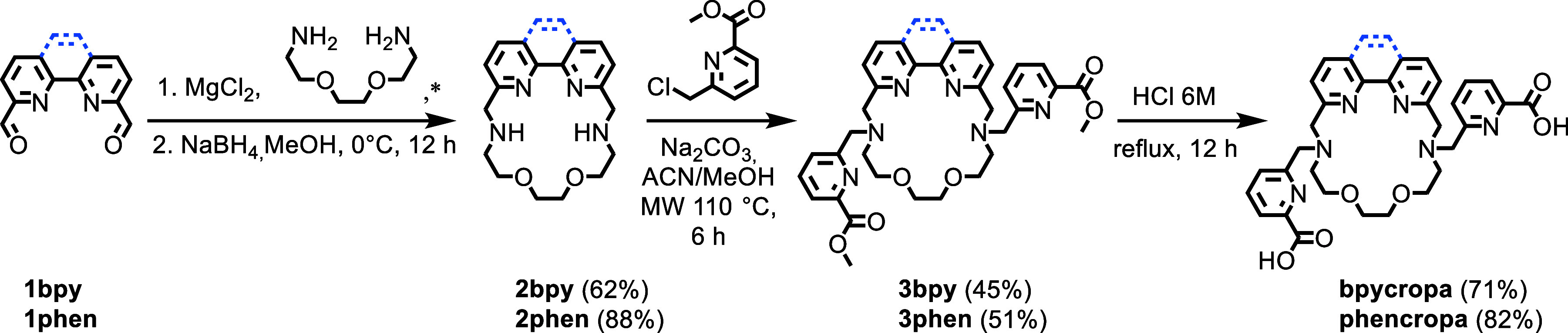
Synthetic Steps for Obtaining the **bpycropa** and **phencropa** Chelators; *1 h for **1bpy**, 6 h for **1phen**

Reaction intermediates and the final compounds were characterized by NMR spectroscopy (mono- and bidimensional) and mass spectrometry (see Supporting Information, Sections 1.1–1.5, 6 and 7). The ^1^H NMR spectrum of each ligand (Figure 2) is consistent with its symmetric structure, showing only half the number of proton resonances expected for the full molecule, i.e. the pairwise chemically equivalent protons are also magnetically equivalent. The resonances were assigned through 2D NMR spectra as well as comparison with the spectra of the precursors and of **macropa**, as detailed in the Supporting Information (Sections 1.6, 1.7).

**Figure 2:**
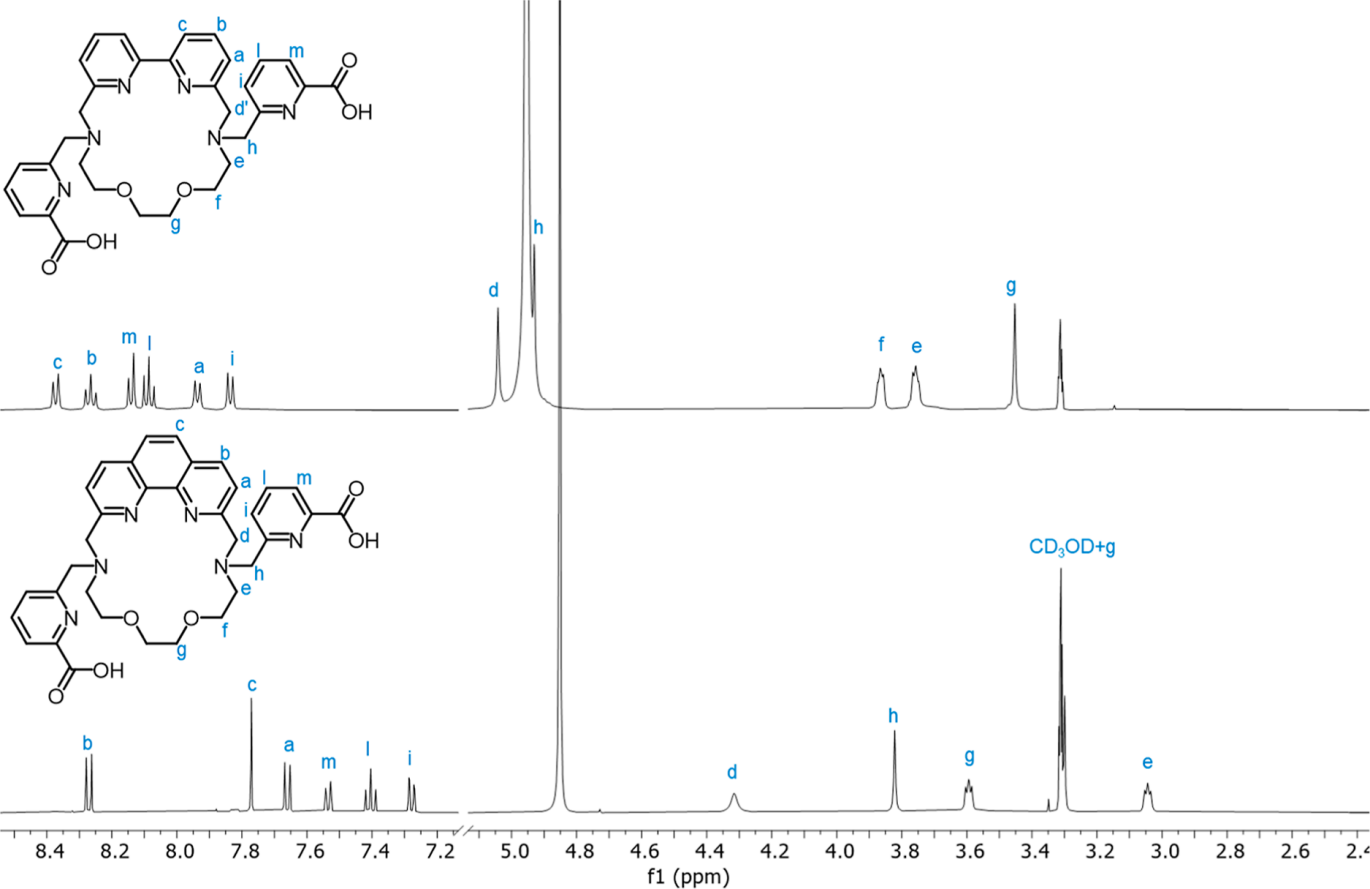
^1^H NMR spectra (500 MHz, 298 K) of **bpycropa** (top) and **phencropa** (bottom) in CD_3_OD with labeling scheme.

### Protonation Constants of **bpycropa** and **phencropa**

Prior to examining the Ra­(II)/Ba­(II) coordination behavior of the **bpycropa** and **phencropa** chelators, their acid–base behavior in aqueous solution was investigated by pH potentiometry and ^1^H NMR titrations and compared to that of **macropa** (Table 1). This preliminary analysis is required because protonation equilibria directly affect metal binding, as H^+^ ions compete with metal cations for donor atoms exhibiting both Brønsted and Lewis basicity.

**Table 1:** Comparison of p*K*_a_ Values for the **macropa**, **bpycropa** and **phencropa** Ligands Obtained by NMR Spectroscopy and by Potentiometry^a^

	**macropa**		**bpycropa**		**phencropa**	
	potentiometric	NMR	potentiometric	NMR	potentiometric	NMR
p*K*_a1_	7.92(3)	7.73(1)^b^	7.86(3)	7.72(1)	7.87(5)	8.11(1)
p*K*_a2_	6.59(3)	6.80(1)^b^	6.31(3)	6.09(2)	6.14(5)	6.42(1)
p*K*_a3_	3.29(5)	3.13(1)^b^	3.69(3)	3.91(1)	3.58(5)	3.88(5)
p*K*_a4_	2.05(1)	2.40(1)^b^	2.51(3)	2.9(1)	2.46(5)	2.9(1)
p*K*_a5_	1.65(1)	1.69(1)^c^	1.74(3)	1.80(2)	–	–

a [NaNO_3_] = 0.15 M, 25 °C.

b p*K*_a_ determined at *T* = 25 °C and *I* = 0.1 M NaCl. Data taken from ref 24.

c p*K*a determined at *T* = 25 °C and *I* = 0.1 M KCl. Data taken from ref 22.

Compared to **macropa**, which has six potential (de)­protonation sites (two sp^3^ N atoms in the macrocycle, two sp^2^ N atoms, and two carboxylate groups on the picolinate arms), **bpycropa** and **phencropa** possess one additional protonation site: the two sp^2^ N atoms of the bipyridine or phenanthroline unit (p*K*_a_ = 4.44 and 4.92 for the free ligands, respectively).^21^

Previous studies of **macropa** identified four distinct protonation constants in the pH range 2–8 (Table 1).^22–24^ The two highest values (6.80 and 7.73) were assigned to the macrocyclic tertiary nitrogens, whereas the two lower p*K*_a_ values (2.40 and 3.13) were ascribed to the carboxylic groups on the picolinate rings. An additional constant at very acidic pH (p*K*_a_ = 1.69) was observed by some Authors (including in this work) and attributed to the protonation of a picolinate nitrogen atom.^22,25^

For **bpycropa** and **phencropa**, ^1^H NMR (Figures S27–S29 for **bpycropa**; S31–S33 for **phencropa**) and potentiometric titrations afforded p*K*_a_ values in good agreement with one another (Table 1). In the NMR spectra, all resonances progressively shifted to lower frequencies upon increasing pH, consistent with ligand deprotonation and the associated increase in electron density. Between pH 6.9 and 8.5, pronounced signal broadening occurs in the aliphatic region (Figure S34), attributed to protonation–deprotonation equilibria and/or conformational movements of the flexible macrocyclic backbone occurring on an intermediate NMR time scale.

As for **macropa**, potentiometric and NMR titration data for **bpycropa** were best described by a five-protonation-constant model (four- and six-constant models were also evaluated for comparison) with good agreement observed between the two methods: the two highest p*K*_a_ values (>6.2) obtained by potentiometric titration, were assigned to stepwise protonation of the macrocyclic amine nitrogen atoms. The three lowest protonation constants (∼3.80 and 2.9–1.8) were attributed to the picolinate carboxylate groups and to an aromatic nitrogen donor, either belonging to the picolinate pendant arm or to the bpy/phen unit. For **phencropa**, although a five-protonation-constant model was similarly considered, both potentiometric and NMR titrations yielded only four determinable constants, likely because the lowest protonation constant fell below the detection limits of the methods employed. The p*K*_a_ determination from NMR data relied on the signal of the aromatic H_b_ proton (Figure S30 and S34, dark blue trace) and four well-resolved aliphatic resonances that remain nonoverlapping across the entire pH range.

Based on the determined p*K*_a_ values, the speciation diagrams for **bpycropa** and **phencropa** as a function of pH were calculated (Figures S32 and S35 respectively).

### Ba­(II) Complexes

The coordination behavior of **bpycropa** and **phencropa** in aqueous solution was investigated using Ba­(II) as a chemical surrogate for Ra­(II). In fact, since all radium isotopes are radioactive, a detailed characterization of Ra­(II) coordination by conventional experimental techniques is inherently challenging. In this context, the use of cold Ba­(II) provides a practical and well-established approach to probe the coordination chemistry of heavy alkaline earth metals under nonradioactive conditions, enabling the collection of thermodynamic and structural information that would be difficult to obtain directly with Ra­(II).^18^

The [Ba­(**bpycropa**)] (**4**) and [Ba­(**phencropa**)] complexes (**5**) were obtained as white-yellowish solids (yields of 95% and 97%, respectively) by reacting, at room temperature, a slight excess of BaCl_2_ (1.1 equiv) with a concentrated aqueous solution of **bpycropa/phencropa**, previously adjusted to pH 7, with complex formation occurring immediately upon mixing The complexes were characterized by single crystal X-ray diffraction, NMR spectroscopy and mass spectrometry (vide infra). Single crystals were obtained by slow evaporation of a methanol solution at room temperature.

X-ray diffraction studies of **4** and **5** (Figure 3, with coordination bond lengths), show that both complexes are neutral, with no additional counterions, consistent with the deprotonation of both carboxylic groups of the picolinate rings. In the solid state, both complexes adopt an asymmetric geometry in which the Ba­(II) cation is deca-coordinate, i.e. all donor atoms of the ligand are coordinated to barium. The asymmetric unit contains two crystallographically independent molecules (Ba1 and Ba2) belonging to the Δ­(λλδ)/Λ­(δδλ) enantiomeric pair (Figures S45 and S46). The crystallographic inversion center generates the other enantiomer, so that the crystal lattice contains both enantiomers in equal amounts (racemate).

**Figure 3:**
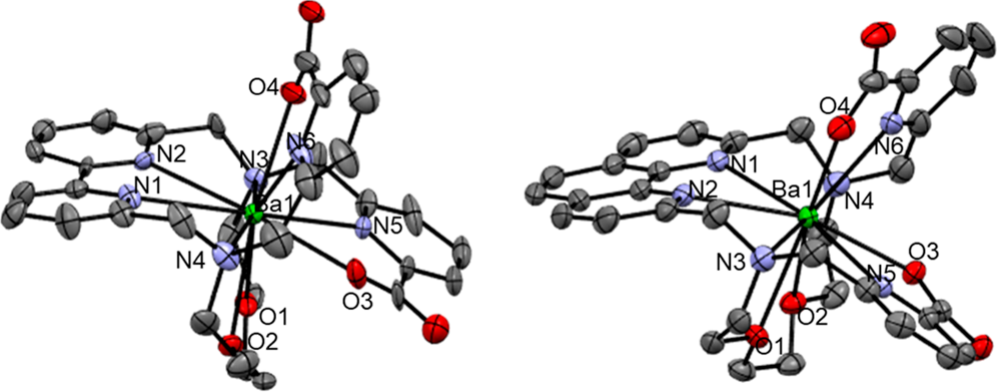
X-ray molecular structure (30% probability ellipsoids) of [Ba­(**bpycropa**)] (**4**, left), and [Ba­(**phencropa**)] (**5**, right), showing one of the two crystallographically independent molecules in the asymmetric unit. Hydrogen atoms and disordered methanol and water molecules have been omitted for clarity. The complete asymmetric units are shown in Figures S45 and S46. Coordination distances (Å): [Ba­(**bpycropa**)]: Ba1–N1 = 3.024(9), Ba1–N2 = 2.962(7), Ba1–N3 = 2.991(7), Ba1–N4 = 3.017(9), Ba1–N5 = 2.912(8), Ba1–N6 = 2.823(8), Ba1–O1 = 2.861(6), Ba1–O2 = 2.969(7), Ba1–O3 = 2.733(8), Ba1–O4 = 2.788(7); [Ba­(**phencropa**)]: Ba1–N1 = 2.925(4), Ba1–N2 = 2.908(4), Ba1–N3 = 2.999(4), Ba1–N4 = 3.042(5), Ba1–N5 = 2.937(4), Ba1–N6 = 2.880(4), Ba1–O1 = 2.894(3), Ba1–O2 = 2.937(3), Ba1–O3 = 2.740(3), Ba1–O4 = 2.746(4).

In each complex, the macrocycle exhibits a highly distorted geometry, with the bent ether portion nearly perpendicular to the bpy/phen plane. Within the barium coordination sphere, two approximately perpendicular mean planes can be identified, each defined by six donor atoms and intersecting at the tertiary amine nitrogens N3 and N4 (Figure 4). Besides these two atoms, one plane contains the bpy/phen nitrogens (N1 and N2) and N5 and O3 of one picolinate, whereas the other plane contains O4 and N6 of the second picolinate moiety and the two ether oxygens O1 and O2 (Figure 3). The angle between the average plane containing N1–N5 and O3 and the bpy/phen moiety was found to be around ∼19° in **4** and slightly larger (∼30°) in **5**. In general, the Ba–heteroatom bond lengths observed in the present complexes are in good agreement with those reported in the literature for related barium compounds.^10,23^ However, the two picolinate units are remarkably different from one another in terms of coordination environment, one (N5–O3) being *trans* to the bpy/phen moiety, and the other (N6–O4) *trans* to the ether oxygen atoms; indeed, in both complexes, the Ba–N5 bond length is significantly longer than Ba–N6, consistent with the stronger *trans*-influence of bpy/phen compared to ether oxygen. The solid-state geometry of both complexes is fully consistent with the NMR spectra in solution, which points to the absence of symmetry elements (vide infra).

**Figure 4:**
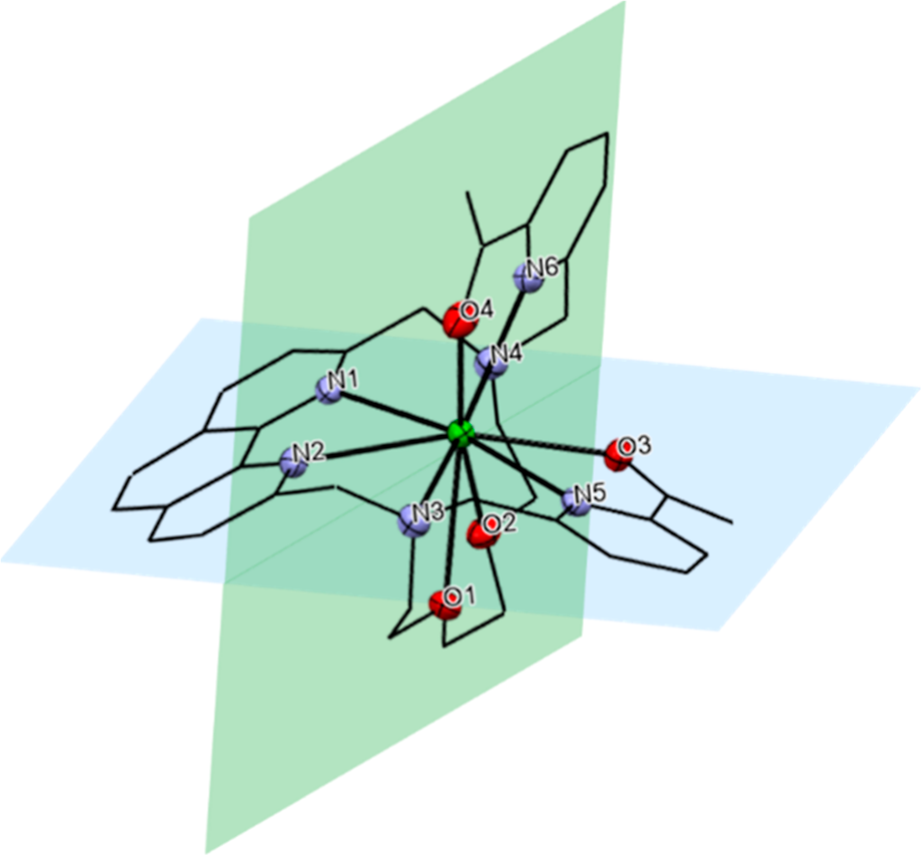
Schematic representation of the two approximately perpendicular mean planes (blue and green) identified within the coordination sphere of [Ba­(**phencropa**)]. The blue plane contains the 1,10-phenanthroline nitrogens (N1, N2), the two amine nitrogens (N3, N4), and the first picolinate group (N5, O3). The green plane contains the second picolinate group (N6, O4) and the ether oxygens (O1, O2). The two planes intersect at N3 and N4.

On the other hand, the solid-state geometries of **4** and **5** differ greatly from that of [Ba­(**macropa**)], in which the ligand coordination to the metal center exhibits a higher degree of symmetry. The X-ray structures of **macropa** with Ba­(II), Ra­(II), La­(III), and Lu­(III) ions consistently show that the six donor atoms of the macrocyclic framework, i.e. the two amine nitrogens (N1 and N2) and the four ether oxygens (O1–O4), define a mean plane around the cation, while the two picolinate rings chelate the metal from above in an antisymmetric (head-to-tail) arrangement.^10,23,26,27^ The presence of a *C*_2_ symmetry axis between the two picolinate arms is consistent with the solution NMR spectrum of [Ba­(**macropa**)], in which they give a single set of three resonances (each integrating for 2H) in the aromatic region.^15,23^

The ^1^H NMR spectra of **4** and **5** (Figure 5) immediately reveal a substantial increase in the number of resonances compared to the free ligands, indicating that upon metal coordination all protons become magnetically nonequivalent and resonate at distinct frequencies due to the loss of symmetry, which is also in agreement with the X-ray crystal structure of each complex. Given the close similarity of the NMR spectra and the identical solution behavior of the two Ba­(II) complexes, only the spectrum of [Ba­(**bpycropa**)] is discussed in detail here, while the full spectroscopic data for both complexes are provided in the Supporting Information (Sections 1.7 and 1.8).

**Figure 5:**
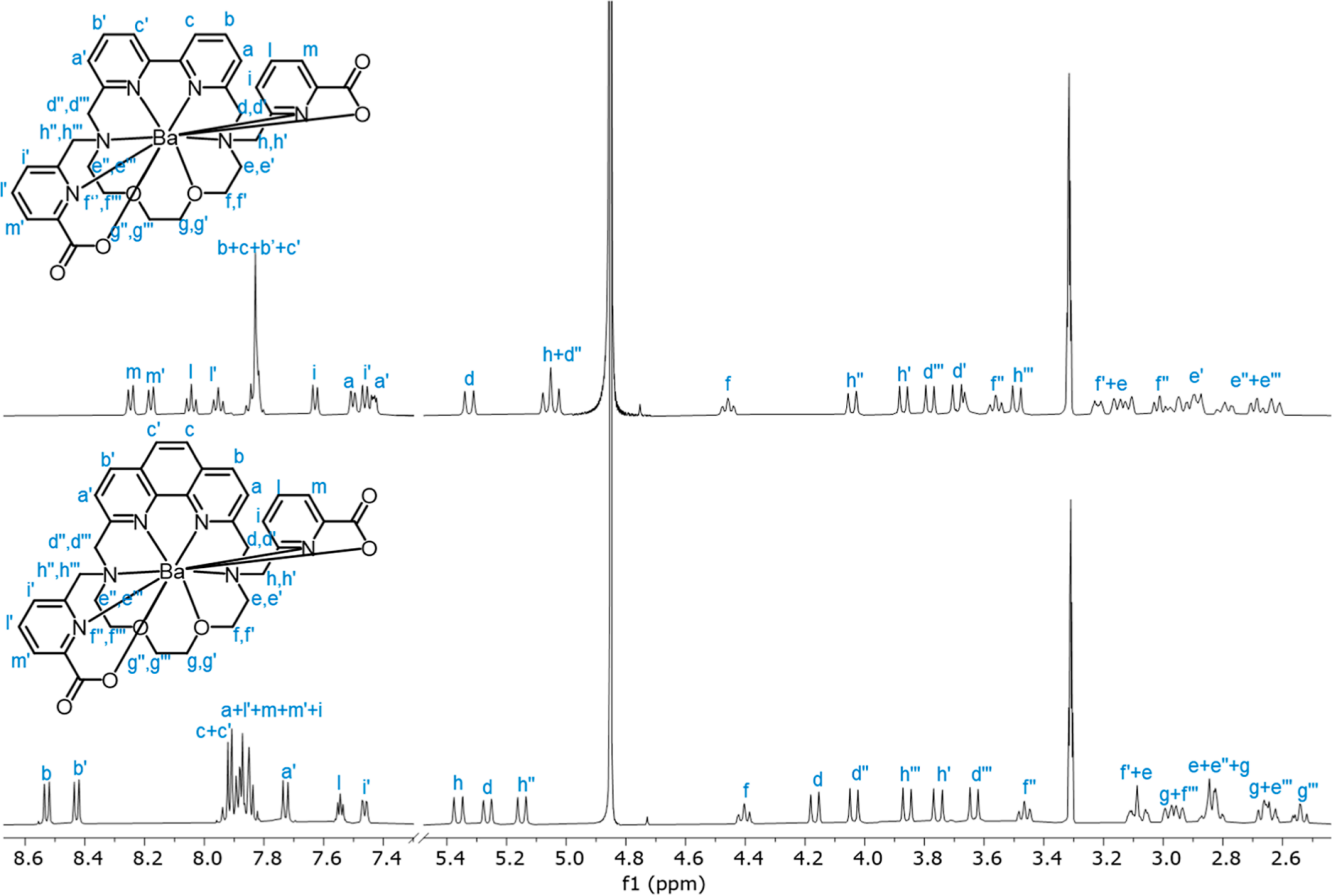
^1^H NMR spectra of [Ba­(**bpycropa**)] (**4**, top) and [Ba­(**phencropa**)] (**5** bottom) in CD_3_OD with labeling scheme. The 2D drawings are not representative of the true geometry of the complexes, which can be seen in Figure 3.

The ^1^H NMR spectrum of **4** shows 13 well-resolved signals—five in the aromatic region and eight in the aliphatic region—each integrating for 1H, whereas the resonances of the remaining 19 protons appear as partially overlapping multiplets. In the aromatic region of the spectrum, the two doublets and two triplets at highest frequencies belong to the H_*m*‑*m*′_ and H_*l*‑*l*′_ picolinate protons, that are more deshielded compared to H_*i*‑*i*′_ due to resonance effects. The signals of the remaining picolinate protons (H_*i*‑*i*′_) appear as doublets at slightly lower frequencies and were assigned through an ^1^H–^1^H COSY spectrum (Figure S21a). The resonances of the bipyridyl protons H_*b*‑*b*′_ and H_*c*‑*c*′_ overlap in a multiplet that shows correlation with the doublets of the H_*a*‑*a*′_ protons. In the aliphatic region of the ^1^H – ^1^H COSY spectrum (Figure S21b), several cross peaks between doublets, corresponding to diastereotopic CH_2_ protons, are observed. The resonances of the most deshielded protons H_*d*‑*d*‴_ and H_*h*‑*h*‴_, due to their proximity to the sp^3^ N atoms and the aromatic fragments, appear at higher frequencies. Similar to what was observed in the free ligand, these protons presented long-range couplings with nearby aromatic partners: for example, the resonance of H_*d*_ correlates with the H_*b*_/H_*c*_ multiplet and the H_*a*_ doublet (Figure S21c, resonances with orange dots), while the resonance of H_*h*″_ shows correlations with those of the picolinate protons H_*m*′_ and H_*i*′_ (Figure S21c, resonances with magenta dots). All resonances relative to the remaining protons (H_*e*‑*e*‴_, H_*f*‑*f*‴_ and H_*g*‑*g*‴_) fall in partially overlapping multiplets at lower frequencies and were further analyzed via ^1^H – ^13^C HSQC and ^1^H – ^13^C HMBC experiments for completeness (Figures S22 and S23). Lastly, an ^1^H – ^1^H ROESY experiment showed that the resonances of chemically equivalent protons are pairwise connected by clear exchange cross peaks, indicating the occurrence of a dynamic process that exchanges them, which is slow on the NMR time scale (Figure 6). For [Ba­(**macropa**)] also a similar conformational equilibrium was detected by ^1^H NMR spectroscopy in solution. However, in that case the higher symmetry of the complex did not allow to establish unambiguously the nature of the process.

**Figure 6:**
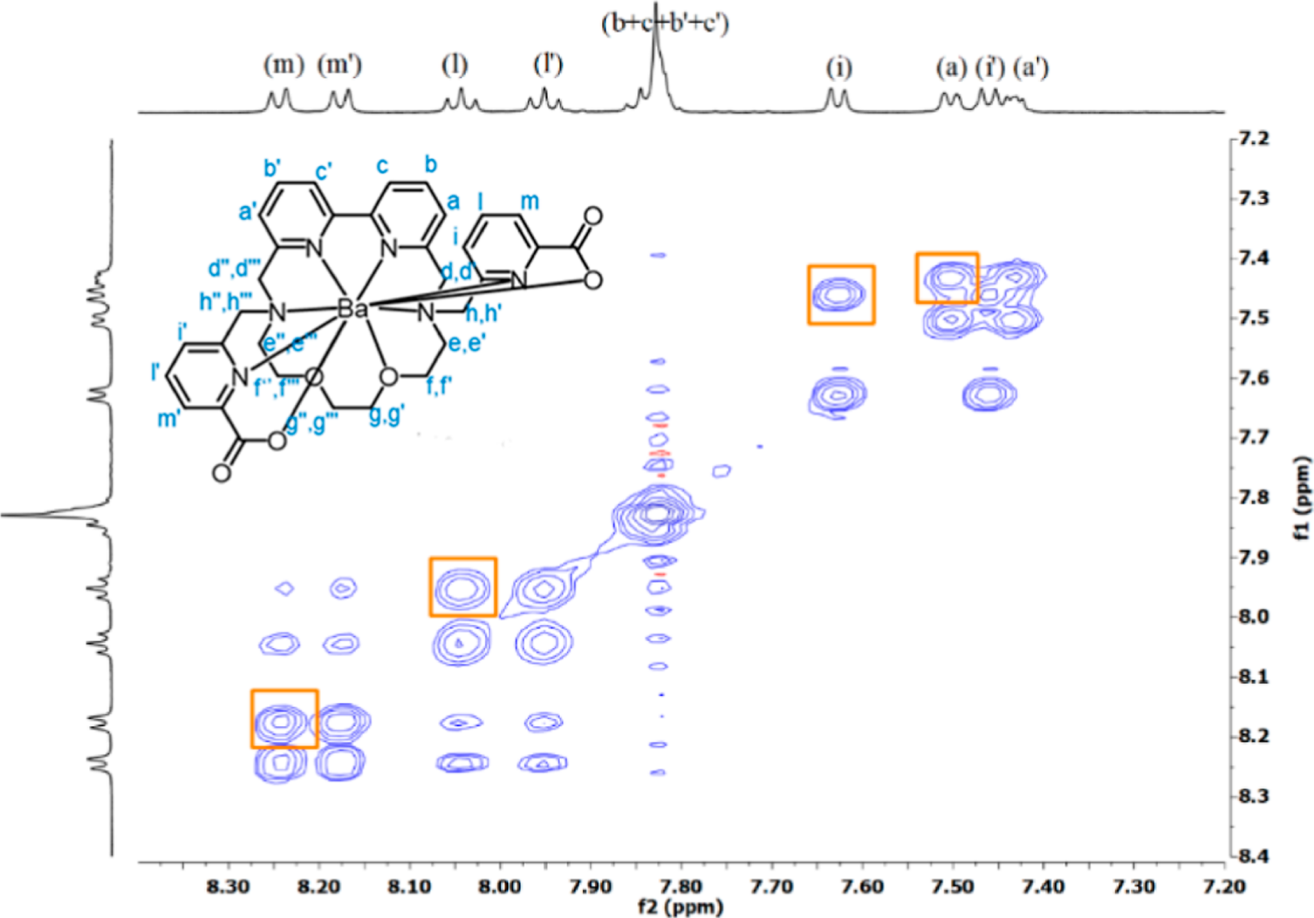
^1^H–^1^H ROESY spectrum (500 MHz) of the aromatic region of [Ba­(**bpycropa**)] (**4**) in CD_3_OD. Mixing time = 300 ms. Exchange cross peaks are evidenced by orange frames.

It was hypothesized that this phenomenon arises from the physical exchange of the coordinative positions of the two picolinate pendant arms, possibly through their dissociation/recomplexation, that leads to a Δ ↔ Λ inversion in the conformation of the complex and thus to an exchange of all pairwise chemically equivalent (but magnetically inequivalent) protons. To confirm this hypothesis, we performed DFT calculations (see computational details below). A careful conformational analysis of the [Ba­(**bpycropa**)] system afforded a minimum energy structure that corresponds to the Λ­(λδλ)/Δ­(δλδ) enantiomeric pair, whereas the conformation observed in the solid state (the Λ­(δδλ)/Δ­(λλδ) enantiomeric pair) displays a relative free energy of +9.08 kJ mol^–1^. The dynamic process observed in solution very likely corresponds to the Λ­(λδλ) ↔ Δ­(δλδ) racemization process, which involves the inversion of three chelate rings and the inversion of the configuration of the pendant arms.

The inversion of the N3–O1–O2–N4 aliphatic chain of the ligand follows a three-step process, with each step involving the inversion of one of the three five-membered chelate rings, leading to a Δ­(δλδ) ↔ Δ­(λδλ) interconversion. Figure 7 (left) shows an energy diagram for this interconversion pathway, as well as a representation of the λ and δ conformations of the central O1–O2 chelate ring. The transition state with the highest free energy is +47.8 kJ mol^–1^ above that of the Δ­(δλδ) isomer. This energy barrier is somewhat lower than those reported for the inversion of the macrocyclic unit in complexes of the trivalent lanthanide ions (60–65 kJ mol^–1^).^28^ This suggests that the lower positive charge density on Ba­(II) facilitates the inversion of the macrocyclic unit.

**Figure 7:**
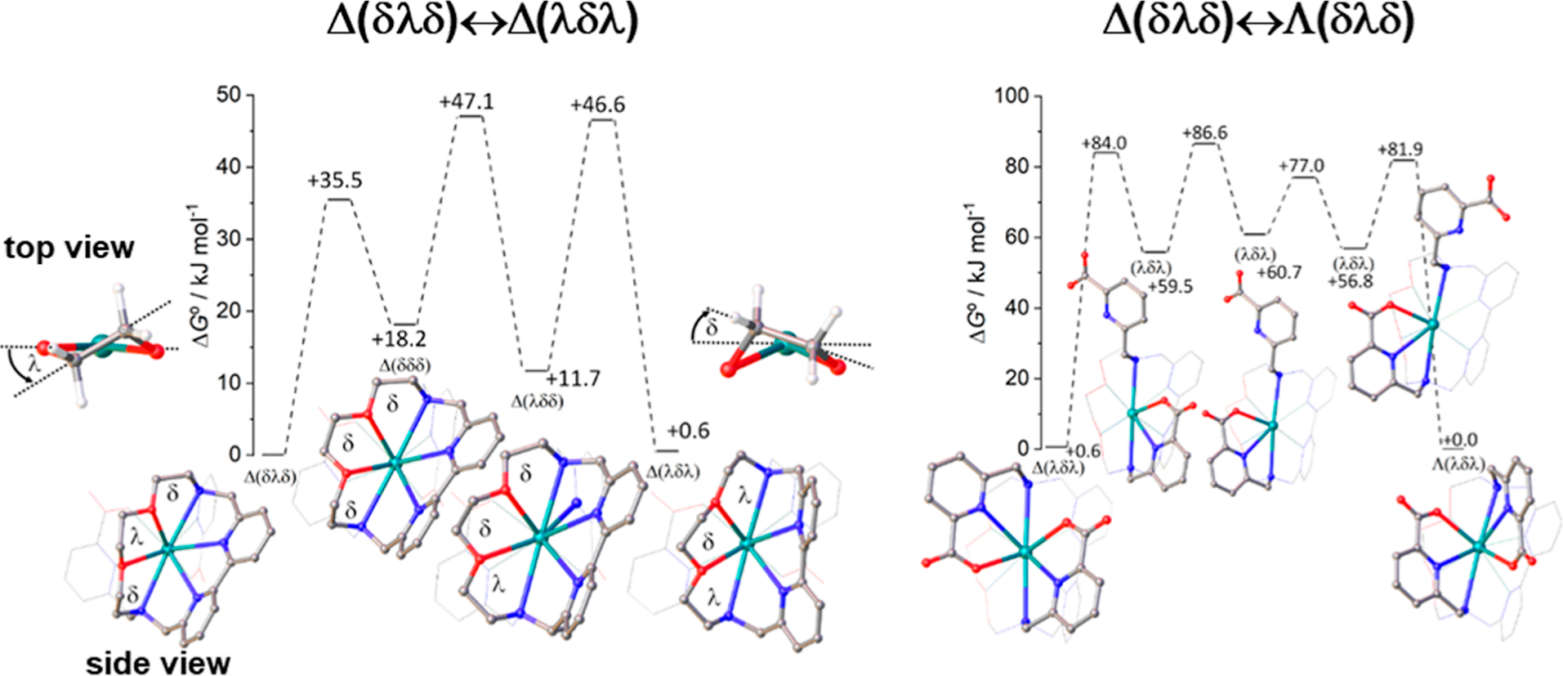
Left: Δ­(δλδ) ↔ Δ­(λδλ) interconversion process in [Ba­(**bpycropa**)] studied with DFT calculations and schematic representation of the λ and δ conformations of the central five-membered chelate ring. The energies of minima and transition states are given in kJ mol^–1^ (left). Right: Δ­(δλδ) ↔ Λ­(δλδ) interconversion process in [Ba­(**bpycropa**)] studied with DFT calculations. Dissociation of the picolinate moiety *trans* to bpy is represented. The energies of minima and transition states are given in kJ mol^–1^.

The change of the helicity of the pendant arms was also studied using DFT calculations (Figure 7, right). All attempts to find a nondissociative concerted pathway leading to a Δ ↔ Λ interconversion failed, which suggests that this process requires the dissociation of at least one of the picolinate arms. Our calculations indicate that picolinate dissociation is associated with a rather high free energy penalty of +59 kJ mol^–1^, which is virtually identical for both. Once the arm is dissociated, a series of three steps is required to complete the Δ­(λδλ) ↔ Λ­(λδλ) interconversion: reorientation of the coordinated picolinate within the metal coordination sphere, rotation of the dissociated pendant arm followed by recoordination of the picolinate in the complementary position. The overall process possesses an energy barrier of +86.6 kJ mol^–1^, as estimated from the energy of the transition state with the highest energy. We note, however, that our calculations incorporated solvent effects using a polarized continuum, but the actual process may be assisted by the coordination of solvent molecules, which may result in an overestimation of the energy barrier by our DFT study. Nevertheless, using the Eyring expression, the energy barrier calculated here suggests that the inversion of the helicity of the pendant arms takes place on the second time scale. A similar energy barrier was estimated for the dissociation of the acetate groups in Ln­(III) complexes of DOTA, which also requires arm dissociation.^29^ Overall, the computational study demonstrates that arm dissociation in [Ba­(**bpycropa**)] provides a suitable pathway for the racemization of the complex evidenced by the ROESY spectra.

### Thermodynamic Studies of the Barium Complexes

The stability constants of the complexes formed by **bpycropa** or **phencropa** with Ba­(II) were determined by potentiometric titrations (Table 2).

**Table 2:** Stability Constants Values of the Ba­(II) Complexes With the Investigated Chelators in Water (*T* = 25 °C, *I* = 0.15 M NaNO_3_) Obtained by Fitting Potentiometric Data

equilibrium	**macropa** ^a^	**bpycropa**	**phencropa**
Ba + L ⇌ BaL	11.45 ± 0.04	9.72 ± 0.15	11.76 ± 0.08
Ba + L + H ⇌ BaHL	15.19 ± 0.02	14.15 ± 0.15	16.08 ± 0.08
Ba + L + 2 H ⇌ BaH_2_L	17.98 ± 0.07	–	–
pBa	11.6	10.0	11.7

a Stability constants determined by potentiometry at *T* = 25 °C and *I* = 0.2 M NaCl. Data taken from ref 31.

The measurements were performed under the same conditions employed for the free ligands, namely in 0.15 M NaNO_3_ and over the pH range 1.9–12.0, using a standardized 0.1 M NaOH solution as titrant. The experimental data were fitted using the protonation constants of the free macrocycles, and including the formation of the species ML, M­(HL), M­(H_2_L) (metal complex, mono-, diprotonated, respectively) to obtain the overall formation constants (logβ values) for each barium-ligand system (Table 2). This approach afforded an accurate characterization of the complexes across a wide pH range and allowed direct comparison of the relative binding affinity of Ba­(II) with **macropa** under the same conditions.

A direct comparison of the thermodynamic stability of the Ba­(II) complexes based solely on individual log*K* values is not straightforward, as differences in protonation states and complex speciation among the chelators can significantly affect these constants. To overcome this limitation, a more comprehensive and commonly used descriptor is the pM value, defined as −log­[M]_(free)_, which represents the equilibrium concentration of unbound metal ion under specified conditions. Higher pM values correspond to lower concentrations of free metal and therefore indicate greater overall thermodynamic stability. In this study, pBa values were calculated from the experimentally determined p*K*_a_ and logβ constants by assuming standard conditions of *C*_Ba_ = 10^–6^ M, C_L_ = 10^–5^ M (L = **macropa**, **bpycropa**, **phencropa**), and pH 7.4, which are widely adopted in the field (Figure 8).^30^

**Figure 8:**
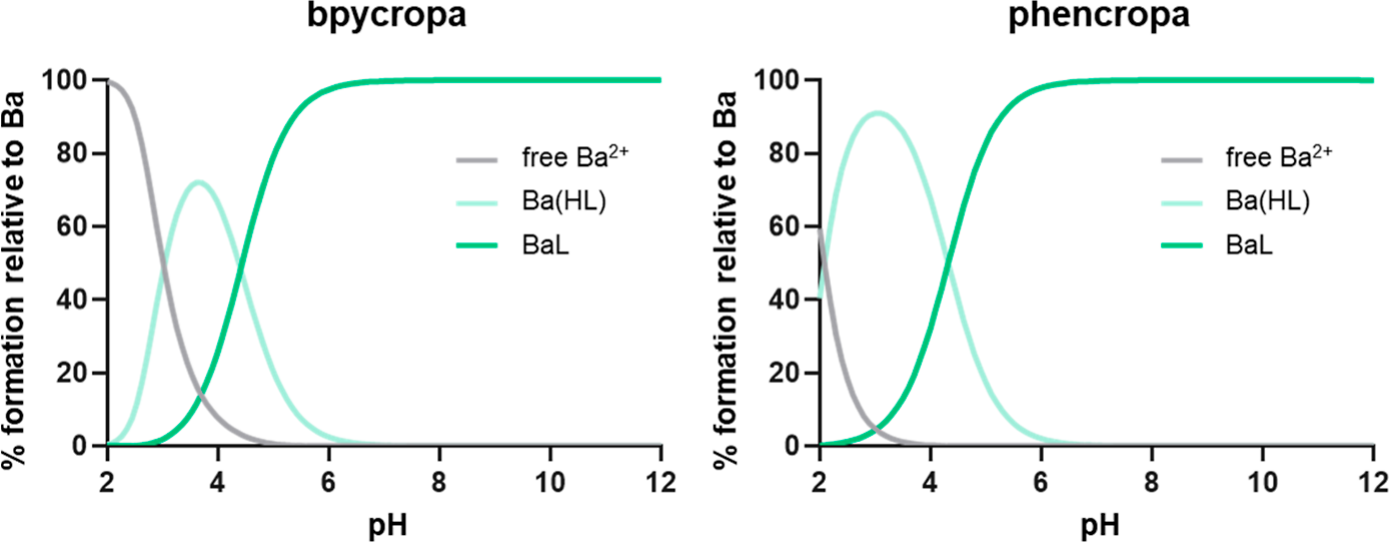
Distribution diagrams of Ba­(II) complexes with **bpycropa** (left) and **phencropa** (right) systems. p*K*_a_ and log*K* values were taken from Tables 1 and 2.

Based on the potentiometric data reported in Table 2, the barium complexes of **phencropa** and **macropa** display similar stability at pH 7.4 (pBa = 11.6 and 11.7, respectively), whereas the **bpycropa** complex is more than 1 order of magnitude less stable (pBa = 10.0). The difference between the **bpycropa** and **phencropa** complexes might be attributed to the distinct rigidity of the two aromatic units: the 2,2′-bipyridine in **bpycropa** is more torsionally flexible than the fused 1,10-phenanthroline in **phencropa**, which results in a less effective stabilization of the Ba­(II) complex. This trend is consistent with the generally higher stability constants reported for metal complexes of 1,10-phenanthroline compared to 2,2′-bipyridine.^32,33^

### Kinetic Inertness Studies

The kinetic inertness of **4** and **5** was evaluated through transmetalation experiments with LaCl_3_. It is worth noting that transmetalation (M + BaL → ML + Ba) and direct complex dissociation (BaL → Ba + L) are mechanistically distinct processes with potentially different kinetic profiles; Although transmetalation and direct dissociation probe different kinetic processes, transmetalation challenges with competing metal ions remain the standard methodology for evaluating the kinetic inertness of Ba­(II) complexes, thereby enabling direct comparison with previously reported **macropa** derivatives.^18,23^ The reactions were monitored by UV over 3 days, and the observed rate constants (*k*_obs_) and half-lives are summarized in Table 3 (see Experimental Section for details).

**Table 3:** Observed Dissociation Rate Constants *k*_obs_ and Half-Lives *t*_1/2_ for the Examined Complexes Determined via La­(III)-Mediated Transmetalations at pH = 7.4, 20 °C

	*k*_obs_ (s^–1^)	*t*_1/2_ (min)
[Ba(**macropa**)]	(5.50 ± 0.06) × 10^–4^	20.9 ± 0.3
[Ba(**bpycropa**)]	(1.62 ± 0.03) × 10^–4^	70.9 ± 1.5
[Ba(**phencropa**)]	(7.20 ± 0.05) × 10^–5^	160.7 ± 1.2

The progress of transmetalation was followed at λ = 284 nm for [Ba­(**macropa**)] and [Ba­(**phencropa**)], and at λ = 320 nm for [Ba­(**bpycropa**)]: the increasing absorbance corresponds to the formation of the respective lanthanum complexes (Figure S37).^35,36^

As summarized in Table 3, incorporating increasingly rigid aromatic moieties into the ligand backbone progressively enhances the kinetic inertness of the barium complexes. This trend is evident when comparing [Ba­(**bpycropa**)] and [Ba­(**phencropa**)], which exhibit half-lives of *t*_1/2_ = 70.9 min and *t*_1/2_ = 160.7 min, respectively. Both complexes are considerably more inert than [Ba­(**macropa**)] (*t*_1/2_ = 20.9 min), which lacks an aromatic backbone entirely, highlighting the role of rigid aromatic structures in improving the kinetic stability of barium complexes.

### Radiolabeling

Building on the promising results obtained with barium as a chemical surrogate for radium, both **bpycropa** and **phencropa** were evaluated for radium-223 complexation to assess their suitability for TAT applications. Radiolabeling studies were conducted under systematically varied conditions (ligand concentration, reaction time, temperature, and pH). **Macropa** was employed as a benchmark chelator for comparison. Radium-223 was provided by the PRISMAP project, and produced by the CERN-MEDICIS (Medical Isotopes Collected from ISOLDE) facility in Geneva using an isotope separator online (ISOL) device.

Under standard conditions (0.1 M ammonium acetate buffer, pH 6), radiolabeling of both **bpycropa** and **phencropa** showed a clear time dependence, with radiochemical conversions (RCCs) increasing between 5 and 30 min and reaching a plateau thereafter (see also Supporting Information, Figure S38). Efficient radiolabeling (RCC ≥95%) required relatively high ligand concentrations (6.7 × 10^–4^ M) and moderate heating (40 °C), conditions considerably more demanding than those reported for **macropa**, where RCC ≥95% is achieved at 10-fold lower ligand concentration and at 20 °C. Notably, increasing the reaction temperature to 90 °C proved detrimental rather than beneficial, leading to reduced RCCs, particularly at lower ligand concentrations, an effect only partially mitigated by raising the pH to 9.5, using 0.1 M sodium bicarbonate buffer (Figure 9). Lowering the pH had a detrimental effect, likely due to protonation of the nitrogen donor atoms of the ligands.

**Figure 9:**
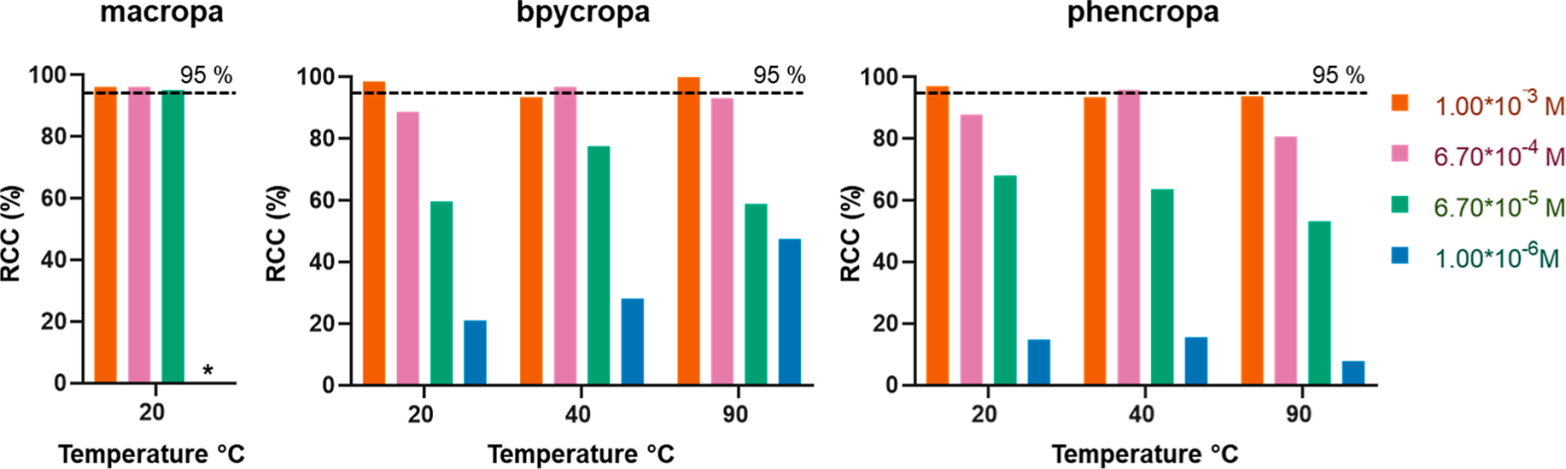
Concentration-dependent (*C*_L_ = 10^–3^–10^–6^ M) and temperature-dependent (20, 40, 90 °C) radiochemical incorporation by **macropa** (left), **bpycropa** (middle) and **phencropa** (right) of [^223^Ra]­RaCl_2_ (pH 6, *t* = 30 min; 2.7–15.3 kBq ^223^Ra). The dashed line indicates 95% RCC. * = not performed.

According to the partition coefficients, at pH 7.4 both [^223^Ra]­Ra-**bpycropa** (log*D*_7.4_ = −0.56 ± 0.04) and, in particular [^223^Ra]­Ra-**phencropa** (log*D*_7.4_ = 0.29 ± 0.01) are more lipophilic compared to [^223^Ra]­Ra-**macropa** (log*D*_7.4_ = −1.86 ± 0.01), which reflects the increasing contribution of aromatic moieties in the ligand scaffold. Decreased hydrophilicity has been suggested to reduce kidney absorbed dose in TAT,^34^ which may represent a future advantage of **phencropa**-based complexes over **macropa**.

The stability of the radiocomplexes [^223^Ra]­Ra-**bpycropa**, and [^223^Ra]­Ra-**phencropa** at 37 °C in EDTA medium (100 equiv) and human serum was studied and compared with that of [^223^Ra]­Ra-**macropa** under the same conditions. Each solution was analyzed at various time points (1, 5, 7, 12, and 19 days) by radio-TLC. Before beginning stability studies, each ligand was first radiolabeled using the previously established conditions: ligand concentration of 6.7 × 10^–4^ M, at 40 °C for 30 min.

In both EDTA and serum medium the radiochemical purity of [^223^Ra]­Ra-**bpycropa** and [^223^Ra]­Ra-**phencropa** decreased by more than 50% after 1 day, whereas [^223^Ra]­Ra-**macropa** was stable over 19 days.

Extensive radiolabeling studies performed on the Kryptofix-22-based chelator family, encompassing variants with different pendant arms and macrocyclic backbones, showed limited affinity toward radium-223.^15,16^ All the investigated ligands displayed poor or negligible radiochemical incorporation, with only a few systems achieving partial labeling yields under optimized conditions. Within this context, the ligands investigated in this work exhibit radiolabeling efficiencies that, while inferior to those of **macropa**, are superior to those reported for the majority of other Kryptofix derivatives.^10,15^ This finding supports the notion that picolinate moieties remain particularly effective donors for stabilizing Ra­(II). At the same time, the introduction of increased structural rigidity appears to slow down the complexation kinetics, consistent with reduced conformational adaptability. The lower stability of [^223^Ra]­Ra-**bpycropa** and [^223^Ra]­Ra-**phencropa** compared to [^223^Ra]­Ra-**macropa** under physiologically relevant conditions is in contrast with what is expected from the thermodynamic and kinetic data concerning the corresponding barium complexes. This discrepancy may reflect the intrinsic limitations of surrogate models for predicting radium-223 behavior, and/or the fact that Ba­(II) transmetalation experiments may not fully capture the kinetic inertness of radium-223 complexes under physiological conditions.

## Conclusions

Two new decadentate macrocyclic chelators, **bpycropa** and **phencropa**, were synthesized by incorporating rigid aromatic units (2,2′-bipyridine and 1,10-phenanthroline, respectively) into the Kryptofix 22 scaffold functionalized with picolinate pendant arms. Both ligands and their barium complexes were fully characterized by NMR spectroscopy, mass spectrometry, potentiometric titrations and X-ray crystallography. The solid state structures of [Ba­(**bpycropa**)] and [Ba­(**phencropa**)] are markedly different from that of [Ba­(**macropa**)],^23^ showing distorted asymmetric coordination environments with donor atoms organized in two approximately perpendicular planes, reflecting the conformational constraints imposed by the rigid aromatic units. The NMR data are fully consistent with the solid-state findings, indicating that the same structural features are maintained in solution. The asymmetric coordination environment allowed us to establish, through 2D NMR experiments, that in solution the two inequivalent picolinate moieties are involved in a dissociative exchange process that very likely corresponds to the Λ­(λδλ) ↔ Δ­(δλδ) racemization.

Thermodynamic and kinetic data demonstrate distinct differences in coordination behavior between the two chelators. Under physiological conditions, **phencropa** and **macropa** display comparable pBa values (11.7 and 11.6 respectively), whereas **bpycropa** exhibits a value lower by more than 1 order of magnitude (10.0). Kinetic inertness follows a similar trend: both new ligands form barium complexes that dissociate more slowly than **macropa**, with [Ba­(**bpycropa**)] and [Ba­(**phencropa**)] showing half-lives of 70.9 and 160.7 min, respectively, compared to 20.9 min for [Ba­(**macropa**)]. These encouraging results with barium, however, did not fully translate to radium. Although both chelators achieved quantitative radium-223 labeling, [^223^Ra]­Ra-**bpycropa** and [^223^Ra]­Ra-**phencropa** exhibited reduced serum stability relative to [^223^Ra]­Ra-**macropa**. These observations highlight the delicate balance between preorganization, kinetic accessibility, and thermodynamic stability in the design of chelators for radium radiochemistry. Moreover, the discrepancy between the encouraging barium results and the limited radium serum stability highlights that elements within the same group do not necessarily exhibit identical behavior, underscoring that surrogate studies, while valuable for mechanistic insight, cannot replace direct radiochemical validation.^14,37^ In this context, it should also be considered that transmetalation experiments, while standard practice for Ba­(II) systems, may not fully capture the kinetic inertness of radium-223 complexes under physiological conditions, representing an additional source of uncertainty in the interpretation of surrogate data.

Looking ahead, the results of this study, combined with the broader landscape of radium-223 chelation chemistry, suggest that the path toward improved chelators requires a more nuanced design strategy. The success of **macropa** points to the importance of a balanced donor set, combining hard oxygen donors with nitrogen atoms capable of providing both thermodynamic affinity and kinetic retention, rather than simply increasing rigidity, macrocyclic cavity size, or the number of a single donor type. Equally important is the systematic pairing of radiolabeling studies with full characterization of the corresponding Ba­(II) complexes. Developing reliable strategies to correlate the properties of Ba­(II) complexes with the behavior of the corresponding Ra­(II) species remains a central and open challenge for truly predictive chelator design.^38^ Although the chelators investigated in this study do not outperform **macropa** for the complexation of radium-223 under conditions relevant to targeted alpha therapy, they may still prove useful for the coordination of other radionuclides, such as terbium-161, actinium-225, or bismuth-212/213, as well as for selected lanthanides. Beyond their immediate applicability, the insights gained here advance the understanding of the structural features governing complex stability for this uniquely demanding class of cations, contributing to the ongoing pursuit of suitable chelating agents for heavy alkaline earth radiometals.

## Experimental Section

### Materials

All chemicals were of reagent grade and purchased from Sigma-Aldrich, except for 2,2′-bipyridine-6,6′-dicarbaldehyde and 1,10-phenanthroline-2,9-dicarbaldehyde, which were purchased from Fluorochem. Macrocycles **2bpy** and **2phen** were synthesized adapting a reported procedure for bipyridine-containing cryptates.^19,20^ Macropa and methyl 6-(chloromethyl)­pyridine-2-carboxylate were prepared according to literature protocols.^22,39^ Radium-223 (^223^Ra) was provided by the PRISMAP project, produced by the ISOLDE (Isotope Separator On Line DEvice) Radioactive Ion Beam Facility.

### Instrumental Methods

Mono- and bidimensional (^1^H–^1^H COSY, ^1^H–^13^C HSQC, ^1^H–^13^C HMBC, ^1^H–^1^H ROESY) NMR spectra were recorded at room temperature on a Varian 400, 500 or a Bruker 600 MHz spectrometer (^1^H: 400, 500, or 600 MHz, ^13^C: 100, 125, or 150 MHz). ^1^H chemical shifts in CDCl_3_ and CD_3_OD were referenced to the peak of residual nondeuterated solvent (δ = 7.26 and δ = 3.30 ppm, respectively), while the ^1^H chemical shifts in D_2_O were referenced to the peak of 2,2-dimethyl-2-silapentane-5-sulfonate sodium salt (DSS), used as an internal standard.

The mass spectra were obtained with the LTQ XL—Thermo Fisher mass spectrometer, while HR-MS experiments were carried out on a Bruker maXis mass spectrometer by the SALSA platform from ICOA laboratory or on a maXis UHR-Q-TOF mass spectrometer (Bruker, Bremen, Germany), with an electrospray ion source (ESI) in positive ionization mode.

UV–vis absorption spectra were recorded on an Agilent Cary 60 UV–vis spectrophotometer.

The pH of the analyzed solutions was measured with a sensION + PH31 (Hach) pH-meter equipped with a Mettler Toledo InLab NMR glass electrode.

### Synthesis

#### 2bpy

In a system kept under stirring at 40 °C and a continuous argon flow, 2,2′-bipyridine-6,6′-dicarbaldehyde (500 mg, 2.3 mmol, 1 equiv) was reacted with MgCl_2_ (448 mg, 4.7 mmol, 2 equiv) in anhydrous methanol (75 mL). After complete dissolution, a mixture consisting of 1,8-diamino-3,6-dioxaoctane (346 μL, *d* = 1.01 g/mL, 2.3 mmol, 1 equiv) in anhydrous methanol (9 mL) was added dropwise to the yellowish solution. The mixture was stirred for an additional hour to ensure full reaction of the amine. During this stage, a white-gray precipitate formed, which was removed by filtration. The solution was then reduced carefully with an excess of NaBH_4_ (446 mg, 11.5 mmol, 5.0 equiv), added in an ice bath, causing effervescence due to the release of *H*_2_(*g*), and stirred at room temperature for 12 h. The yellowish solution containing the reduced macrocycle was diluted with distilled water (60 mL) and the methanol was subsequently removed by rotary evaporation. The product was extracted from the aqueous solution with 3 × 10 mL portions of dichloromethane, and the organic phase was dried over Na_2_SO_4_ evaporated, yielding a yellow oil (476 mg, yield 62%) which was used for the next synthetic steps without further purification. ^1^H NMR (500 MHz, CDCl_3_, 298 K): δ_H_ 7.71 (t, *J* = 7.7, 2H_b_), 7.62 (d, *J* = 7.7, 2H_c_), 7.22 (d, *J* = 7.7, 2H_a_), 3.99 (s, 4H_d_), 3.67 (t, *J* = 4.6 Hz, 4H_f_), 3.57 (s, 4H_g_), 2.92 (t, *J* = 4.6 Hz, 4H_e_). ^13^C­{^1^H} NMR (126 MHz, CDCl_3_, 298 K): δ_C_ 157.5 (C1), 154.1 (C5), 148.9 (C2), 137.9 (C3), 123.9 (C4), 70.4 (C16), 70.1 (C15), 53.9 (C6), 48.1 (C14).

#### 3bpy

A 200 mg amount of **2bpy** (0.60 mmol, 1 equiv) was introduced in a 10 mL microwave vessel containing acetonitrile (4 mL), methanol (0.3 mL) and Na_2_CO_3_ (636 mg, 6 mmol, 10 equiv). Subsequently, 6-chloromethylpyridin-2-carboxylate methyl ester (226 mg, 1.2 mmol, 2 equiv) was added to the vessel. The reaction mixture was heated in a microwave reactor for 6 h at 110 °C. The solvent was removed using a rotary evaporator, and the resulting product was completely redissolved in dichloromethane (10 mL) and washed three times with distilled water. The organic phase was again evaporated to dryness and further dried under vacuum. A TLC plate of the crude product on alumina using a DCM/MeOH 95:5 eluent displayed two spots (*R*_f1_ = 0.58 and *R*_f2_ = 0.41). Therefore, purification by alumina column chromatography was conducted using a DCM/MeOH gradient. The second spot (*R*_f2_ = 0.41) was successfully isolated and identified as the desired product (172 mg, yield 45%). ^1^H NMR (500 MHz, CDCl_3_, 298 K): δ_H_ 7.87 (d, *J* = 7.7 Hz, 2H_*b*_), 7.84–7.73 (m, 6H_*c*,*l*,*m*_), 7.56 (d, *J* = 7.7 Hz, 2H_*a*_), 7.41 (d, *J* = 7.3 Hz, 2H_*i*_), 4.07 (s, 4H_*h*_), 3.88 (s, 6H, −CH_3_), 3.70 (t, *J* = 5.0 Hz, 4H_*f*_), 3.48 (s, 4H_*d*_), 2.85 (t, *J* = 5.0 Hz, 4H_*e*_), 2.67–2.57 (m, 4H_*g*_). ^13^C­{^1^H} NMR (126 MHz, CDCl_3_, 298 K): δ_*C*_ 163.8 (C13), 150.2 (C5), 149.3 (C1), 148.9 (C8), 147.3 (C12), 141.8 (C3), 140.4 (C10), 127.9 (C9), 127.1 (C2), 124.9 (C11), 123.3 (C4), 69.5 (C16), 65.0 (C15), 58.5 (C7), 57.9 (C6), 55.3 (−OCH_3_), 53.3 (C14) ppm.

#### bpycropa

A 172 mg amount of **3bpy** (0.2745 mmol, 1 equiv) was added to 6 M aqueous HCl (10 mL). The yellow solution was left under magnetic stirring and reflux at 100 °C for a total of 12 h. Subsequently, the acid aqueous solution product was concentrated to dryness using a rotary evaporator. **bpycropa** (6,6′-(7,10-dioxa-4,13-diaza-1,2­(2,6)-dipyridinacyclotetradecaphane-4,13-diylbis­(methylene))­dipicolinic acid) was obtained as a yellowish oil and was further purified by precipitation by dissolution in a minimal amount of water and precipitated by slowly adding acetone. **bpycropa** was obtained as a white-yellowish solid (117 mg, yield 71%). Solubility in water: 24.7 mg/mL. ^1^H NMR (500 MHz, CD_3_OD, 298 K): δ_*H*_ 8.40 (d, *J* = 7.9 Hz, 2H_*c*_), 8.28 (t, *J* = 7.8 Hz, 2H_*b*_), 8.17 (d, *J* = 7.7 Hz, 2H_*m*_), 8.10 (t, *J* = 7.8 Hz, 2H_*l*_), 7.95 (d, *J* = 7.7 Hz, 2H_*a*_), 7.84 (d, *J* = 7.7 Hz, 2H_*i*_), 5.04 (s, 4H_*d*_), 4.83 (s, 4H_*h*_), 3.85 (pt, 4H_*f*_), 3.74 (pt, 4H_*e*_), 3.44 (s, 4H_*g*_). ^1^H NMR (500 MHz, D_2_O, 298 K): δ_H_ 7.98 (t, *J* = 7.8 Hz, 2H_*b*_), 7.88 (d, *J* = 8.2 Hz, 2H_*c*_), 7.75 (t, *J* = 7.7 Hz, 2H_*l*_), 7.70 (d, *J* = 7.7 Hz, 2H_*m*_), 7.60 (d, *J* = 7.7 Hz, 2H_*a*_), 7.46 (d, *J* = 7.6 Hz, 2H_*i*_), 4.76 (s, 2H_*d*_), 4.60 (s, 2H_*h*_), 3.95 (m, 6H), 3.71 (m, 11H). ^13^C­{^1^H} NMR from the HSQC spectrum (126 MHz, CD_3_OD, 298 K): δ_C_ 164 (C13), 151 (C5), 149 (C8), 149 (C1), 146 (C12), 141 (C3), 139 (C10), 128 (C9), 126 (C2), 125 (C11), 123 (C4), 69 (C16), 64 (C15), 58 (C7), 57 (C6). MS (ESI^+^, CH_3_OH): *m*/*z* (%) = 599.34 (100, [M + H]^+^), 300.33 (85, [M + 2H]^2+^), 637.30 (30, [M + K]^+^), 621.32 (25, [M + Na]^+^). MS (ESI^–^, CH_3_OH): *m*/*z* (%) = 597.32 (100, [M–H]^−^), 619.37 (40, [M – 2H + Na]^−^). HR-MS (ESI^+^, CH_3_OH): *m*/*z* calcd for C_32_H_34_N_6_O_6_: [M + H]^+^ 599.2613, found 599.2605.

#### 2phen

The synthetic procedure was analogous to that of **2bpy**. In this case the starting aldehyde material was 1,10-phenanthroline-2,9-dicarbaldehyde (499 mg, 2.1 mmol, 1 equiv), which reacted with MgCl_2_ (423 mg, 4.4 mmol, 2 equiv) at room temperature. Subsequently, 1,8-diamino-3,6-dioxaoctane (310 μL, 2.1 mmol, *d* = 1.01 g/mL, 1 equiv)­was added gradually and uniformly in small aliquots over the course of 6 h, to limit the formation of undesired byproducts (e.g., the 2 + 2 macrocycle resulting from the condensation of two molecules of 1,10-phenanthroline-2,9-dicarbaldehyde with two molecules of diamine). The obtained macrocycle appears as an orange solid (660 mg, yield 88%), and was used for the next synthetic step without further purification. ^1^H NMR (500 MHz, CDCl_3_, 298 K): δ_H_ 8.13 (d, *J* = 8.2 Hz, 2H_*b*_); 7.70 (s, 2H_*c*_); 7.48 (d, *J* = 8.1 Hz, 2H_*a*_), 4.22 (s, 4H_*d*_); 3.75 (t, *J* = 4.7 Hz, 4H_*f*_); 3.69 (s, 4H_*g*_); 2.98 (t, *J* = 4.9 Hz, 4H_*e*_) ppm. ^13^C­{^1^H} NMR (125 MHz, CDCl_3_, 298 K): δ_*C*_ 159.1 (C1), 145.7 (C6), 136.6 (C3), 127.9 (C4), 126.0 (C5), 122.6 (C2), 70.7 (C16), 70.5 (C17), 55.5 (C15), 49.5 (C7) ppm.

#### 3phen

The synthesis was performed as for **3bpy**. **2phen** (198 mg, 0.56 mmol, 1 equiv) was introduced in a 10 mL microwave vessel with acetonitrile (4 mL), methanol (0.3 mL) and Na_2_CO_3_ (594 mg, 5.6 mmol, 10 equiv). Subsequently, 6-chloromethylpyridin-2-carboxylate methyl ester (209 mg, 1.124 mmol, 2 equiv) were added to the vessel. The reaction mixture was heated in a microwave reactor for 6 h at 110 °C. After evaporation and drying under vacuum, **3phen** was obtained (188 mg, yield 51%). A TLC plate on alumina using a DCM/MeOH 95:5 eluent displayed a single spot with an *R*_f_ = 0.47, corresponding to the alkylated product as confirmed by ^1^H NMR spectroscopy, hence no further purification was required. ^1^H NMR (500 MHz, CDCl_3_, 298 K): δ_H_ 8.20 (d, *J* = 8.2 Hz, 2H_*b*_), 8.01 (m, 2H_*m*,_ 2H_*i*_), 7.84 (t, *J* = 7.7 Hz, 2H_*l*_), 7.77 (s, 2H_*c*_); 7.72 (d, *J* = 8.2 Hz, 2H_*a*_), 4.31 (s, 4H_*d*_), 4.19 (s, 4H_*h*_), 4.01 (s, 6H, −CH_3_), 3.89 (t, *J* = 6.5 Hz, 4H_*f*_), 3.66 (s, 4H_*g*_), 2.86 (t, *J* = 6.5 Hz, 4H_*e*_) ppm. ^13^C­{^1^H} NMR (125 MHz, CDCl_3_, 298 K): δ_C_ 163.2 (C14), 149.5 (C1), 147.1 (C13), 142.9 (C6), 138.8 (C11), 138.8 (C4), 127.2 (C10), 138.1 (C3), 126.8 (C5), 124.8 (C2), 124.2 (C12), 70.2 (C17), 65.9 (C16), 59.4 (C7), 58.0 (C8), 56.3 (−OCH_3_), 55.2 (C15) ppm.

#### phencropa

The synthesis was conducted in an analogous way to **bpycropa**. Moreover, after the 12 h of stirring under reflux in HCl 6 M, a color change was observed, i.e. the solution shifted from orange to brown. **Phencropa** (6,6′-(6,9-dioxa-3,12-diaza-1­(2,9)-phenanthrolinacyclotridecaphane-3,12-diylbis­(methylene))­dipicolinic acid) was precipitated by adding diethyl ether dropwise to its concentrated methanol solution. The isolated product was obtained as a brown solid (166 mg, yield 82%). Solubility in water: 22.2 mg/mL. ^1^H NMR (500 MHz, CD_3_OD, 298 K): δ_H_ 8.29 (d, *J* = 8.2 Hz, 2H_*b*_), 7.79 (s, 2H_*c*_), 7.68 (d, *J* = 8.2 Hz, 2H_*a*_), 7.55 (d, *J* = 7.6 Hz, 2H_*m*_), 7.42 (t, *J* = 7.6 Hz, 2H_*l*_), 7.30 (d, *J* = 7.6 Hz, 2H_*i*_), 4.33 (s, 4H_*d*_), 3.84 (s, 4H_*h*_), 3.61 (t, *J* = 4.7 Hz, 4H_*f*_), 3.32 (s, 4H_*g*_), 3.06 (t, *J* = 4.7 Hz, 4H_*e*_) ppm. ^1^H NMR (500 MHz, D_2_O, 298 K): δ_H_ 8.47 (d, *J* = 8.2 Hz, 2H_*b*_), 7.92 (s, 2H_*c*_), 7.83 (d, *J* = 8.2 Hz, 2H_*a*_), 7.50 (t, *J* = 7.7 Hz, 2H_*m*_), 7.46 (d, *J* = 7.6 Hz, 2H_*l*_), 7.26 (d, *J* = 7.5 Hz, 2H_*i*_), 5.07 (s, 4H_*d*_), 4.73 (s, 4H_h_), 4.17 (t, *J* = 4.6 Hz, 4H_*f*_), 3.94 (m, 4H_*g*_, 4H_*e*_) ppm. ^13^C­{^1^H} NMR from the HSQC spectrum (125 MHz, CD_3_OD, 298 K): δ_C_ 165 (C14), 150 (C1), 147 (C13), 143 (C6), 139 (C11), 139 (C4), 127 (C10), 138 (C3), 127 (C5), 124 (C2+C12), 70 (C17), 65 (C16), 59 (C7), 58 (C8), 54 (C15) ppm. MS (ESI^+^, CH_3_OH): *m*/*z* (%) = 312.32 (100, [M + 2H]^2+^), 331.29 (51, [M + H + Na]^2+^), 623.32 (35, [M + H]^+^), 661.25 (24, [M + K]^+^), 645.28 (13, [M + Na]^+^), 339.23 (13, [M + H + K]^2+^). MS (ESI^–^, CH_3_OH): *m*/*z* (%) = 621.30 (100, [M–H]^−^), 325.18 (45, [M–2H]^2–^), 643.28 (37, [M–2H + Na]^−^). HR-MS (ESI^+^, CH_3_OH): *m*/*z* calcd for C_34_H_36_N_6_O_6_: [M + H]^+^ 623.2601, [M + Na]^+^ 645.2421, [M+2H]^2 +^ 312.1345; found [M + H]^+^ 623.2602, [M + Na]^+^ 645.2423, [M+2H]^2 +^ 312.1345.

#### [Ba­(**bpycropa**)] (**4**)

The synthesis of the complex was carried out by dissolving in a vial a 17.1 mg amount of **bpycropa** (0.025 mmol, 1 equiv) in water (2 mL). The aqueous ligand solution was set to pH 7 via additions of a KOH solution and monitored with a pH-meter. Subsequently, a 0.1 M BaCl_2_ solution (304 μL, 0.030 mmol, 1.2 equiv) was added. Shortly after adding the barium salt, a beige solid precipitated and was isolated by centrifugation. The solid is soluble in methanol and, depending on the pH, soluble in water as well. The detailed characterization and assignments of complex **4** are reported in the Results and Discussion section. Single crystals suitable for X-ray diffraction were obtained by slow evaporation of a methanol solution at room temperature. ^1^H NMR (500 MHz, CD_3_OD, 298 K): δ_H_ 8.25 (d, *J* = 8.0 Hz, 1H_*m*_), 8.18 (d, *J* = 8.0 Hz, 1H_*m*′_), 8.04 (t, *J* = 7.8 Hz, 1H_*l*_), 7.95 (t, *J* = 7.8 Hz, 1H_*l*″_), 7.87–7.79 (m, 4H_b/b′,c/c′_), 7.63 (d, *J* = 7.6 Hz, 1H_*i*_), 7.50 (d, *J* = 7.3 Hz, 1H_*a*_), 7.46 (d, *J* = 7.7 Hz, 1H_*i*′_), 7.43 (dd, *J* = 5.7, 3.0 Hz, 1H_*a*′_), 5.33 (d, *J* = 14.7 Hz, 1H_*d*_), 5.05 (pt, *J* = 13.5 Hz, 2H_*h*,*d*′_), 4.46 (td, *J* = 10.0, 2.4 Hz, 1H_*f*_), 4.04 (d, *J* = 13.6 Hz, 1H_*h*″_), 3.87 (d, *J* = 13.2 Hz, 1H_*h*′_), 3.78 (d, *J* = 13.9 Hz, 1H_*d*‴_), 3.68 (d, 1H_*d*′_), 3.56 (t, *J* = 9.4 Hz, 1H_*f*″_), 3.49 (d, *J* = 13.6 Hz, 1H_*h*‴_), 3.22 (dd, *J* = 10.1, 3.7 Hz, 1H_*f*′_), 3.18–3.08 (m, 2H_*e*,*f*″_), 3.04–2.85 (m, 4H), 2.83–2.75 (m, 1H), 2.72–2.59 (m, 2H). MS (ESI^+^, CH_3_OH): *m*/*z* (%) = 735.24 (100, [M + H]^+^), 367.99 (30, [M + 2H]^2+^). ^13^C­{^1^H} NMR (125 MHz, CD_3_OD, 298 K): δ_C_ 160.3 (C13), 160.1 (C13′), 159.7 (C8), 159.6 (C8′), 157.5 (C1), 157.2 (C1′), 155.8 (C12), 155.6 (C12′), 154.1 (C5′), 153.6 (C5), 121.7 (C11), 121.6 (C11′), 139.3 (C10), 138.9 (C10′), 138.6 (C4), 138.3 (C4′), 125.1 (C9), 125.3 (C9′), 124.1 (C3), 124.7 (C3′), 123.9 (C2), 122.3 (C2′), 61.1 (C6), 64.6 (C7), 63.7 (C15), 68.2 (C16), 64.5 (C7′), 63.6 (C15′), 61.1 (C6′), 68.4 (C16′), 69.5 (C17), 70.3 (C17′) ppm. HR-MS (ESI^+^, CH_3_OH): *m*/*z* calcd for C_32_H_34_BaN_6_O_6_: [M + H]^+^ 735.1514, [M + 2H]^2 +^ 368.0790; found [M + H]^+^ 735.1515, [M + 2H]^2 +^ 368.0789.

#### [Ba­(**phencropa**)] (**5**)

The synthesis was performed analogously to complex **4**, by dissolving **phencropa** (13.8 mg, 0.019 mmol, 1 equiv), setting the pH with KOH at 7 and mixing with a 0.1 M BaCl_2_ solution (238 μL, 0.023 mmol 1.2 equiv). As for complex **4**, after mixing the two solutions a yellow solid formed, which was isolated by centrifugation. The solid is soluble in methanol and, depending on the pH, soluble in water as well. Single crystals suitable for X-ray diffraction were obtained by slow evaporation of a methanol solution at room temperature. ^1^H NMR (500 MHz, CD_3_OD, 298 K): δ_H_ 8.53 (d, *J* = 8.2 Hz, 1H_*b*_), 8.43 (d, *J* = 8.2 Hz, 1H_*b*′_), 7.95–7.81 (m, 7H_*c*/*c*′,*a*,*l*′,*m*/*m*′,*i*_), 7.73 (d, *J* = 8.2 Hz, 1H_*a*′_), 7.55 (t, 1H_*l*_), 7.47 (d, *J* = 6.6 Hz, 1H_*i*′_), 5.36 (d, *J* = 14.7 Hz, 1H_*h*_), 5.27 (d, *J* = 13.3 Hz, 1H_*d*_), 5.15 (d, *J* = 13.7 Hz, 1H_*h*″_), 4.41 (t, *J* = 9.1 Hz, 1H_*f*_), 4.17 (d, *J* = 13.6 Hz, 1H_*d*″_), 4.02 (d, *J* = 13.3 Hz, 1H_*d*′_), 3.86 (d, *J* = 13.7 Hz, 1H_*h*‴_), 3.76 (d, *J* = 14.7 Hz, 1H_*h*′_), 3.64 (d, *J* = 13.7 Hz, 1H_*d*‴_), 3.47 (t, *J* = 9.0 Hz, 1H_*f*″_), 3.17–3.01 (m, 2H_*f*′,*e*_), 2.97 (dd, *J* = 18.4, 10.7 Hz, 2H_*f*‴,*g*_), 2.89–2.77 (m, 2H_*e*,*e*‴_), 2.70–2.60 (m, 2H_*g*′,*e*‴_), 2.54 (t, *J* = 9.8 Hz, 2H_*g*″/*g*‴_). ^13^C­{^1^H} NMR (125 MHz, CD_3_OD, 298 K): δ_C_ 160.7 (C1), 160.6 (C1′), 160.3 (C6′), 160.1 (C6), 157.8 (C9), 157.6 (C9′), 153.8 (C13 + C13′), 145.7 (C4+C4′), 138.8 (C3), 138.3 (C3′), 138.4 (C12), 138.2 (C12′), 126.5 (C5+C5′), 124.7 (C11), 124.4 (C10′), 124.5 (C2′), 123.7 (C2), 122.5 (C11′+C10), 70.2 (C17), 69.5 (C16′), 69.4 (C17′), 68.2 (C16), 64.8 (C7+C7′), 63.8 (C8′), 61.1 (C8), 56.5 (C15), 56.4 (C15′) ppm. MS (ESI^+^, CH_3_OH): *m*/*z* (%) = 759.34 (100, [M + H]^+^), 380.02 (42, [M + 2H]^2+^). HR-MS (ESI^+^, CH_3_OH): *m*/*z* calcd for C_34_H_36_N_6_O_6_: [M + H]^+^ 759.1508; found 759.1508.

#### Potentiometric Titrations

The stability and protonation constants of the macrocycles and complexes were determined by pH-potentiometry. The titrated solutions contained metal and ligand in a 1:1 molar ratio (ligand concentration typically 2 mM) and were carried out with a Metrohm Toledo’s Titration Excellence T5 automatic workstation equipped with a pH S-Micro DGi102 mini (Metrohm) electrode. Measurements were carried out at 298 K in 6 mL samples with continuous magnetic stirring, maintaining a constant ionic strength of 0.15 M NaNO_3_. The pH range investigated was 1.9–12.0. A waiting time of 60 s between consecutive measurements was used for the ligands, while a longer interval of 110 s was applied for the complexes to ensure that equilibrium was reached. In the equilibrium calculations, the stoichiometric ionic product of water (p*K*_w_) was also considered for the determination of [OH^–^] under basic conditions. To account for systematic errors in the determination of [H^+^] at extreme pH values, the method reported by Irving et al. was employed.^40^ Briefly, a blank titration of a solution of known HCl concentration at fixed ionic strength was performed under the same experimental conditions, and the resulting data were used to correct for electrode junction potential contributions prior to fitting. The protonation and stability constants were fitted using Hyperquad2008 software.

#### ^1^H NMR Titrations

For the ^1^H NMR titrations of **bpycropa** and **phencropa**, a vial containing respectively 1 mL of a 0.01 M solution of the ligand, 0.15 M NaNO_3_ and 0.001 M of 2,2-dimethyl-2-silapentane-5-sulfonate sodium salt (DSS), which was used as reference at 0.0 ppm in D_2_O, was prepared. The ^1^H NMR spectra (500 MHz) were recorded at 298 K, with each spectrum acquired at approximately 0.5 pH-unit intervals over a range between pH 1.3 and 12.1. The pH of the solutions was adjusted through small additions of diluted D_2_SO_4_ and NaOD solutions in D_2_O.

#### X-ray Diffraction

Data collections were performed at the X-ray diffraction beamline (XRD1) of the Elettra Synchrotron of Trieste (Italy) equipped with a Pilatus 2 M image plate detector. Collection temperature was 100 K (nitrogen stream supplied through an Oxford Cryostream 700); the wavelength of the monochromatic X-ray beam was 0.700 Å and the diffractograms were obtained with the rotating crystal method. The crystals were dipped in N-paratone and mounted on the goniometer head with a nylon loop. The diffraction data were indexed, integrated and scaled using the XDS code.^41^ The structures were solved by the dual space algorithm implemented in the SHELXT code.^42^ Fourier analysis and refinement were performed by the full-matrix least-squares methods based on F^2^ implemented in SHELXL.^43^ The Coot and SHELXLE programs were used for modeling.^44,45^ Anisotropic thermal motion was allowed for all non-hydrogen atoms. Hydrogen atoms were placed at calculated positions with isotropic factors *U* = 1.2 × *U*_eq_, *U*_eq_ being the equivalent isotropic thermal factor of the bonded non hydrogen atom. Crystal data and details of refinements are in the ESI.

#### Computational Details

All DFT calculations were performed with the hybrid PBE0 ^46^ density functional and the Gaussian 16 ^47^ C.01 program package. Relativistic effects were considered with the use of the relativistic effective core potential ECP46MDF and the associated (13s12p6d4f2g)/[9s9p6d4f2g] valence basis set for Ba.^48^ The standard Def2-TZVP basis set was used for all other atoms.^49^ Empirical dispersion corrections were considered with the D3 method proposed by Grimme, incorporaing the damping scheme of Becke and Johnson (D3­(BJ)).^50^ The effects of the solvent were incorporated with the integral equation formalism of the polarized continuum model.^51^ The convergence of the self-consistent field procedure and the size of the integration grids were selected with the scf = tight and integral = superfinegrid keywords. Transition states were located using the synchronous transit and quasi-Newton method (QST3 method).^52^ All local energy minima and transition states were characterized using analytical second derivatives.

#### Kinetic Inertness Studies

The kinetic inertness of the two barium complexes (**4**) and (**5**) was studied by performing transmetalation reactions with LaCl_3_ and monitoring them by UV spectroscopy in the range 220–380 nm for 3 days at 20 °C. The concentration of the complexes in the reaction quartz cuvette was 60 μM, while the competing metal ion, La­(III), was in 100-fold excess (6 mM) to ensure pseudo-first-order conditions, thus being incorporated in the observed rate constant *k*_obs_ (eq 1), from which the respective half-lives were calculated with eq 2. Additionally, the assays were performed in aqueous solutions containing 10 mM HEPES buffer to maintain a constant pH of 7.4 and constant ionic strength *I* = 0.15 M with NaCl. The progress of transmetalation was followed at λ = 284 nm for [Ba­(**macropa**)] and [Ba­(**phencropa**)], and at λ = 320 nm for [Ba­(**bpycropa**)], where the increasing absorbance corresponds to the formation of the respective lanthanum complexes. The observed rate constants were obtained by fitting the kinetic data according to eq 3, in which the parameters *A*_t_, *A*_0_, and *A*_e_ are the absorbance values at time *t*, at the start of the reaction and at equilibrium, respectively.

1 $$-\frac{d\left[ML\right]}{dt}=k_{obs}\left[ML\right],,k_{obs}=k\left[La\left(III\right)\right]$$

2 $$t_{1/2}=\frac{\ln2}{k_{obs}}$$

3 $$A_{t}=\left(A_{0}-A_{e}\right)e^{-k_{obs}t}+A_{e}$$

in which the parameters *A*_*t*_, *A*_0_, and *A*_e_ are the absorbance values at time *t*, at the start of the reaction and at equilibrium, respectively.

#### Radiolabeling

*Caution! radium-223 is radioactive. It should be handled only by trained personnel in properly equipped facilities.*

Radium-223 (^223^Ra) was provided by CERN-MEDICIS (Medical Isotopes Collected from Isolde), produced as a high-purity sample by mass separation.^53^ At CERN-MEDICIS, ^223^Ra is produced through ^232^Th­(p,x) nuclear reactions in 20 cm long ThC_*x*_ refractory target material, induced with 1.4 GeV protons delivered by the CERN Proton Synchrotron Booster, followed by mass separation.^54^ Through this production process, other Ra radionuclides and spallation products are also produced and separated, such as Ra-224, Ra-225, Ra-226 and Ra-228; high radionuclidic purity Ra-223 can therefore only be obtained by physical mass separation and implantation along A/Z = 223 into a metal or salt-backed foil.

Mass separation uses a dipole magnet to deflect ionized and accelerated isotopes according to their mass-to-charge ratio through the Lorentz force, using chemically selective 1+ ion sources. By blocking unwanted neighboring trajectories with mechanical slits, beams of high radionuclidic purity can be obtained and implanted in a stopping foil.^55^ Typical mass separation efficiencies at CERN-MEDICIS range from 5–50%, with a notably high efficiency for Ra of up to ∼70% (decay-corrected). For this work, the Ra-223 mass separation efficiency over the two collections was 10 ± 1% (decay-corrected), reflecting the condition of the target units and the operating conditions selected for this run.

On mass 223, no elemental stable or radioactive contaminants are expected due to the short half-life of neighboring isobars. At CERN-MEDICIS, mass separation begins a few hours after irradiation, by which time most potential contaminants have decayed. Molecular ions such as oxides may in principle form, but their formation is hindered by the excess carbon in the target material and by the selective surface ion source used, and they are further suppressed by their high ionization potentials.^53^ Radiochemical impurities at mass 207 were detected in the gamma spectra. Other impurities may arise from neighboring mass tail bleeding into the desired trajectory, for instance from an intense Ra-224 beam and its decay products; this can be suppressed and optimized through narrower blocking slit positions.

^223^Ra was received as a cation implanted in NaCl backing on an aluminum foil. Radium-223 was recovered by dissolving the NaCl backing with 500 μL of HCl 0.1 M (metal-free). This stock solution was diluted with 0.01 M HCl (metal-free), to obtain an activity between 2.7 and 15.3 kBq for experiments, with a volume set at 10 μL of [^223^Ra]­RaCl_2_.

The three ligands’ solutions were prepared in ultrapure water, with concentrations from 10^–2^ M to 10^–5^ M. For radiolabeling experiments, 10, 15.6, 50, or 100 μL of ligand solution were added. The reaction medium was completed with 130 μL of 0.1 M ammonium acetate buffer at pH 6. To study the influence of the pH, some experiments were tested with different buffer solutions: 0.1 M sodium acetate buffer at pH 3.5, 0.1 M sodium acetate buffer at pH 4.65, 1.5 M HEPES buffer at pH 5, 0.1 M sodium acetate buffer at pH 5.2, 1 M HEPES buffer at pH 7.5, phosphate buffer at pH 9, and 0.1 M sodium bicarbonate buffer at pH 9.5. All buffers were purified on 5 g/L Chelex resin (100–200 mesh, Sigma-Aldrich). This gave a final ligand concentration from 10^–3^ M to 10^–6^ M. Radiolabeling experiments were conducted at different temperatures (20, 40, and 90 °C), with different reaction times (5, 30, and 60 min). All experiments were performed in triplicate.

Each radiolabeling was analyzed by radio-TLC by spotting reaction solution onto TLC sheets and developing them using mobile phases. Various TLC plates (aluminum-backed silica gel 60, iTLC-SG, iTLC-SA, Whatman 1 paper, DGA (diglycolamide) sheets, DGA isheets and TKI213 isheets, the latter 2 being generously provided by Triskem, Rennes, France) and mobile phases (50 mM EDTA, 0.4 M sodium citrate pH 6, 0.1 M sodium hydroxide, 0.1 M hydrogen chloride) were tested, to obtain the best quality control method, depending on the ligand tested. Best results were obtained when radiolabeled. **bpycropa** and **phencropa** were analyzed on iTLC-SG (instant thin layer chromatography impregnated with a silica gel) sheets with 0.1 M sodium hydroxide as eluent, and radiolabeled **macropa** on DGA (diglycolamide, Triskem International) impregnated TLC sheets with 0.1 M sodium hydroxide as eluent (see Figures S39–S44 for representative TLC plates and chromatograms). After elution, TLC sheets were wrapped in stretch film and, following an equilibration time of at least 6 h, were analyzed with a Cyclone Storage Phosphorimager (PerkinElmer) to determine the radiochemical conversion (RCC).

#### Determination of the Octanol/Water Partition Coefficient LogD_7.4_

The reaction conditions used for LogD_7.4_ determination were 40 °C for 30 min with a final ligand concentration of 6.7 × 10^–4^ M. These conditions were selected based on the lowest ligand concentration that gave nearly quantitative radiochemical conversions. 50 μL of [^223^Ra]­Ra-complexes were mixed with 1 mL of *n*-octanol and 1 mL of PBS. The solutions were vigorously shaken and centrifugated at 2700 rpm for 5 min. After separation of the phases, 100 μL of each phase were taken and counted with an AMG automatic gamma counter (Hidex). LogD_7.4_ was calculated as follows: LogD_7.4_ = log­[*A*_Oct_]/[*A*_PBS_], where *A*_Oct_ and *A*_PBS_ are the measured activities of the *n*-octanol and aqueous phase, respectively. Experiments were done in triplicate.

#### Stability Studies

The reaction conditions used for stability studies were 40 °C for 30 min with a final ligand concentration of 6.7 × 10^–4^ M. After radiolabeling, 100 μL of each radiocomplex were added to 700 μL of 0.01 M EDTA solution or 400 μL of an aliquot of human serum (sourced from a pool of donor, Biopredic) and incubated at 37 °C. Then, at various time points (1, 5, 7, 12, and 19 days), an aliquot of each sample was collected and analyzed by radio-TLC onto iTLC-SG sheets, with 0.1 M sodium hydroxide as eluent, or onto DGA sheets, with 0.1 M sodium hydroxide as eluent. After elution, TLC sheets were analyzed with a Cyclone Storage Phosphorimager to determine the radiochemical purity (RCP). Stability studies were performed in triplicate.

## Supplementary Material



## Notes

It contains the detailed NMR characterization of **bpycropa**, **phencropa** and **4**; NMR spectra of **2bpy**, **3bpy**, **2phen**, **3phen**, **bpycropa**, **phencropa**, **4**, **5**, crystallographic details of **4**, **5**; thermodynamic and kinetic studies of **bpycropa** and **phencropa**, radiolabeling kinetics of **bpycropa** and **phencropa**; DFT geometries of **4** (PDF)
