# Supplementary material for: Impact of Bipyridine and Phenanthroline Incorporation into the Macropa Scaffold on Ba(II)/Ra(II) Chelation
Source: Inorg Chem. 2026 Jul 15;65(29):17230–44. doi: 10.1021/acs.inorgchem.6c02747 (PMC13418164; doi:10.1021/acs.inorgchem.6c02747)
Supplement: Supplementary file 1 [file ic6c02747_si_001.pdf]

Supporting Information

Impact of Bipyridine and Phenanthroline Incorporation into the Macropa Scaffold on Ba(II)/Ra(II) Chelation

Ileana Merdžo,[1] Clarisse Brossard,[2] Nicolas Lepareur,[2] Carlos Platas-Iglesias,[3] Gabriele Balducci,[1] Raphaël Tripier[4]*, Enzo Alessio[1], and Federica Battistin[1,4]*

[1] University of Trieste, Department of Chemical and Pharmaceutical Sciences, Via L. Giorgieri 1, 34127 Trieste, Italy.
[2] Univ Rennes, Centre Eugène Marquis, Inrae, Inserm, Institut NUMECAN [(Nutrition, Métabolismes et Cancer)]—UMR_A 1341, UMR_S 1241, Avenue de la Bataille Flandres, Dunkerque CS 44229, 35042 Rennes Cedex, France.
[3] CICA - Centro Interdisciplinar de Química e Bioloxía and Departamento de Química, Universidade da Coruña, 15008 A Coruña, Spain.
[4] Univ Brest, UMR-CNRS 6521 CEMCA, 6 avenue Victor le Gorgeu, 29238 Brest, France.
Emails: federica.battistin@units.it, raphael.tripier@univ-brest.fr

Table of Contents:

1. NMR characterization: 3

1.1 Bpycrown (2bpy) 3

1.2 Bpycrown-*N,N′*-dipicolinate methyl ester (3bpy) 4

1.3 Phencrown (2phen) 5

1.4 Phencrown-*N,N′*-dipicolinate methyl ester (3phen) 6

1.5 Bpycropa 8

1.6 Phencropa 12

1.7 [Ba(bpycropa)] (4) 16

1.8 [Ba(phencropa)] (5) 18

2. Thermodynamic studies: ^{1}H NMR titrations of bpycropa and phencropa 22

3. Kinetic inertness studies 26

4. Radium-223 labelling 27

4.1 Radio-TLC plates and chromatograms 28

5. X-ray Crystallography 32

6. ESI-MS spectra 34

7. HR-ESI-MS spectra 37

8. DFT geometries 42

8.1 Sample input files 42

8.2 DFT optimized geometries 54

1. NMR characterization:

1.1 Bpycrown (2bpy)

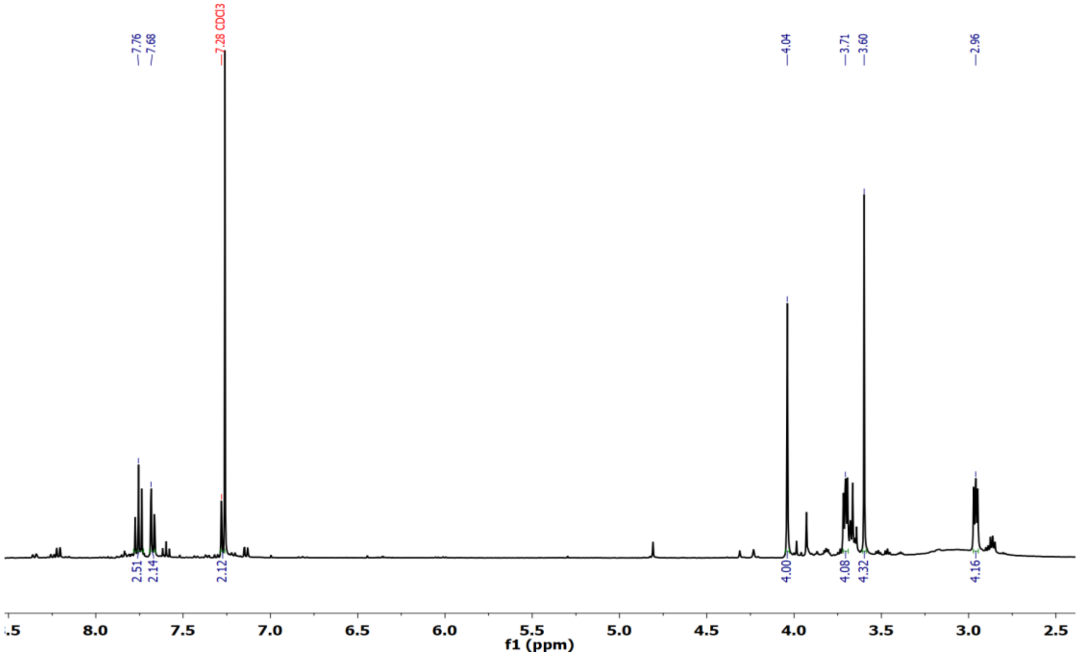

Figure S1. ^{1}H NMR spectrum (400 MHz) of **bpycrown** (**2bpy**) in $CDCl_3$.

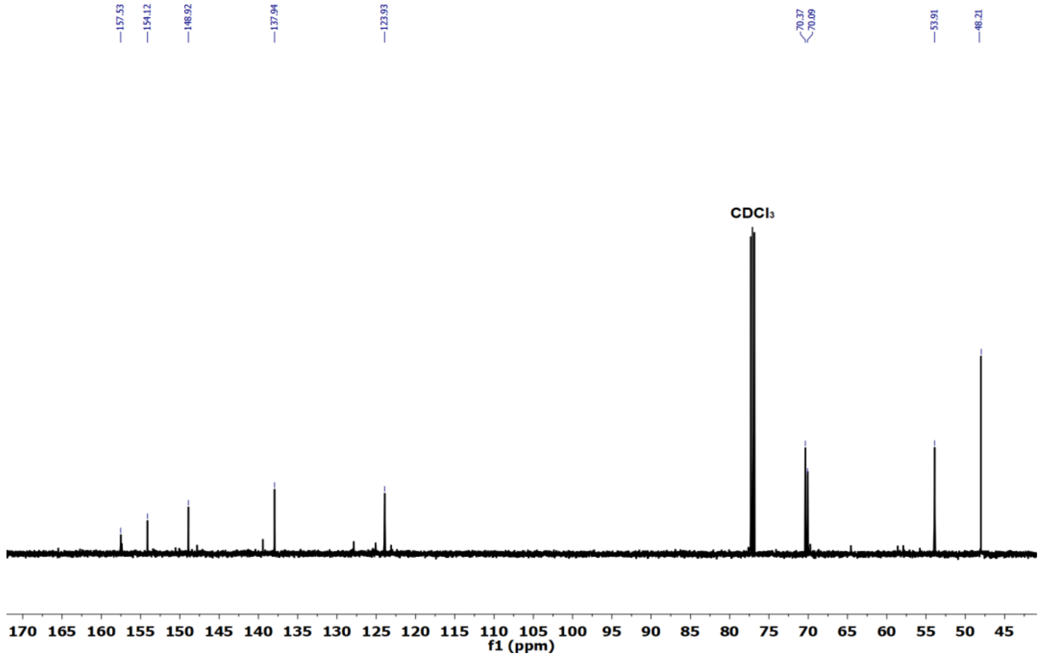

Figure S2. ^{13}C NMR spectrum (126 MHz) of **bpycrown** (**2bpy**) in $CDCl_3$.

1.2 Bpycrown-*N,N′*-dipicolinate methyl ester (3bpy)

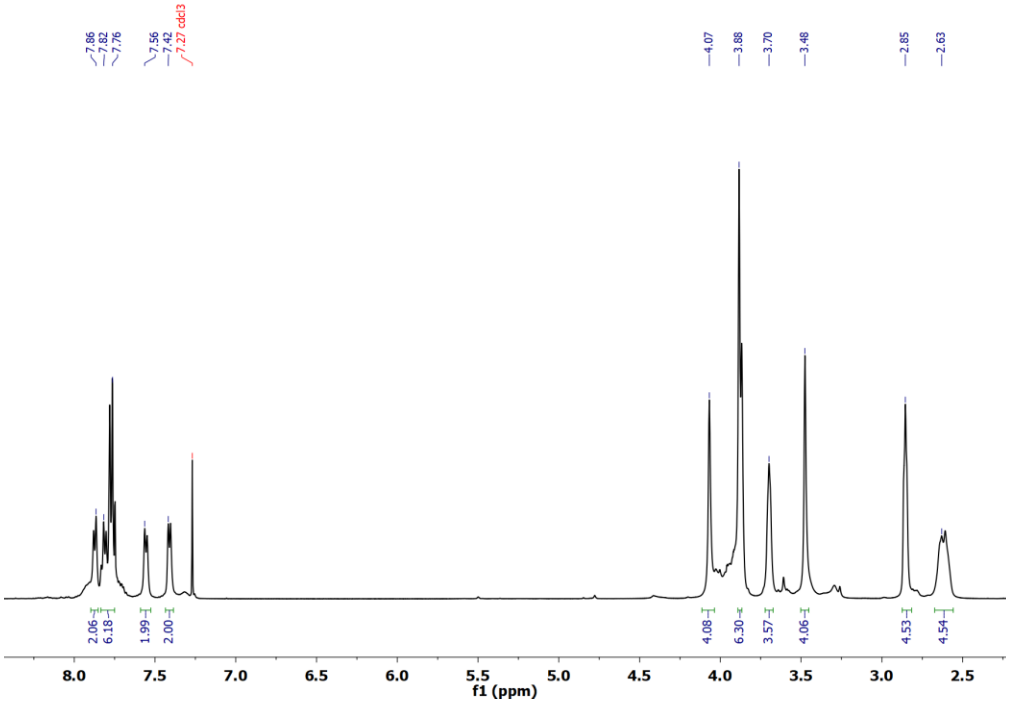

Figure S3. ^{1}H NMR spectrum (500 MHz) of bpycrown-*N*,*N′*-dipicolinate methyl ester (**3bpy**) in $CDCl_3$.

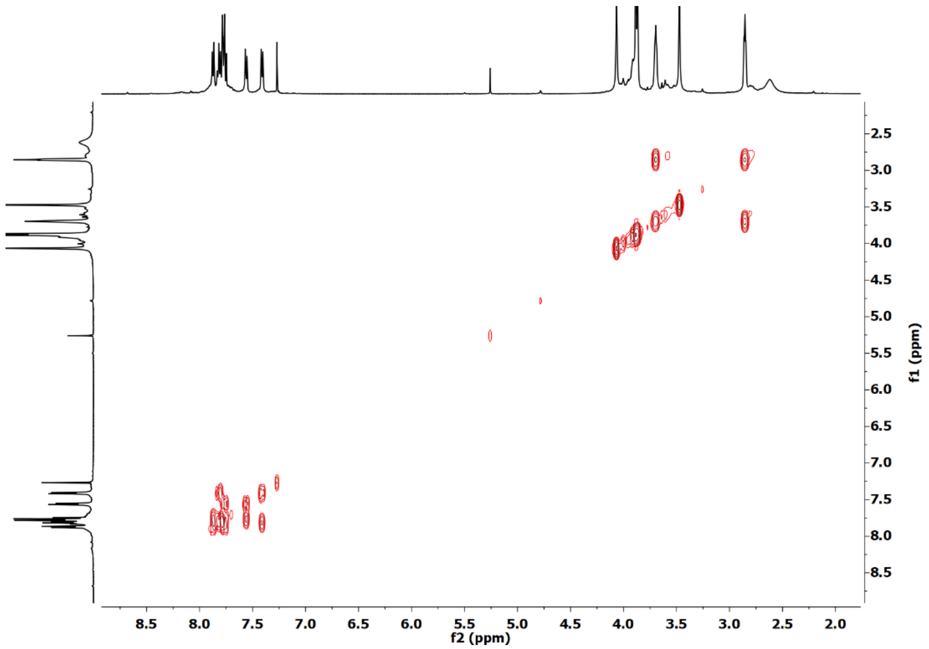

Figure S4. ^{1}H – ^{1}H COSY spectrum (500 MHz) of bpycrown-*N,N′*-dipicolinate methyl ester (**3bpy**) in $CDCl_3$.

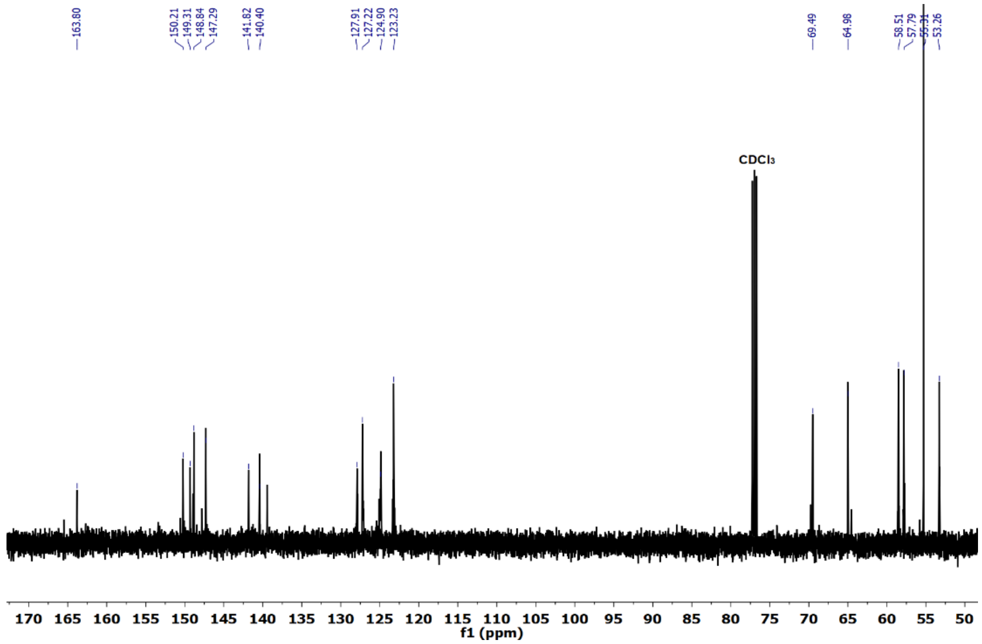

Figure S5. $^{13}C\{^{1}H\}$ NMR spectrum (500 MHz) of bpycrown-*N,N'*-dipicolinate methyl ester (**3bpy**) in $CDCl_3$.

1.3 Phencrown (2phen)

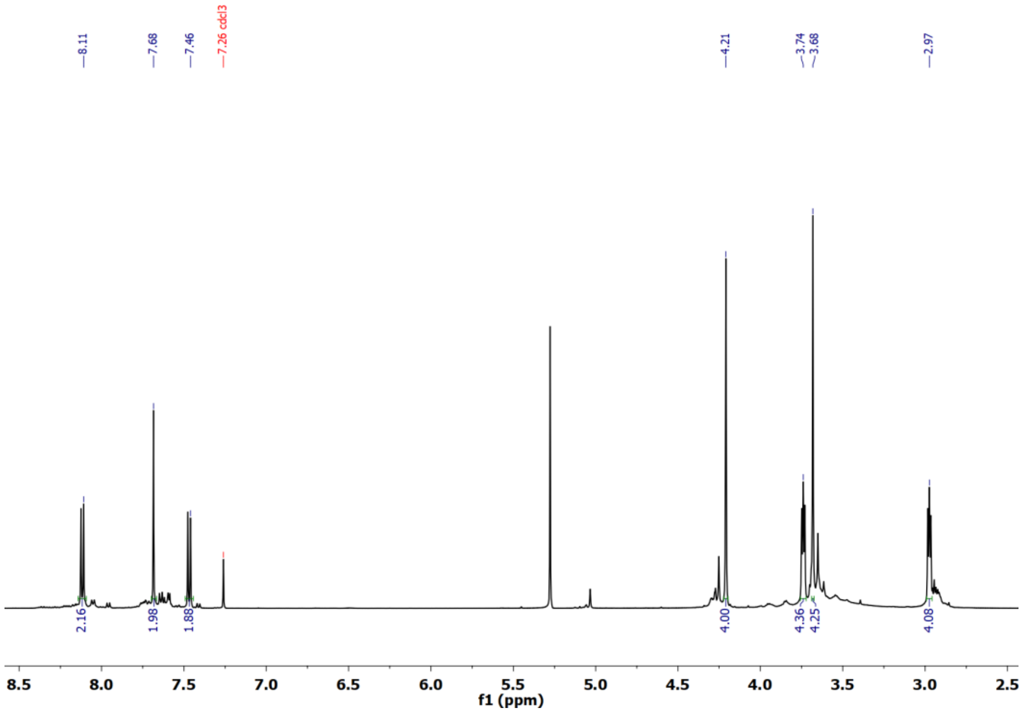

Figure S6. ^{1}H NMR spectrum (500 MHz) of **phencrown** (**2phen**) in $CDCl_3$

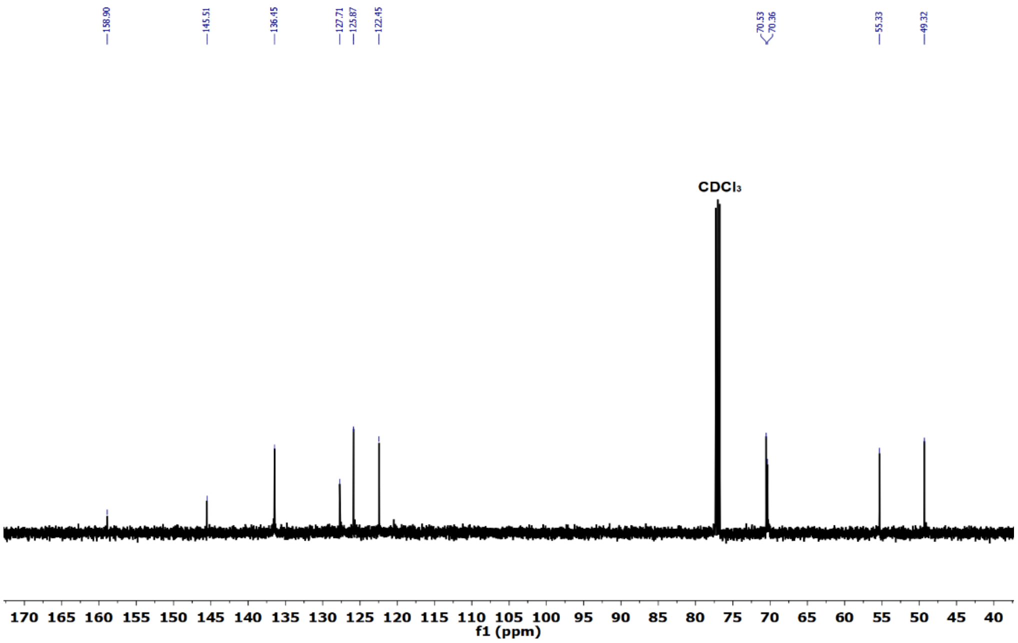

Figure S7. ^{13}C NMR spectrum (126 MHz) of **phencrown (2phen)** in $CDCl_3$.

1.4 Phencrown-*N*,*N′*-dipicolinate methyl ester (3phen)

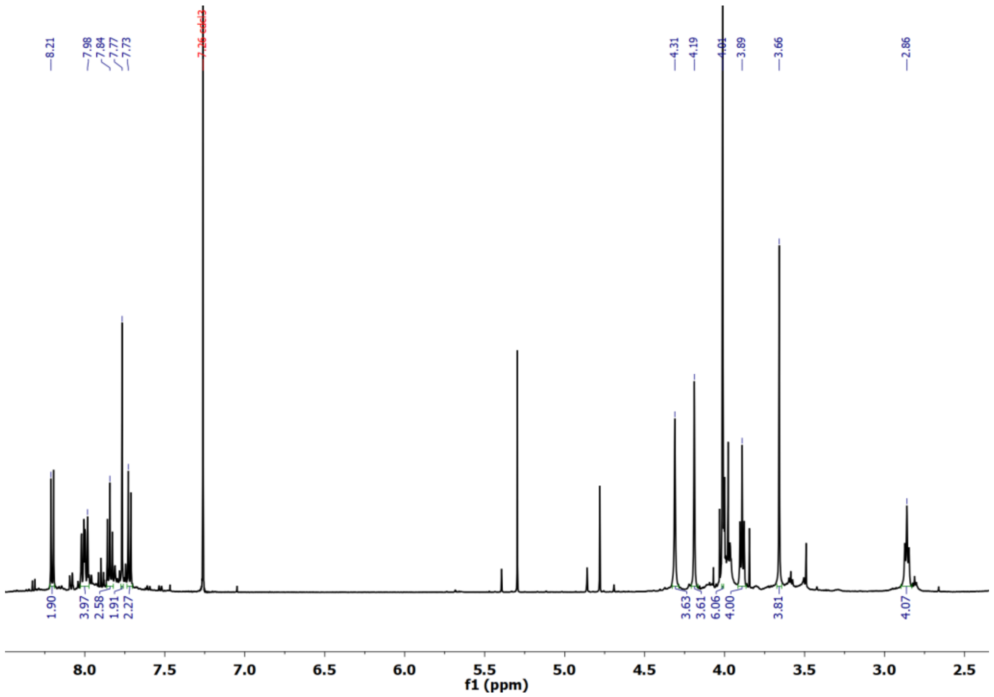

Figure S8. ^{1}H NMR spectrum (500 MHz) of phencrown-*N*,*N′*-dipicolinate methyl ester (**3phen**) in $CDCl_3$.

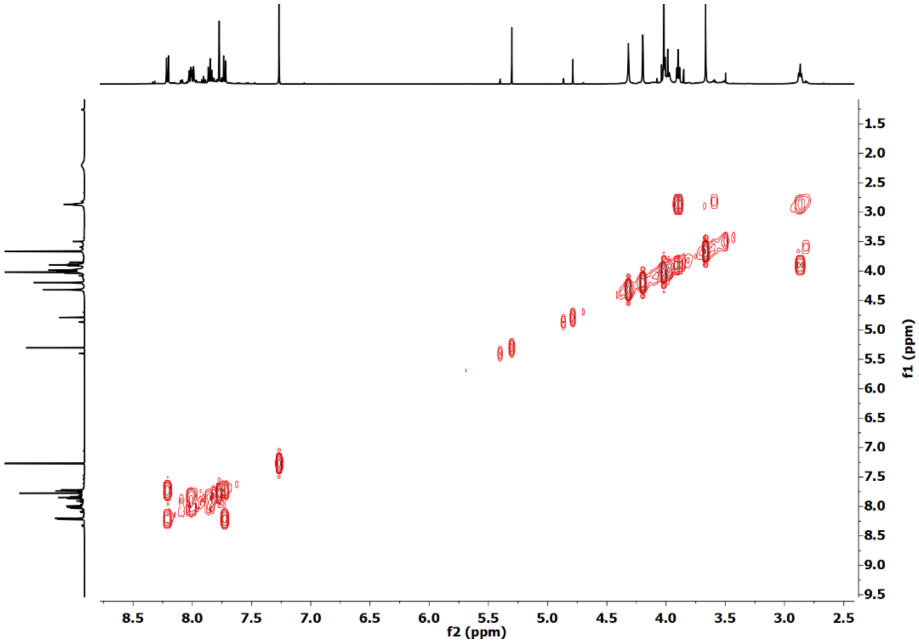

Figure S9. $^{1}H - ^{1}H$ COSY spectrum (500 MHz) of phencrown-*N,N′*-dipicolinate methyl ester (**3phen**) in $CDCl_3$.

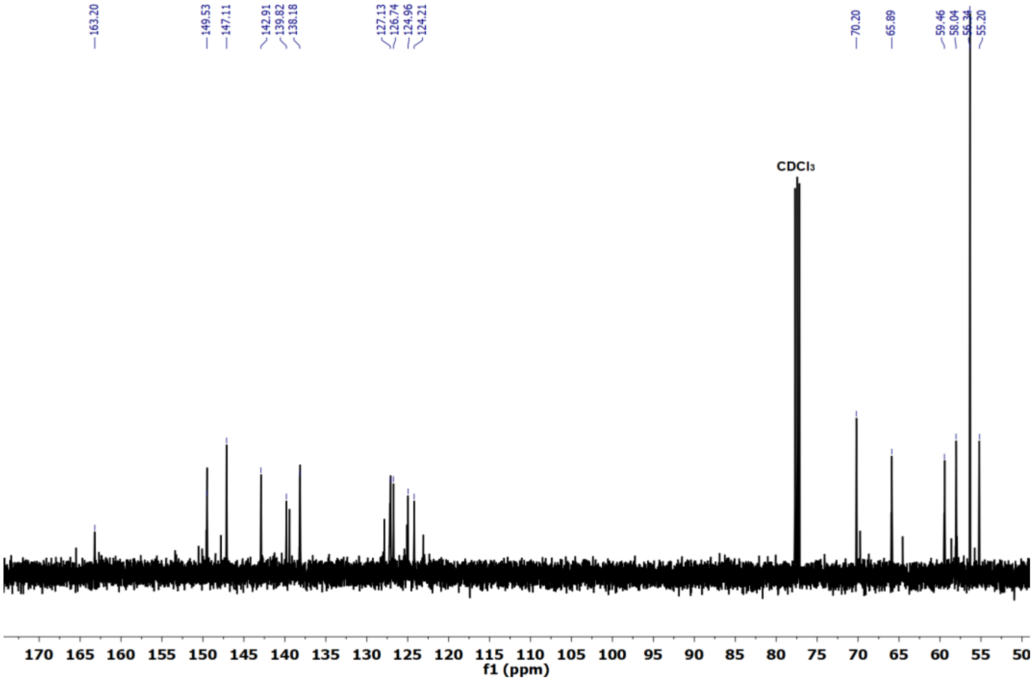

Figure S10. $^{13}C\{^{1}H\}$ NMR spectrum (500 MHz) of phencrown-*N,N′*-dipicolinate methyl ester (**3phen**) in $CDCl_3$.

1.5 Bpycropa

The ^{1}H NMR spectrum of **bpycropa** is consistent with its symmetric structure, showing only half the number of proton resonances expected for the full molecule, *i.e.* chemically equivalent proton pairs are also magnetically equivalent. In the aromatic region, six signals – four doublets and two triplets of equal intensity, each integrating for two protons − are observed. Five additional resonances appear in the aliphatic region, one of which partially overlaps with the solvent peak.

Assignments of the aromatic doublets and triplets were done through a ^{1}H – ^{1}H COSY spectrum (Figure S10), which also revealed long-range couplings between aromatic and aliphatic protons (Figure S11). The resonances of the picolinate protons (H_m, H_l, H_i) were distinguished from those of the bipyridyl unit (H_a, H_b, H_c) by comparing the ^{13}C NMR spectrum of the ligand with that of the relevant building-block analogues (6,6'-dimethyl-2,2'-bipyridine and 6-methyl-pyridin-2-carboxylic acid).**Errore. L'origine riferimento non è stata trovata.** Further confirmation was provided by the ^{1}H – ^{13}C HSQC and HMBC spectra, which display the C–H connectivities (Figures S12a,b) and the correlations to quaternary carbons, including the picolinate carboxylate carbon and the quaternary carbon of the bipyridyl moiety (Figures S13a,b).

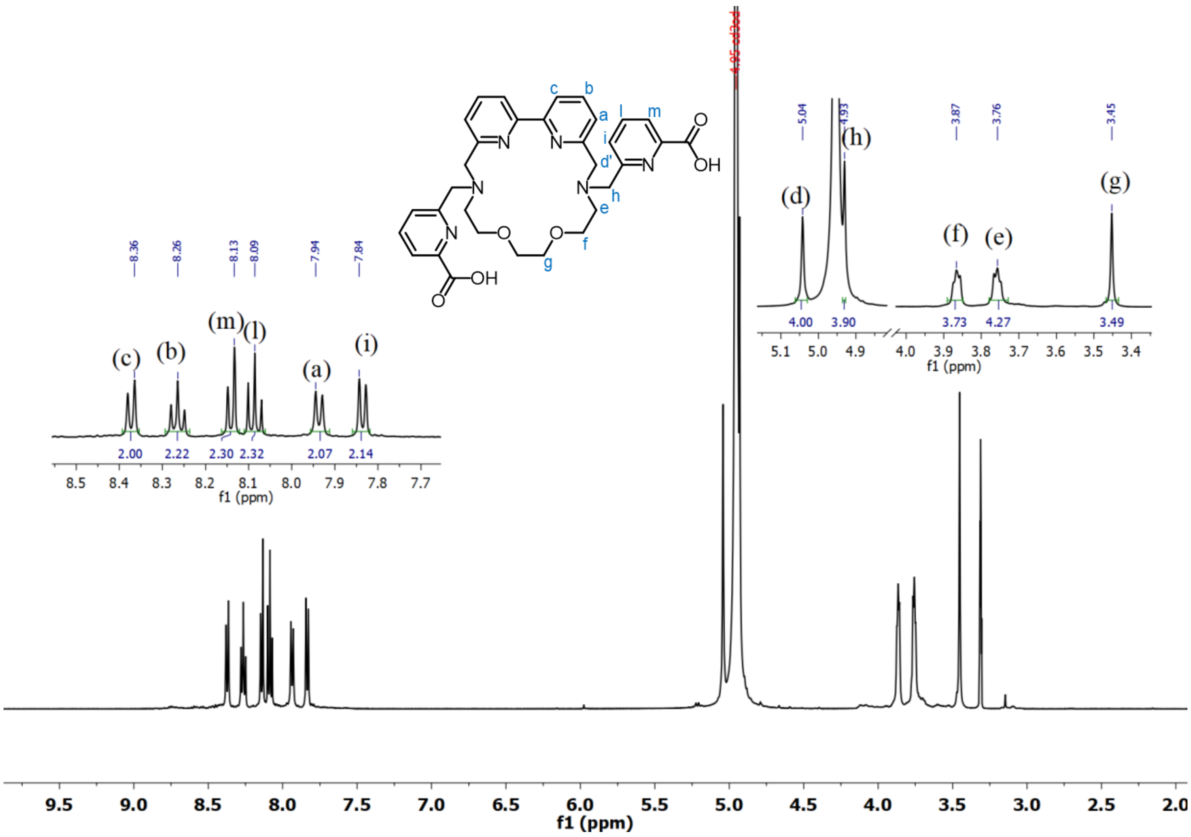

Figure S11. ^{1}H NMR spectrum (500 MHz) of the **bpycropa** ligand in CD_3OD with labeling scheme.

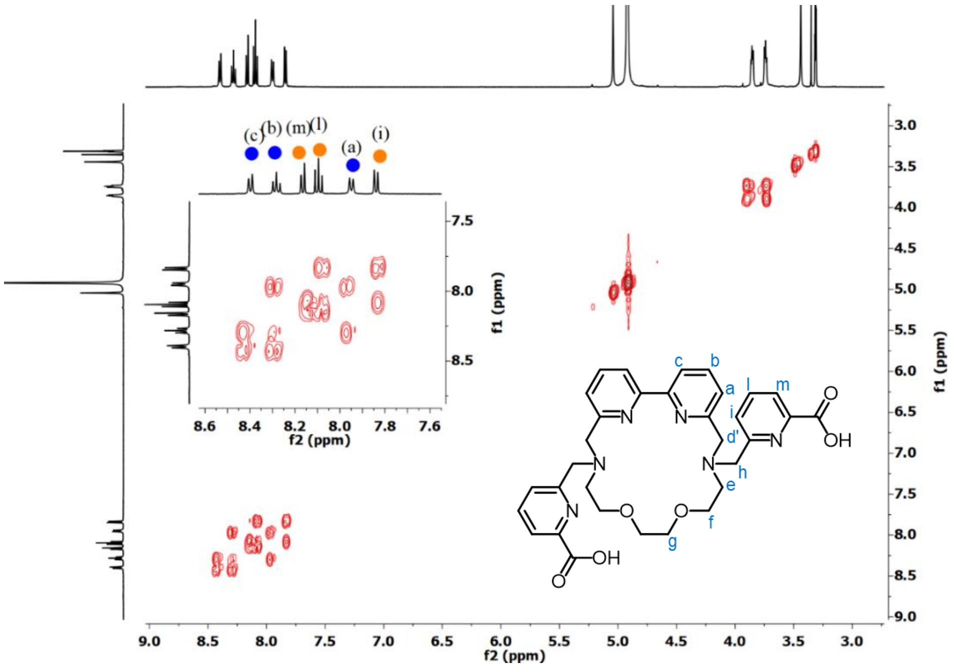

Figure S12. $^1H - ^1H$ COSY spectrum (500 MHz) of the **bpycropa** ligand in CD_3OD with labeling scheme. In the insert, the enlarged aromatic region. Blue dots = resonances of bpy unit, orange dots = resonances of picolinate unit.

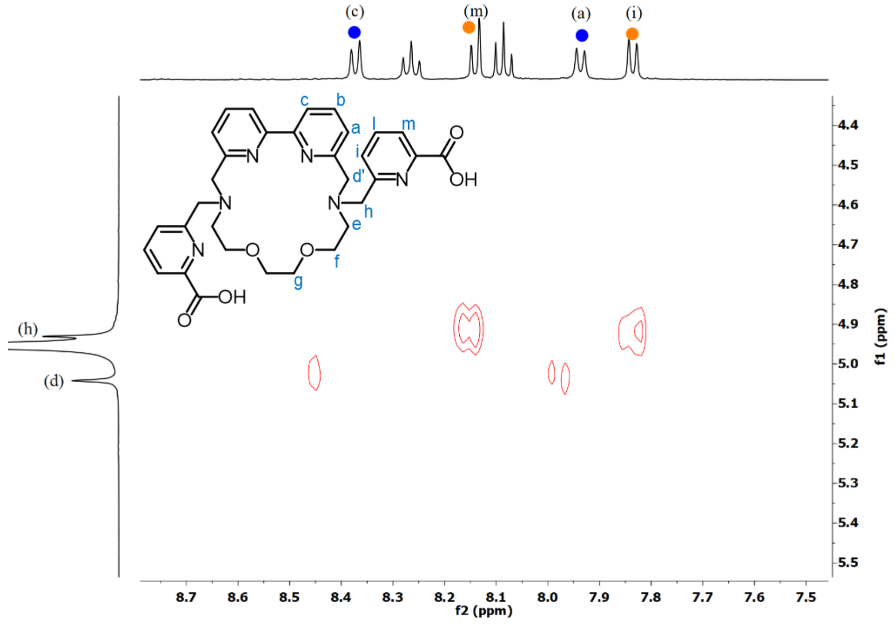

Figure S13. $^1H - ^1H$ COSY spectrum (500 MHz) of **bpycropa** in CD_3OD highlighting long-range coupling between the resonances of aromatic (H_c, H_m, H_a, H_i) and aliphatic (H_d, H_h) protons. Blue dots = resonances of bpy unit, orange dots = resonances of picolinate unit.

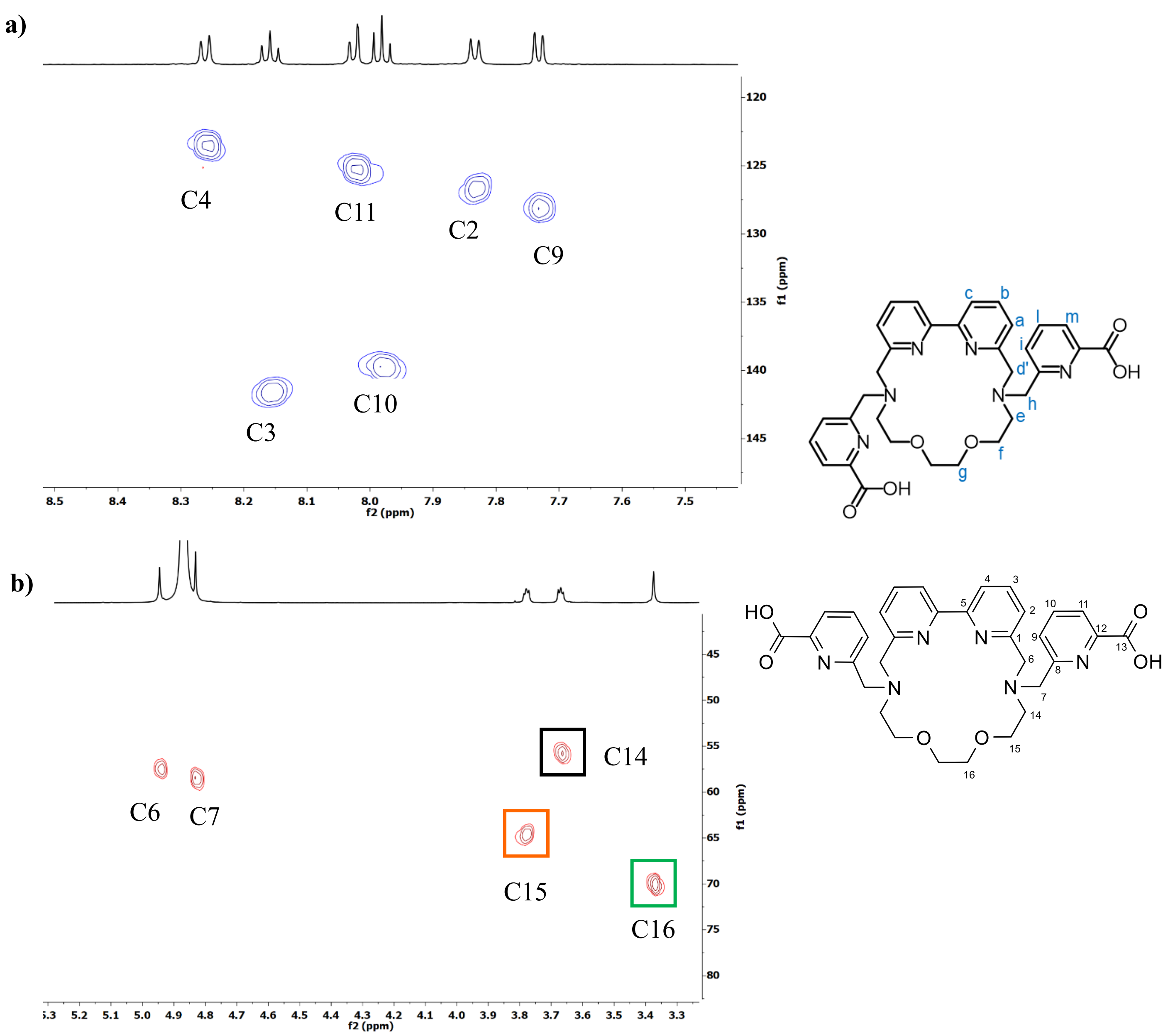

Figure S14. Enlarged view of the aromatic (**a**) and aliphatic (**b**) region of the ^{1}H – ^{13}C HSQC spectrum (500 MHz) of **bpycropa**.

In the deshielded aliphatic region, two singlets are observed at 5.03 ppm (H_d) and 4.73 ppm (H_h) that in the ^{1}H – ^{13}C HSQC spectrum have cross peaks with C6 and C7, respectively (Figure S12b). In addition to the long-range couplings evident in the ^{1}H – ^{1}H COSY spectrum, the distinction between H_d and H_h was achieved through their correlations in the ^{1}H – ^{13}C HMBC spectrum. The H_h protons correlate with carbons C8 and C9 at 150.11 and 127.52 ppm, respectively − carbons that also show correlations with protons H_i and H_l (Figure S13a, red frames). Conversely, the H_d protons correlate with carbons C1 and C2 at 149.20 and 125.91 ppm, which in turn correlate with the bipyridyl protons H_c and H_b (Figure S13a, blue frames).

This assignment was further supported by cross peaks between the picolinate protons H_l and H_m and the deshielded carboxylate carbon at 164.9 ppm in the ^{1}H – ^{13}C HMBC spectrum (Figure S13b). The chemical shift of this carboxylate carbon matches that observed in the ^{13}C NMR spectrum of the 6-methyl-pyridin-2-carboxylic acid fragment, which served as an additional reference.

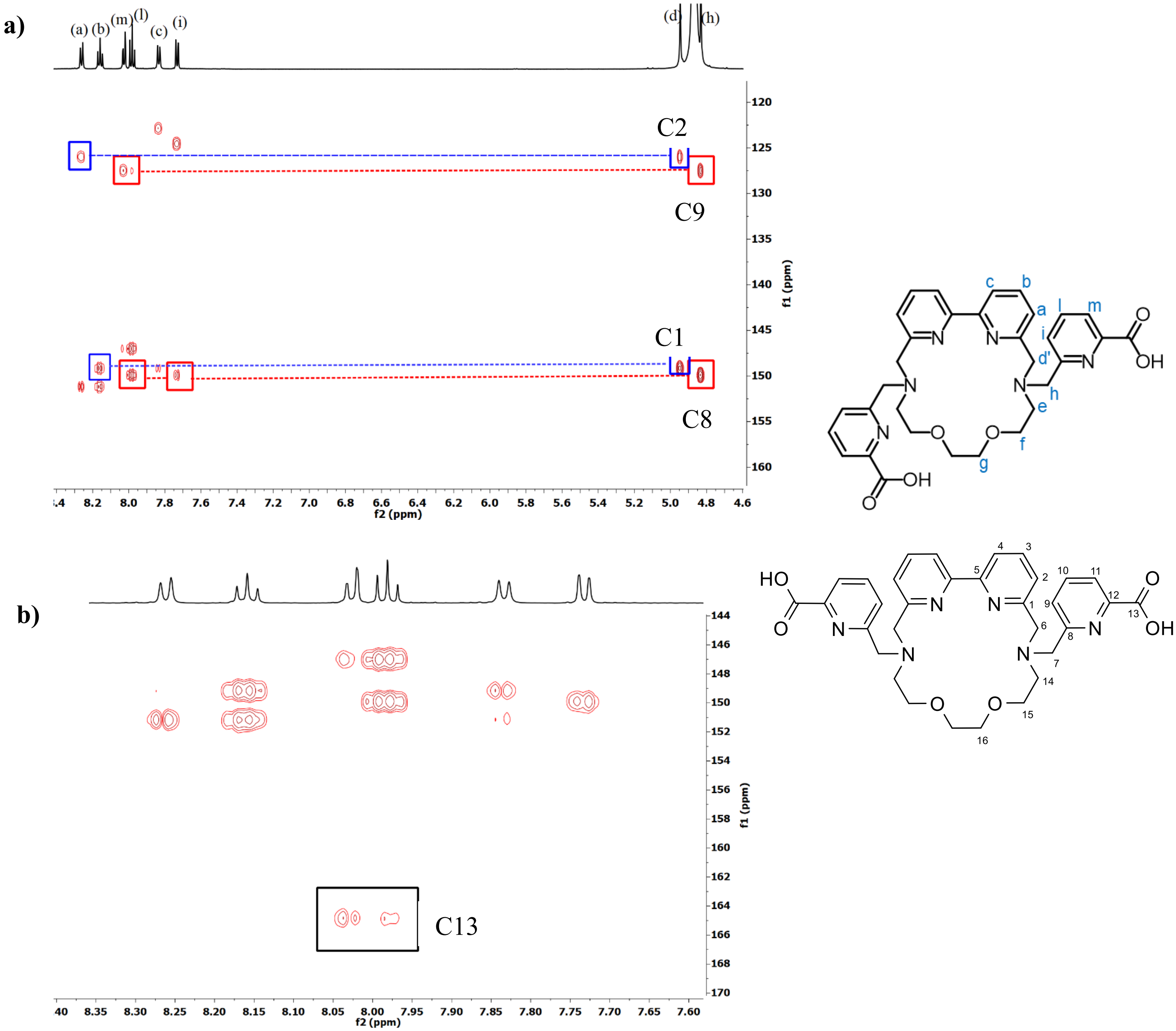

Figure S15. Enlarged sections of the ^{1}H – ^{13}C HMBC spectrum of **bpycropa**. In panel **a**, correlations between aromatic carbons C8, C9 and the protons H_i, H_l and H_h (red frames) are visible, along with correlations between aromatic carbons C1, C2 and H_b, H_c and H_d protons (blue frames). Panel **b** displays the correlations between the aromatic protons H_m, H_l and the carboxylate carbon C13 of the picolinate group.

1.6 Phencropa

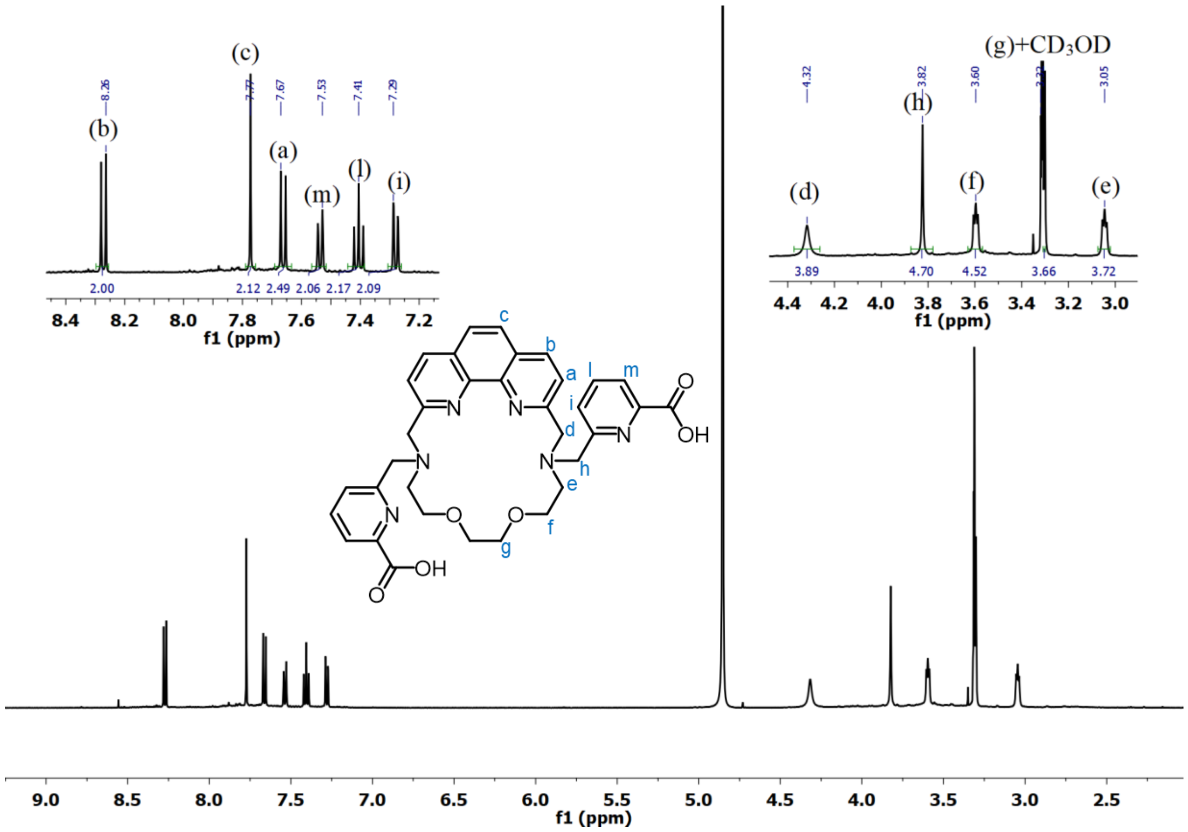

Figure S16. ^{1}H NMR spectrum (500 MHz) of **phencropa** in CD_3OD with labeling scheme.

The spectrum of **phencropa** is similar to that of **bpycropa** (Figure S14): also in this case the number of resonances corresponds to half the total number of protons present in the molecule, in agreement with its symmetry (*i.e.* chemically equivalent protons are also magnetically equivalent). In total, six resolved resonances of equal intensity (2H) in the range 7.2 – 8.3 ppm can be observed. The singlet at 7.79 ppm is unequivocally assigned to the H_c protons of the phenanthroline moiety. The remaining multiplets were readily attributed either to phenanthroline − doublets H_b and H_a at 8.29 ppm and 7.69 ppm, respectively − or to the picolinate rings – H_l, H_m and H_i via a ^{1}H – ^{1}H COSY spectrum (Figure S15). The doublet at highest frequency is assigned to the two H_b protons positioned *para* to the nitrogen atom of the phenanthroline ring, which are deshielded due to resonance effects.

In the aliphatic region, four of the five expected resonances (4H each) are observed. The two triplets correspond to the methylene groups of the macrocycle, H_e and H_f. The assignment of these resonances was performed through the ^{1}H – ^{13}C HSQC spectrum (Figures S16 and S17), as for **bpycropa**. In particular, carbons C15 and C16 that resonate at 54.97 and 65.03 ppm, are bound to protons H_e and H_f, respectively (Figure S17). Further confirmation of the H_e and H_f assignments came from the pH dependence of their chemical shifts (*vide infra*, ^{1}H NMR titration studies). In

fact, the resonance of H_e, located closer to the tertiary nitrogen, exhibited a larger chemical shift change upon protonation of the amino group compared to H_f.

Lastly, by analyzing the long-range couplings between aliphatic and aromatic protons of the ^1H – ^1H COSY spectrum (not shown in Figure S15), it was possible to assign the broadened singlet at 4.33 ppm, which displays a cross peak with the resonance of H_a, to the H_d protons, and the slightly broadened singlet at 3.84 ppm to the H_h protons due to long-range coupling with the resonance of H_i. The signal of the four H_g protons overlaps with the quintet of the solvent CD_3OD.

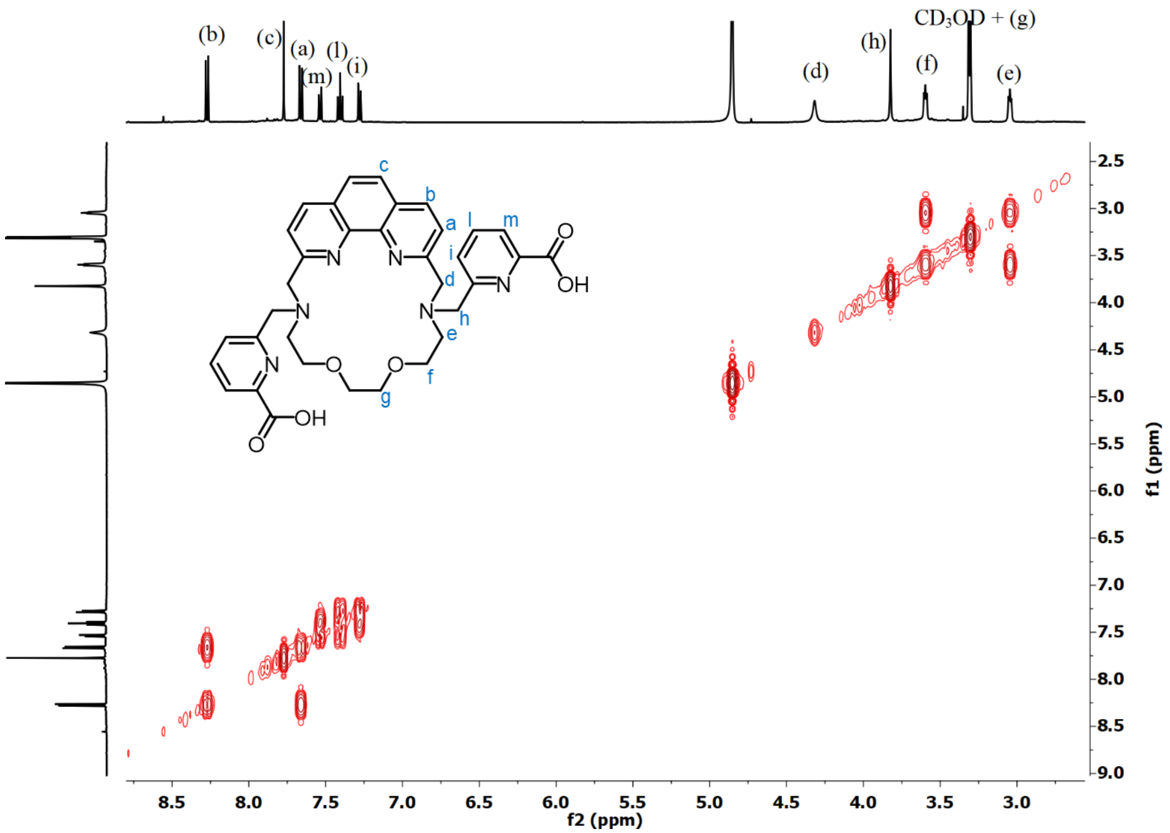

Figure S17. ^1H – ^1H COSY spectrum (500 MHz) of **phencropa** in CD_3OD.

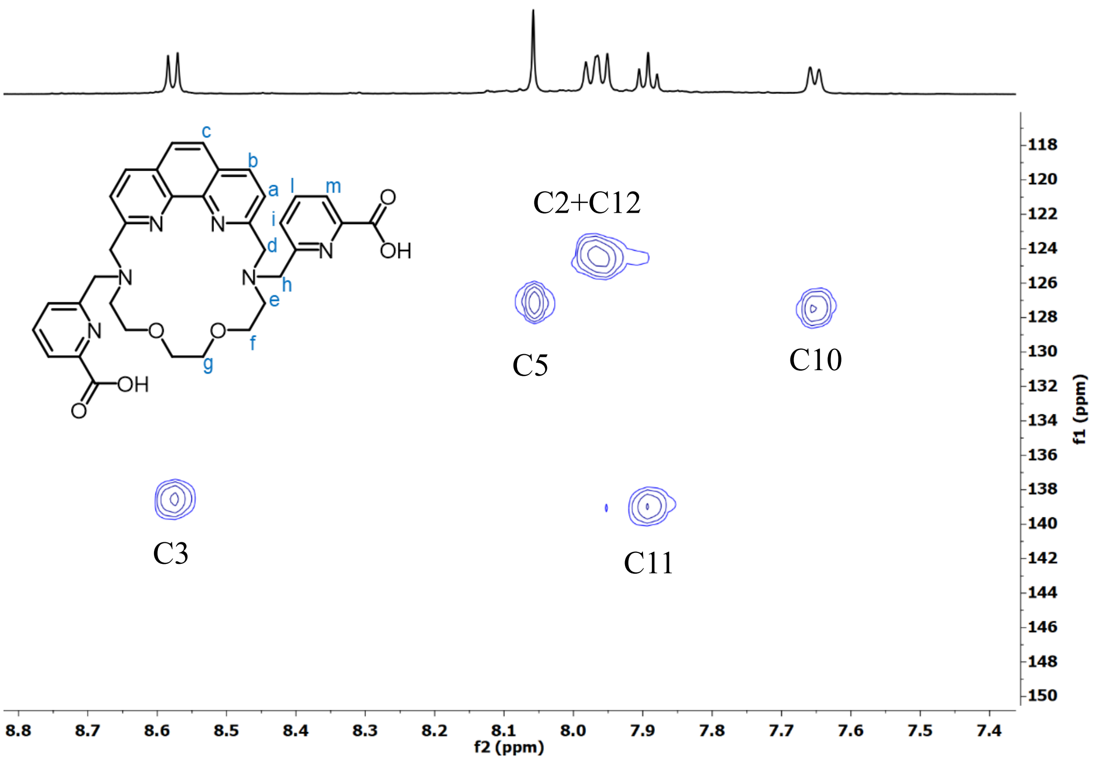

Figure S18. $^{1}H - ^{13}C$ HSQC spectrum (500 MHz) of the aromatic region of **phencropa** in CD_3OD.

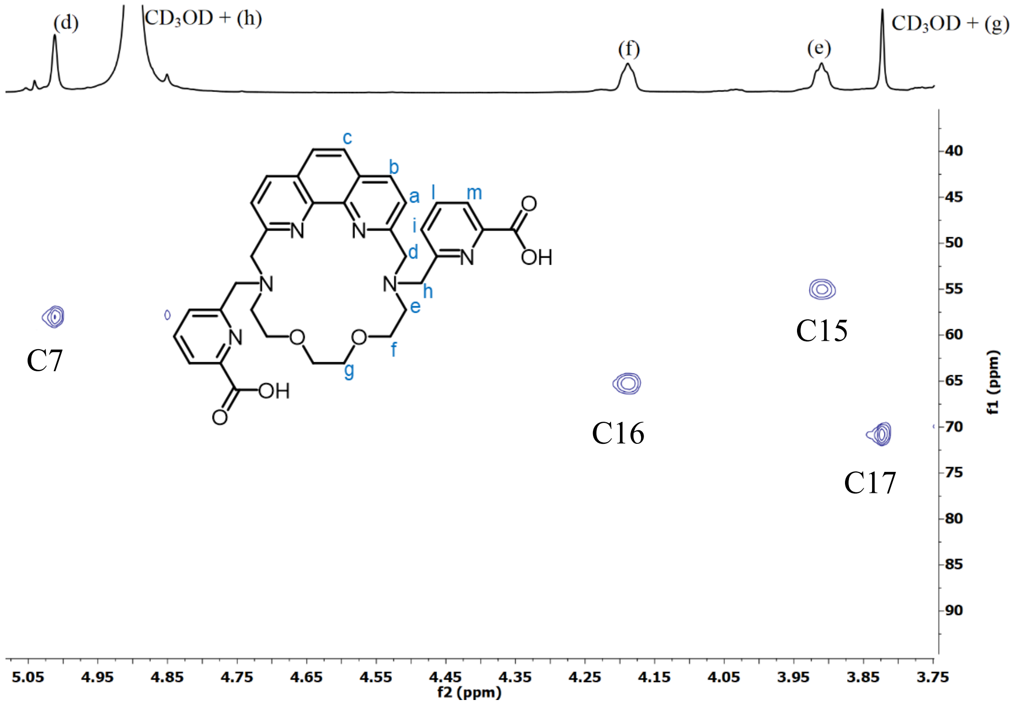

Figure S19. $^{1}H - ^{13}C$ HSQC spectrum (500 MHz) of the aliphatic region of **phencropa** in CD_3OD.

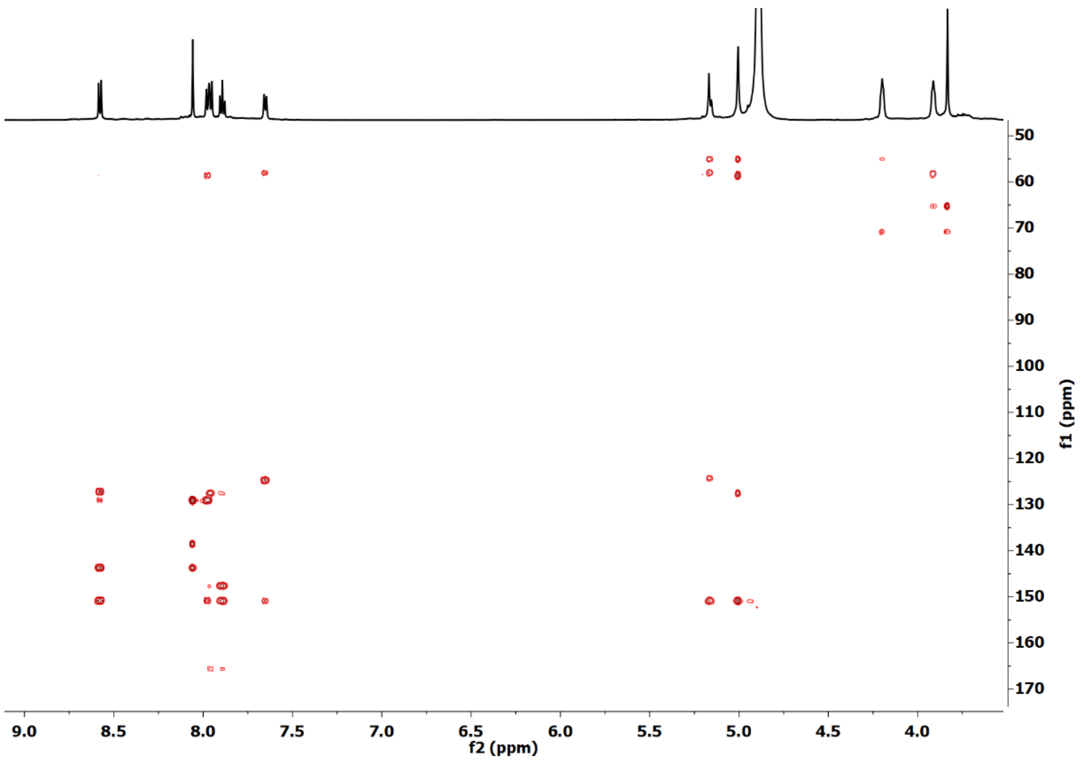

Figure S20. ^{1}H – ^{13}C HMBC spectrum (600 MHz) of **phencropa** in CD_3OD.

1.7 [Ba(bpycropa)] (4)

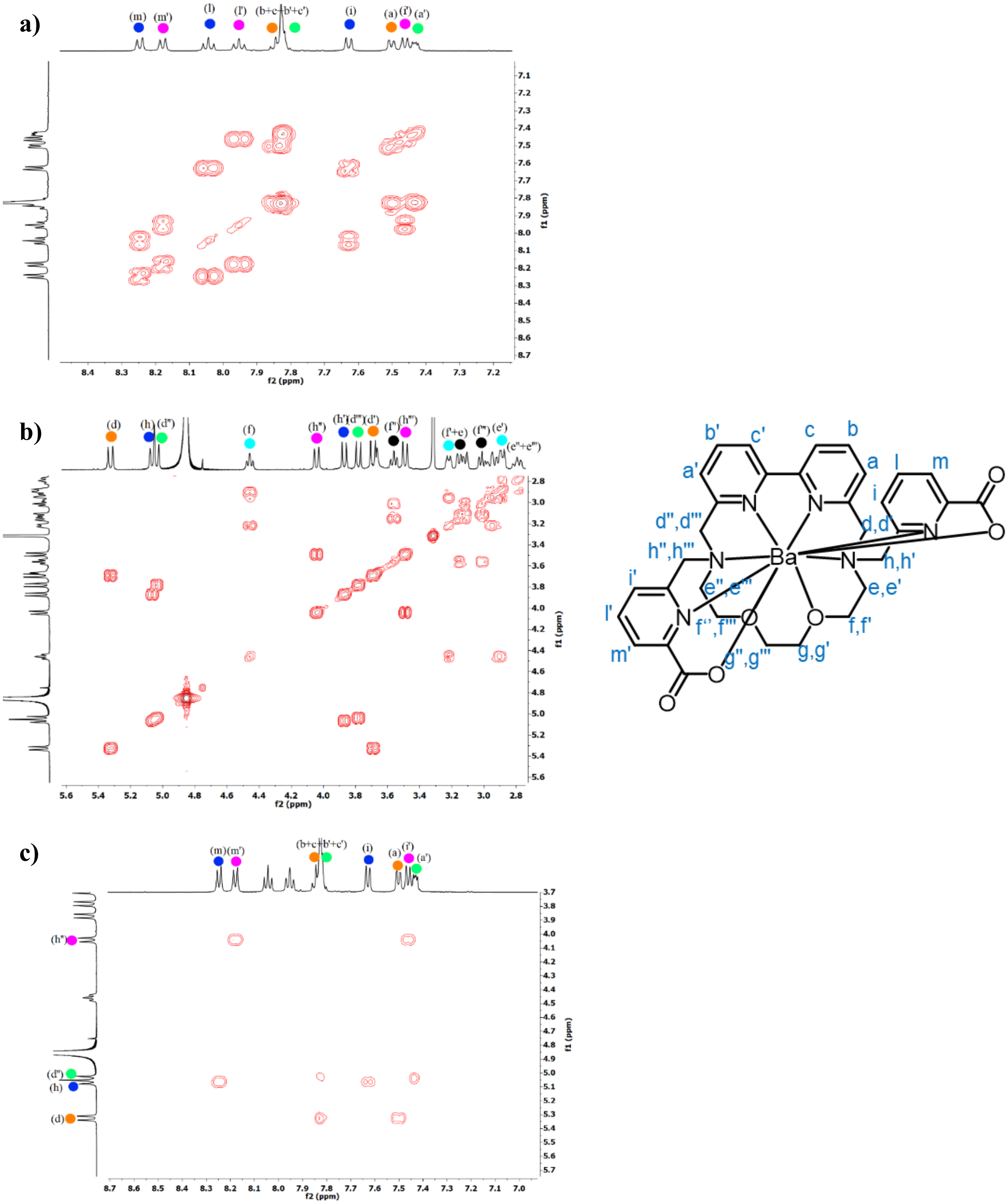

Figure S21. ^1H – ^1H COSY spectrum (500 MHz) of complex (**4**) in CD_3OD highlighting the aromatic (**a**), aliphatic (**b**), and long-range aromatic-aliphatic couplings (**c**). Color coding identifies couplings between proton pairs with at least one resolved resonance. The 2D drawing with the

labeling scheme is not representative of the true geometry of the complex, which can be seen in Figure 3 of the main text.

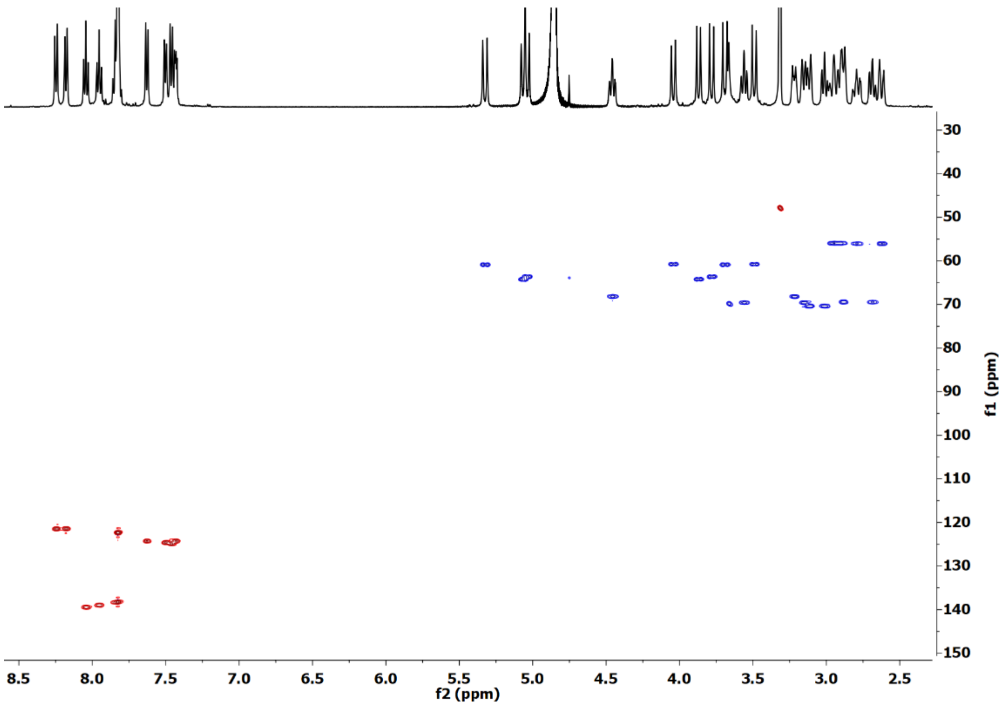

Figure S22. ^{1}H – ^{13}C HSQC spectrum (500 MHz) of [Ba(**bpycropa**)] (**4**) in CD_3OD.

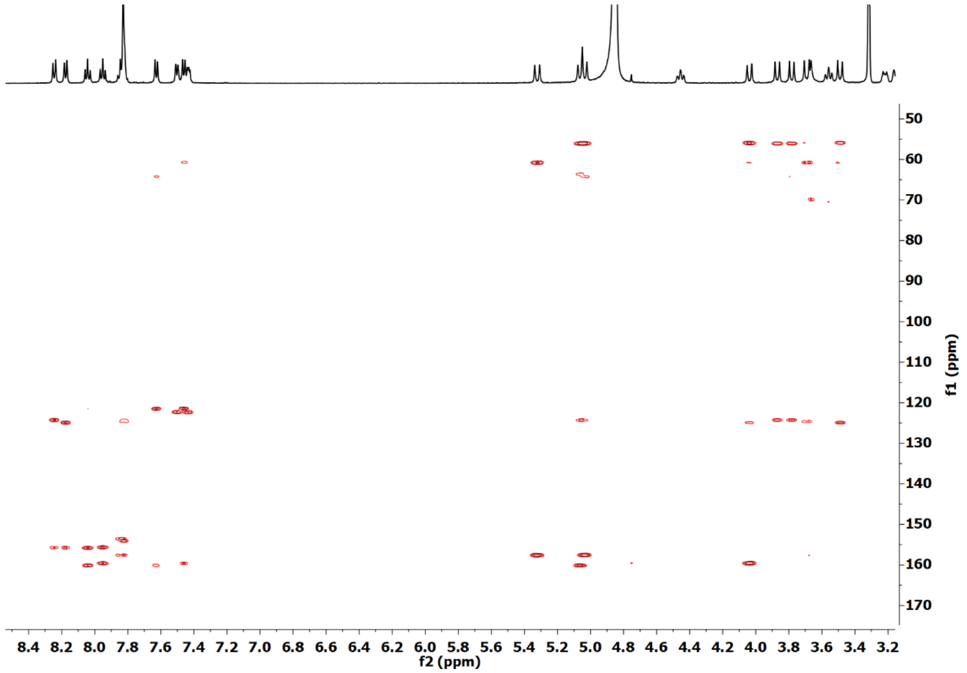

Figure S23. ^{1}H – ^{13}C HMBC spectrum (500 MHz) of [Ba(**bpycropa**)] (**4**) in CD_3OD.

1.8 [Ba(phencropa)] (5)

As for the [Ba(**bpycropa**)] (**4**) complex, the ^{1}H NMR spectrum of [Ba(**phencropa**)] (**5**) also shows a number of resonances significantly larger compared to the free ligand. Sixteen well-resolved resonances are observed (five in the aromatic region and eleven in the aliphatic region, each integrating for 1H). The remaining 16 protons give resonances that partially overlap in four additional multiplets. Thus, in this case as well, all protons are magnetically inequivalent and resonate at different frequencies due to the loss of ligand symmetry upon coordination. All resonances were assigned through a ^{1}H – ^{1}H COSY spectrum (Figures S23a and S23b), followed by analysis of ^{1}H – ^{13}C HSQC and ^{1}H – ^{13}C HMBC spectra (Figures S24 and S25), as done for [Ba(**bpycropa**)] (**4**).

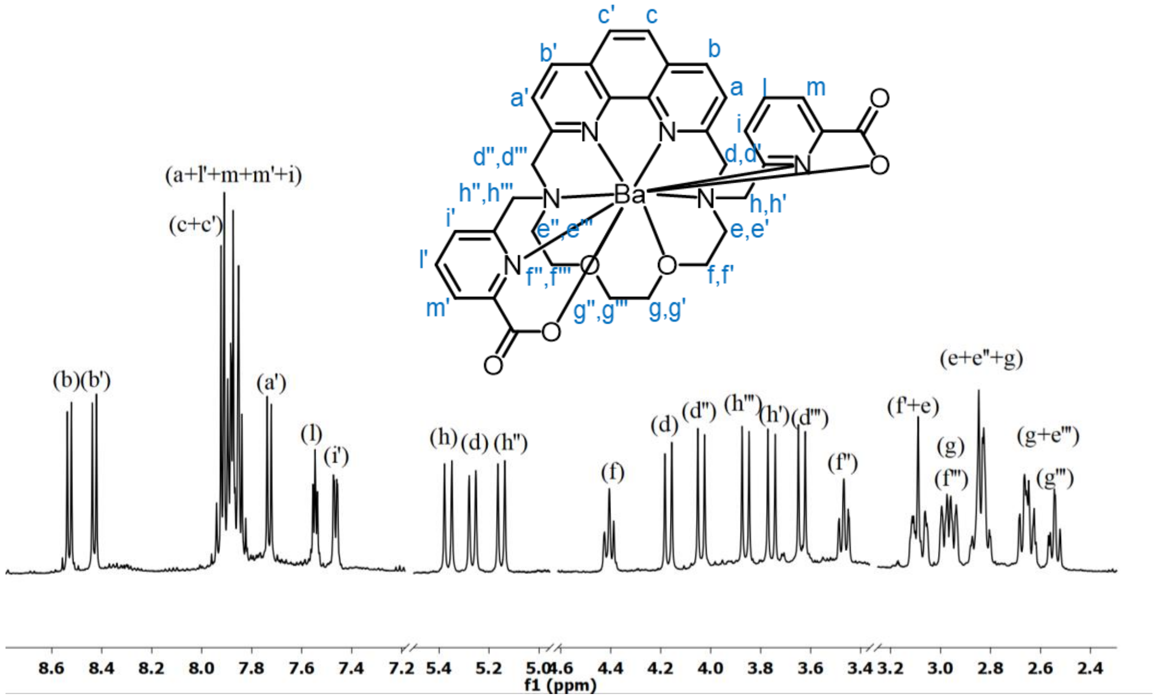

Figure S24. ^{1}H NMR spectrum (500 MHz) of [Ba(**phencropa**)] (**5**) in CD_3OD with labeling scheme. The 2D drawing with the labeling scheme is not representative of the true geometry of the complex, which can be seen in Figure 3 of the main text.

Furthermore, as done for (**4**), the ^{1}H – ^{1}H ROESY spectrum of complex **5** shows exchange cross peaks between the resonances of the chemically equivalent proton pairs, *e.g* the pairs of phenanthroline protons $H_b/H_{b'}$ and $H_a/H_{a'}$, as well as the pairs of picolinate protons $H_l/H_{l'}$ and $H_i/H_{i'}$ (Figure S26).

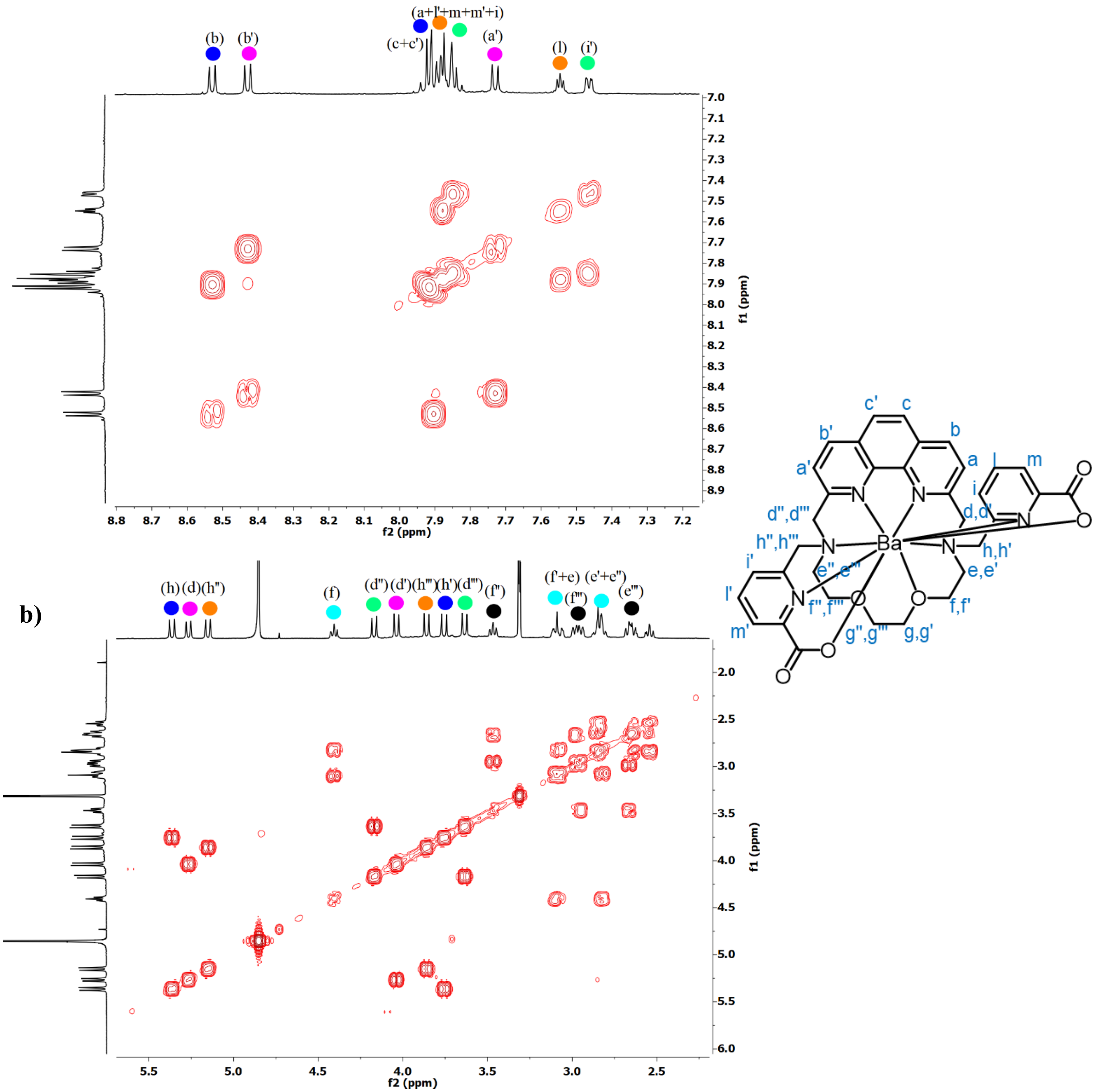

Figure S25. $^{1}H - ^{1}H$ COSY spectrum (500 MHz) of [Ba(**phencropa**)] (**5**) in CD_3OD with labeling scheme: (**a**) aromatic region, (**b**) aliphatic region. Color coding identifies couplings between proton pairs with at least one resolved resonance. The 2D drawing with the labeling scheme is not representative of the true geometry of the complex, which can be seen in Figure 3 of the main text.

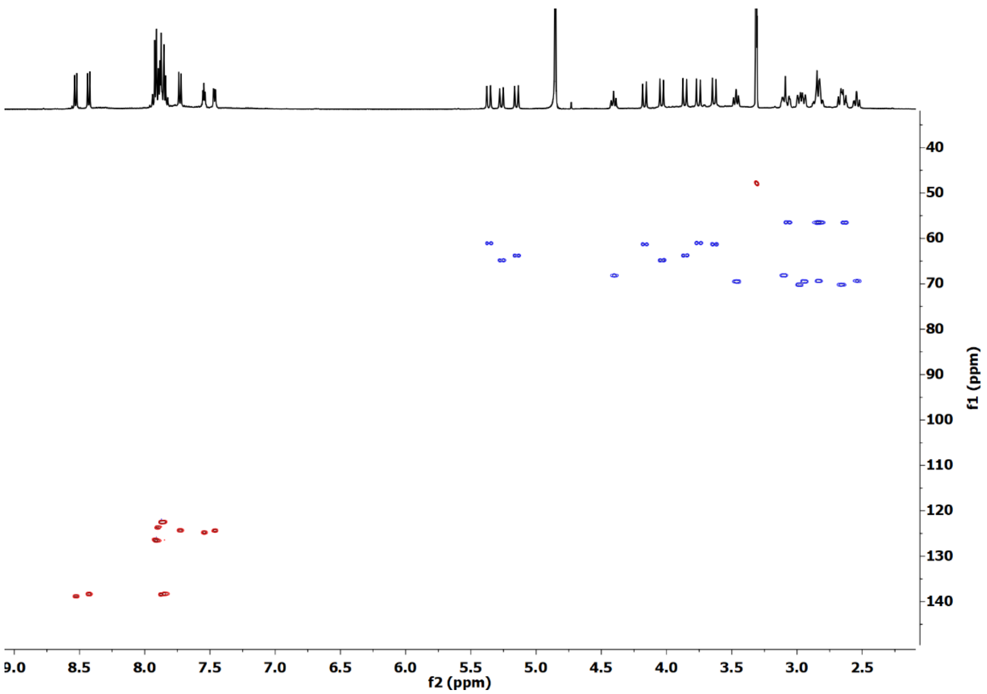

Figure S26. ^{1}H – ^{13}C HSQC spectrum (600 MHz) of [Ba(**phencropa**)] (**5**) in CD_3OD.

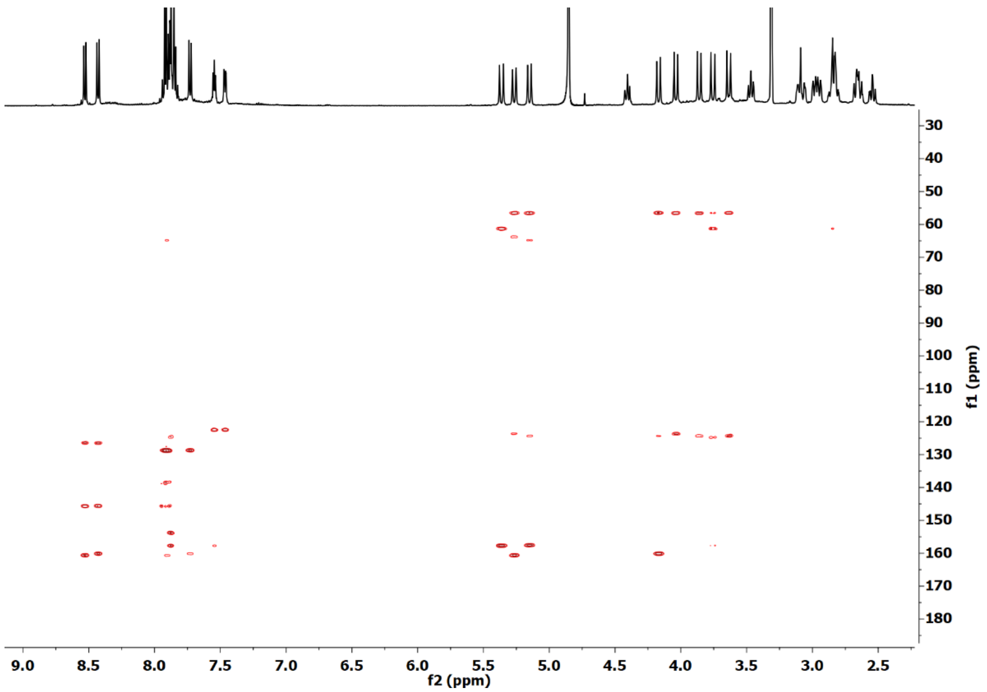

Figure S27. ^{1}H – ^{13}C HMBC spectrum (600 MHz) of [Ba(**phencropa**)] (**5**) in CD_3OD.

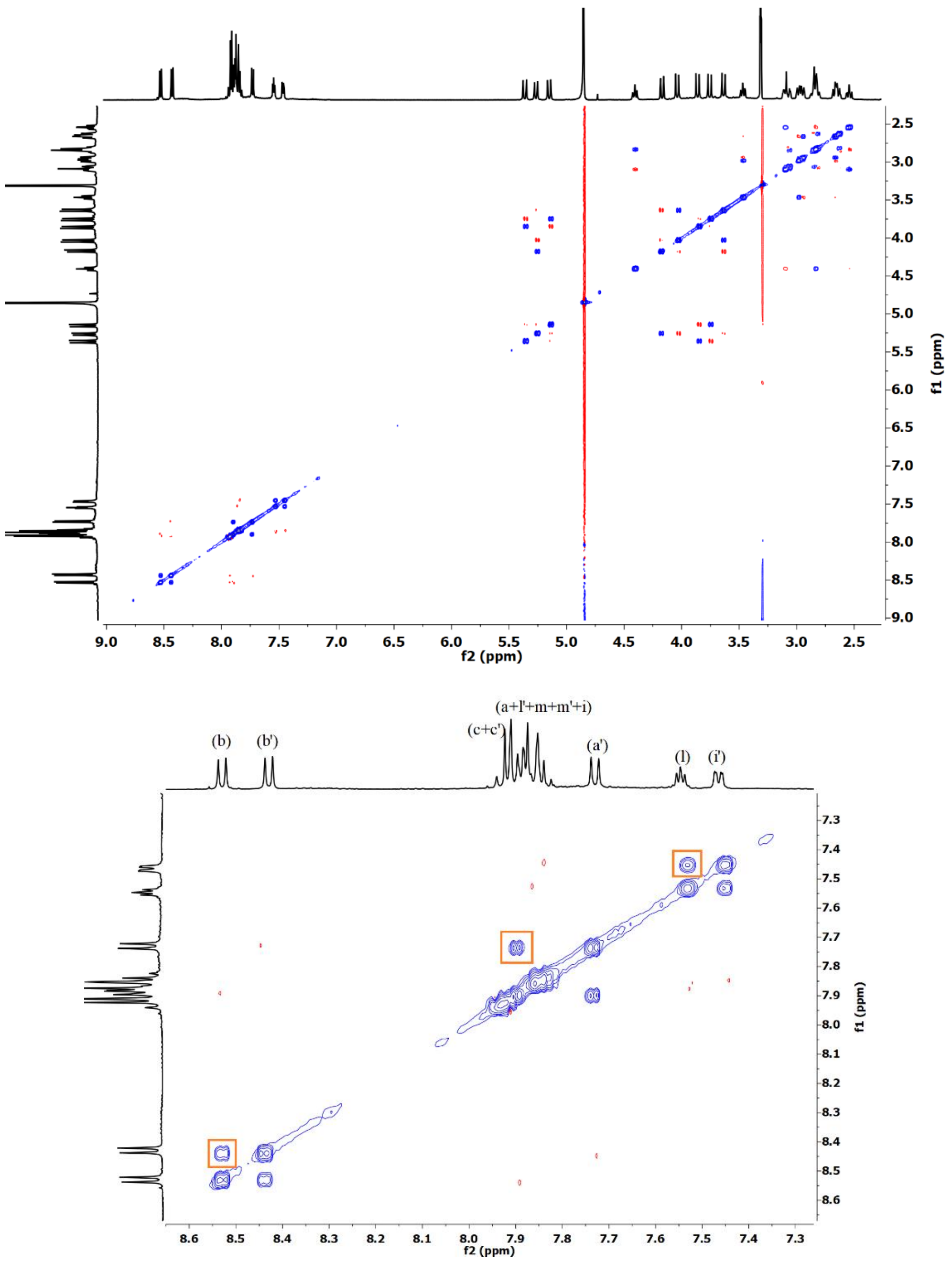

Figure S28. $^1H - ^1H$ ROESY spectrum (600 MHz) of [Ba(**phencropa**)] (**5**) in CD_3OD (mixing time = 300 ms): full spectrum (top) and expansion of the aromatic region (bottom). Exchange cross-peaks are highlighted by orange frames.

2. Thermodynamic studies: ^{1}H NMR titrations of bpycropa and phencropa

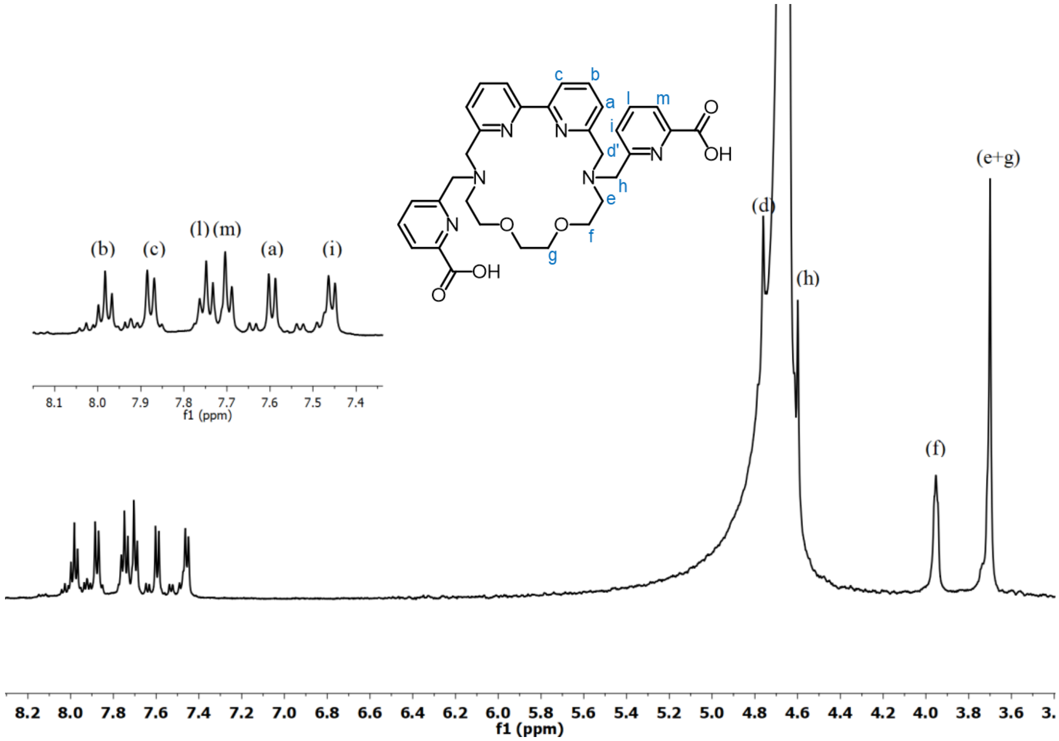

Figure S29. ^{1}H NMR spectrum (500 MHz) of the **bpycropa** ligand in D_2O at pH = 1.38 with labeling scheme.

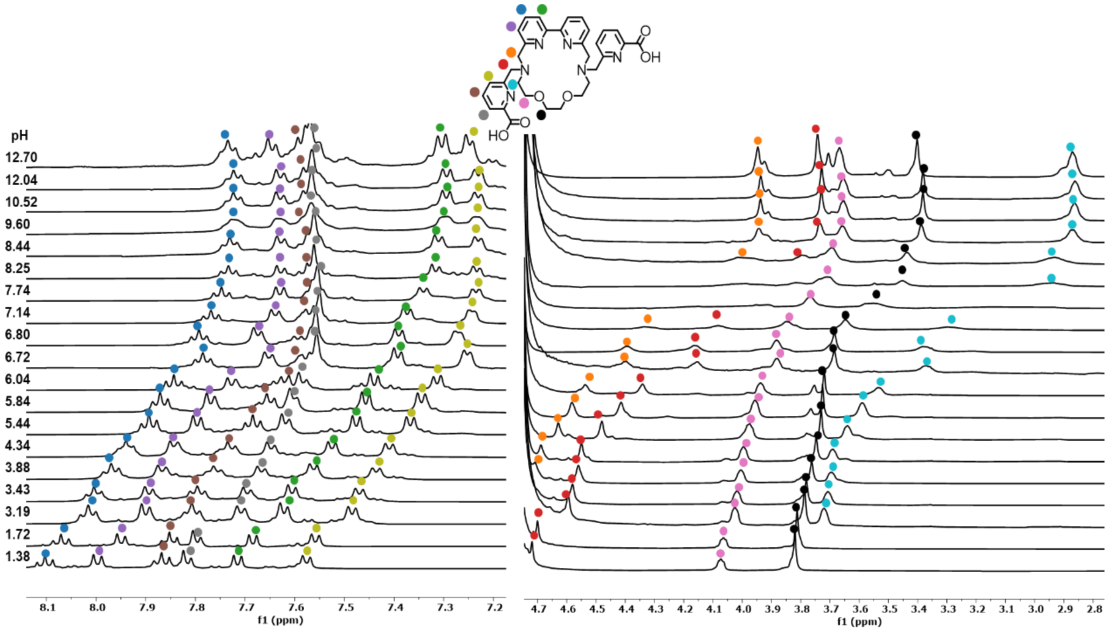

Figure S30. ^{1}H NMR titration of the **bpycropa** ligand in D_2O. See Figure S27 for the labeling scheme. Color coding identifies and tracks the protons of the system as a function of pH.

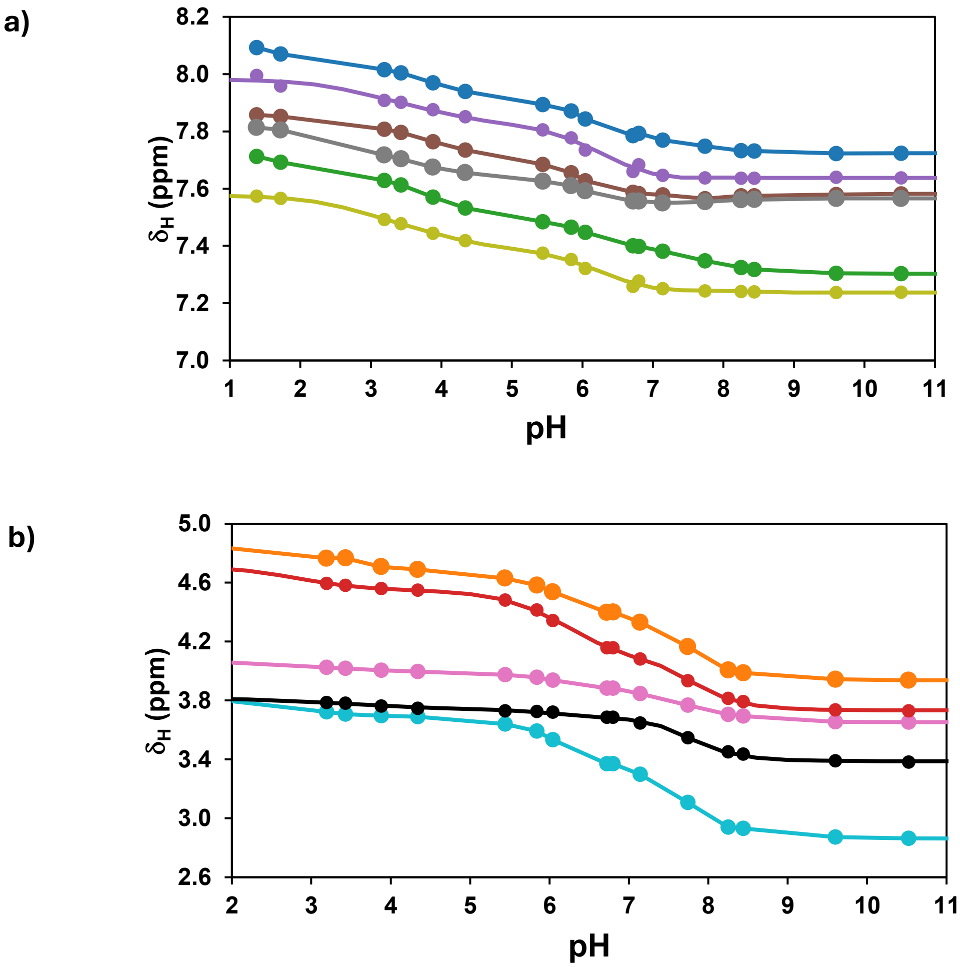

Figure S31. Fitted chemical shifts for the resonances of **bpycropa** relative to the aromatic protons (**a**) and aliphatic protons (**b**) *vs* pH.

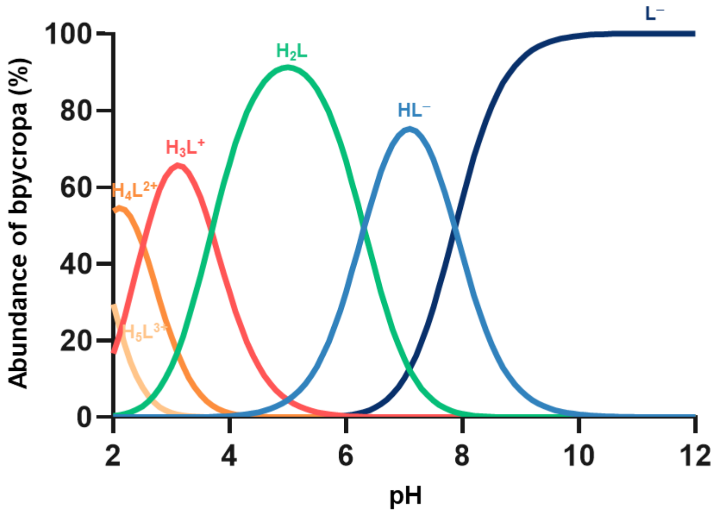

Figure S32. Speciation diagram for the **bpycropa** ligand (here labelled as **H2L**) as a function of pH. [**bpycropa**] = 0.002 M, [$NaNO_3$] = 0.15 M.

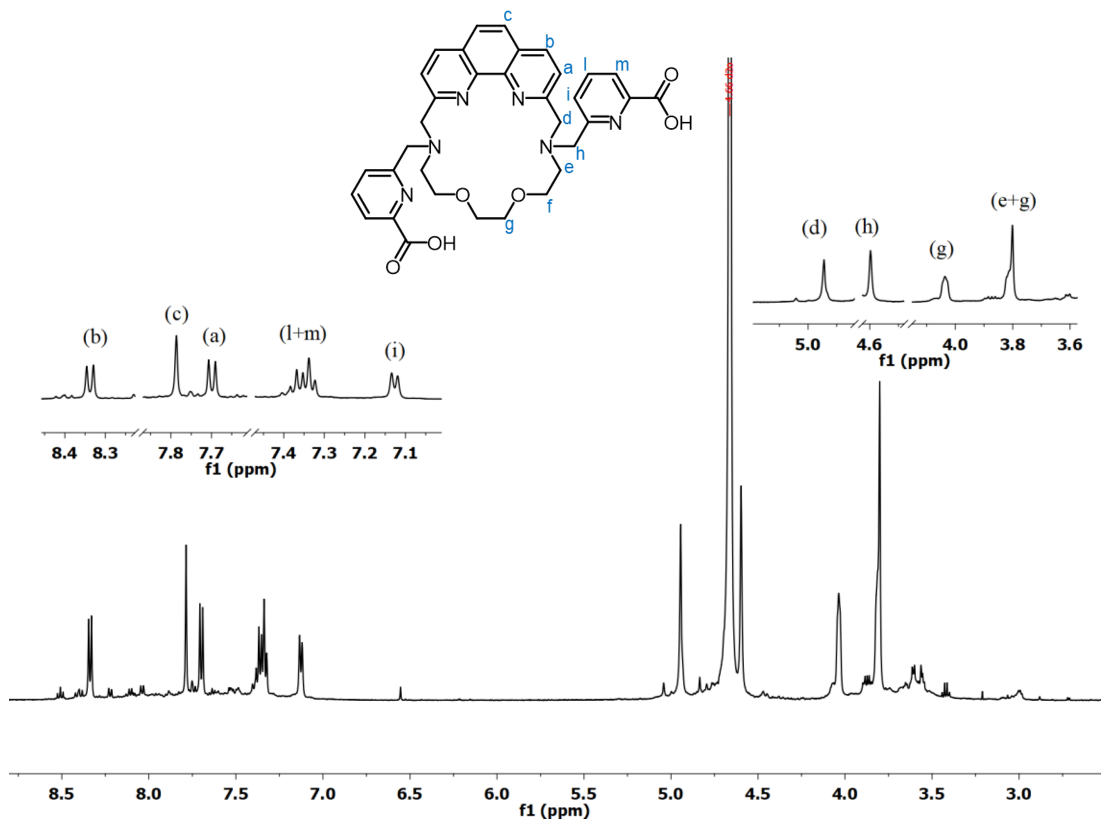

Figure S33. ^{1}H NMR spectrum (500 MHz) of **phencropa** in D_2O at pH = 1.69 with labeling scheme.

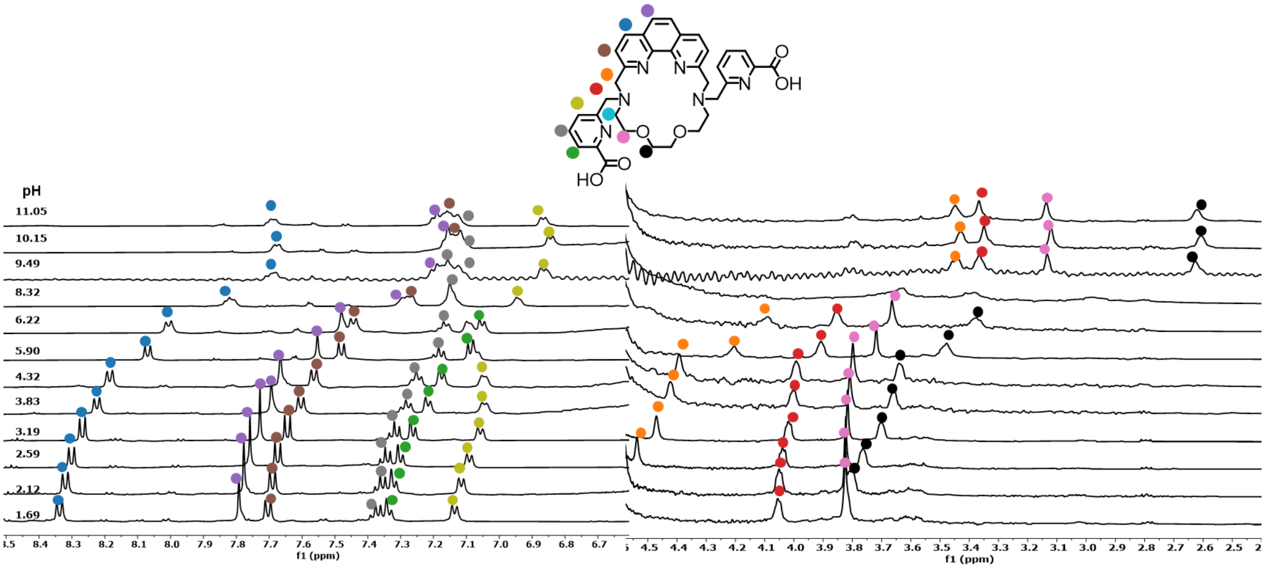

Figure S34. ^{1}H NMR titration of the **phencropa** ligand in D_2O. See Figure S31 for the labeling scheme. Color coding identifies and tracks the protons of the system as a function of pH.

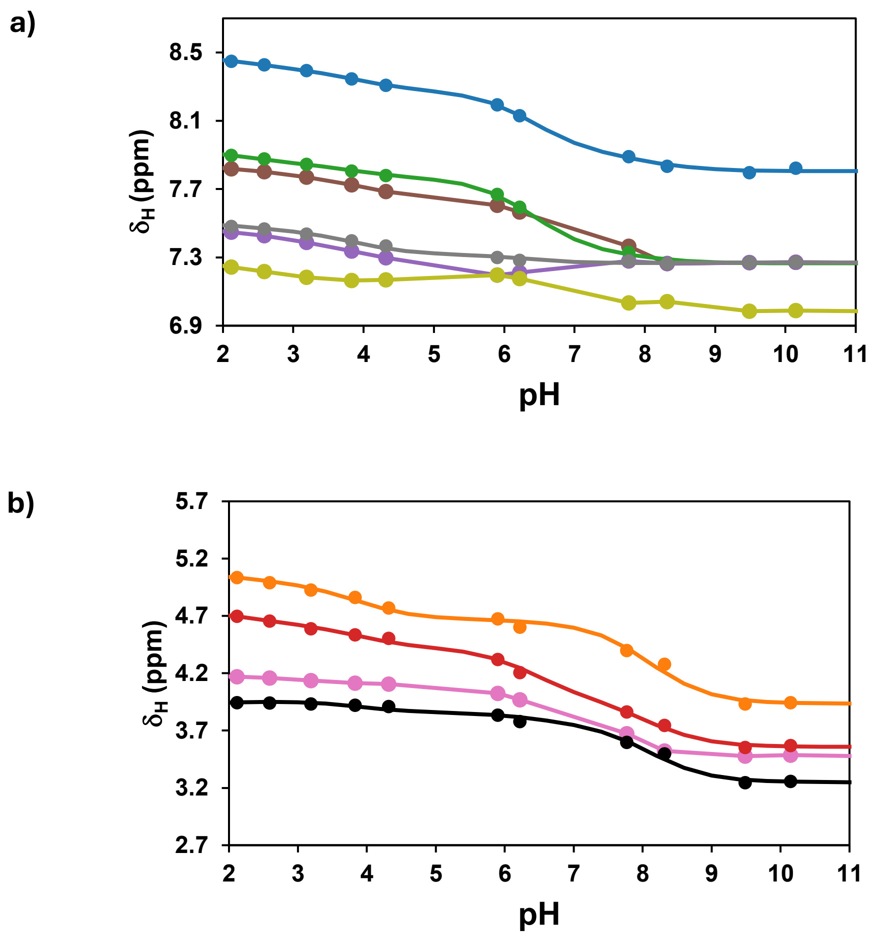

Figure S35. Fitted chemical shifts for the resonances of **phencropa** relative to the aromatic protons (**a**) and aliphatic protons (**b**) *vs* pH.

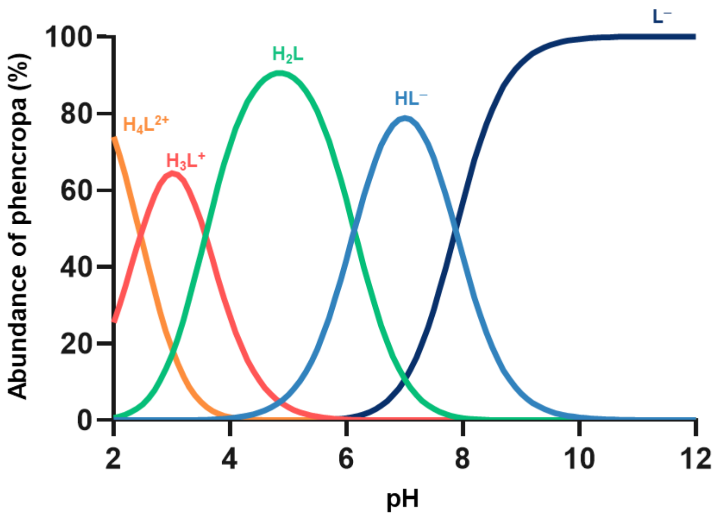

Figure S36. Speciation diagram for the **phencropa** ligand (here labelled as $\mathbf{H_2L}$) as a function of pH. [**phencropa**] = 0.002 M, [$NaNO_3$] = 0.15 M.

3. Kinetic inertness studies

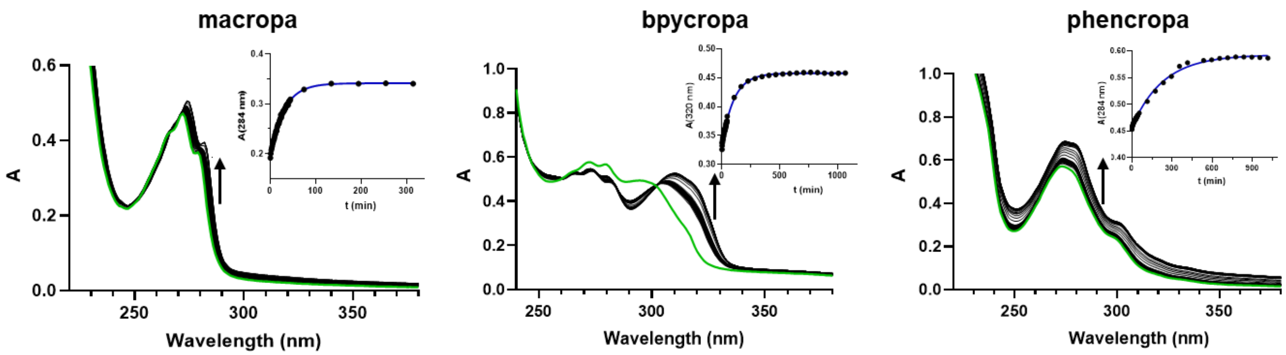

Figure S37. UV spectra of La(III)-mediated transmetalation reactions of [Ba(**macropa**)] (left), [Ba(**bpycropa**)] (middle), [Ba(**phencropa**)] (right) with a 100-fold excess of $LaCl_3$ at pH = 7.4, T = 20 °C, I = 0.15 M NaCl. The green curves represent the spectra of the complexes before the addition of La(III); the black curves correspond to the spectra recorded every 1 minute for the first 45 minutes and every 60 minutes thereafter, after adding La(III). The curves in the inserts were fitted with Eq. 3 and correspond to the change in absorbance vs time monitored at λ = 284 nm for [Ba(macropa)] and [Ba(phencropa)], and at λ = 320 nm for [Ba(bpycropa)]

4. Radium-223 labelling

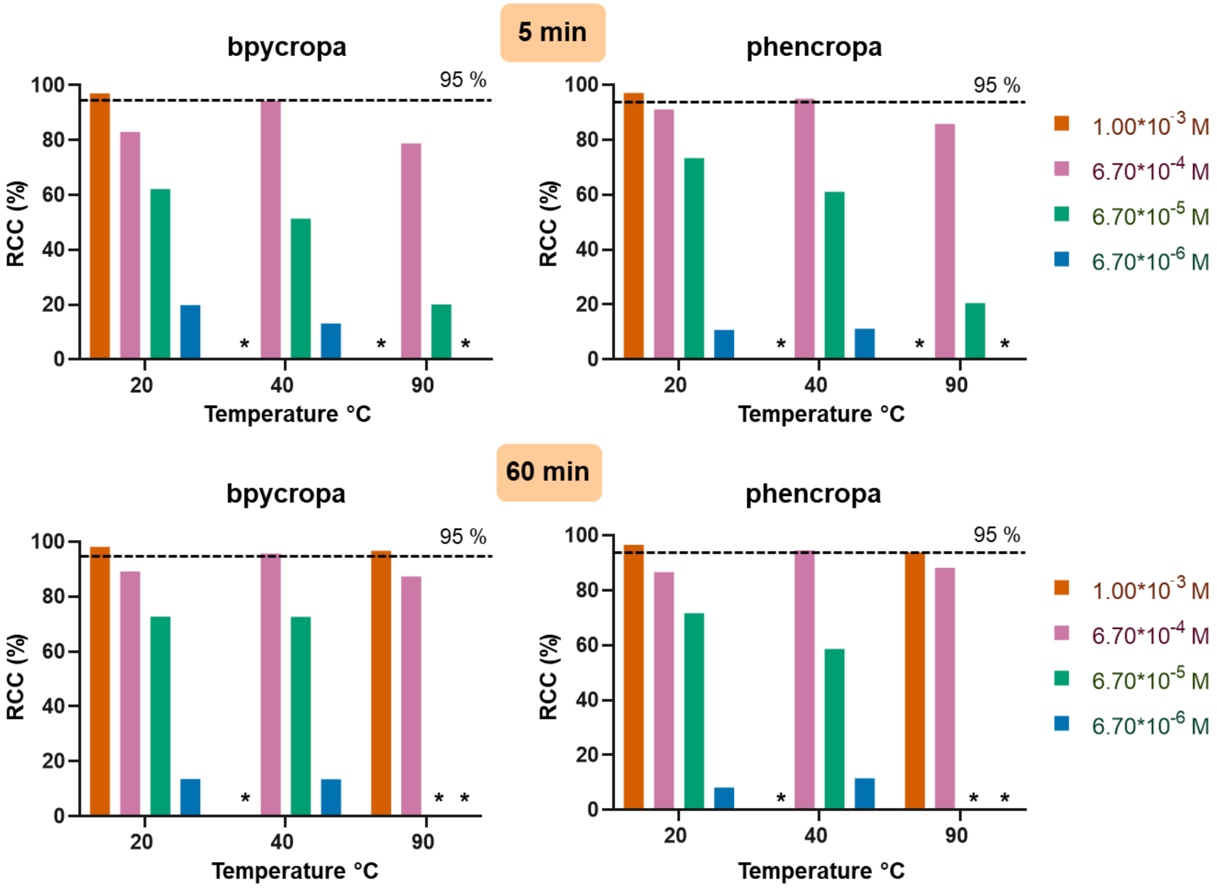

Figure S38. Concentration-dependent ($C_L = 10^{-3}–10^{-6}$ M) and temperature dependent (20, 40, 90 °C) radiochemical incorporation by **bpycropa** (left) and **phencropa** (right) of $[^{223}Ra]RaCl_2$ (pH 6, t = 30 min; 2.7-15.3 kBq ^{223}Ra) at 5 minutes (top) and 60 minutes (bottom). The dashed line indicates 95% RCC. * = not performed.

4.1 Radio-TLC plates and chromatograms

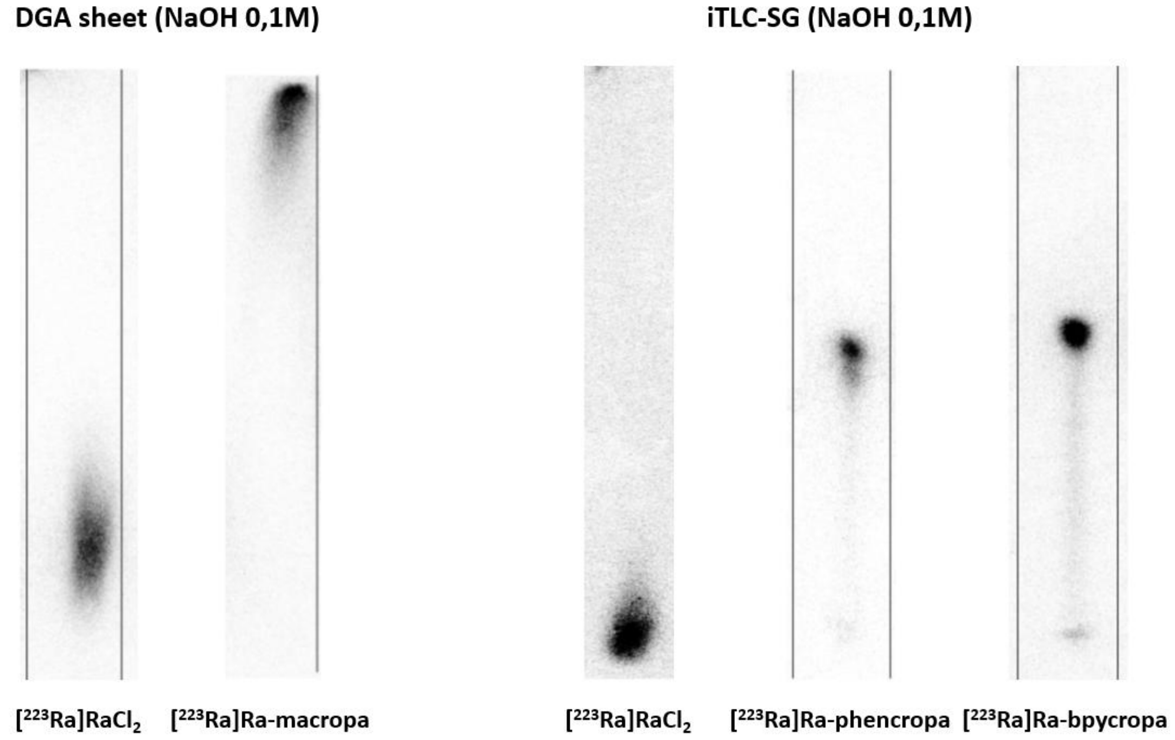

Figure S39. Representative TLC plates for each radiocomplex under optimized conditions.

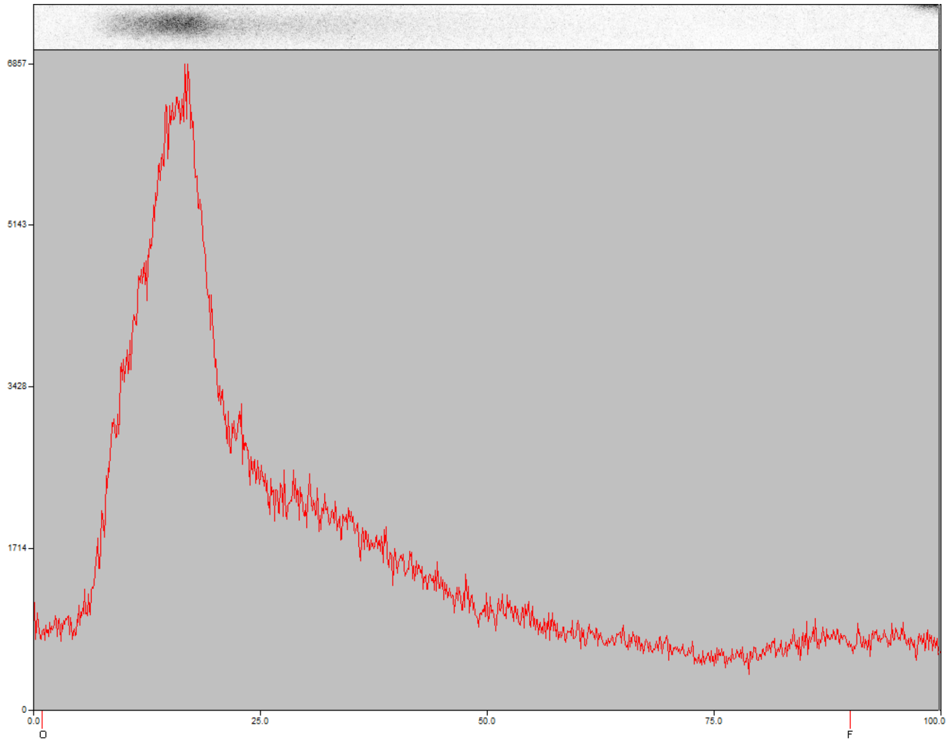

Figure S40. Radio-TLC chromatogram of [^{223}Ra]$RaCl_2$ using iTLC-SG with NaOH 0.1 M as eluent.

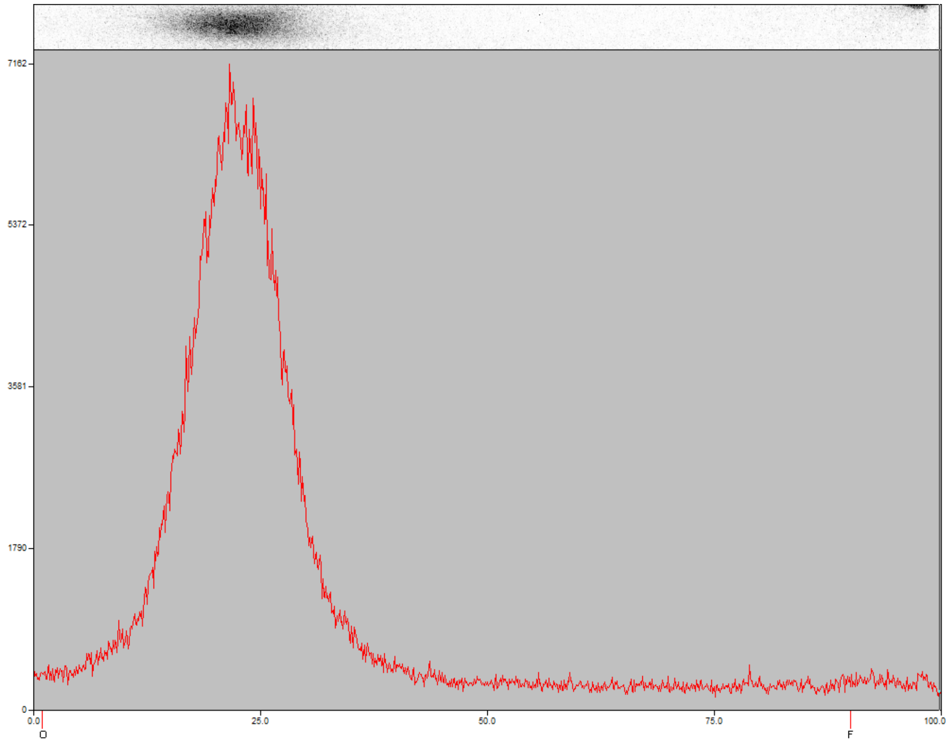

Figure S41. Radio-TLC chromatogram of [^{223}Ra]$RaCl_2$ using DGA sheets with NaOH 0.1 M as eluent.

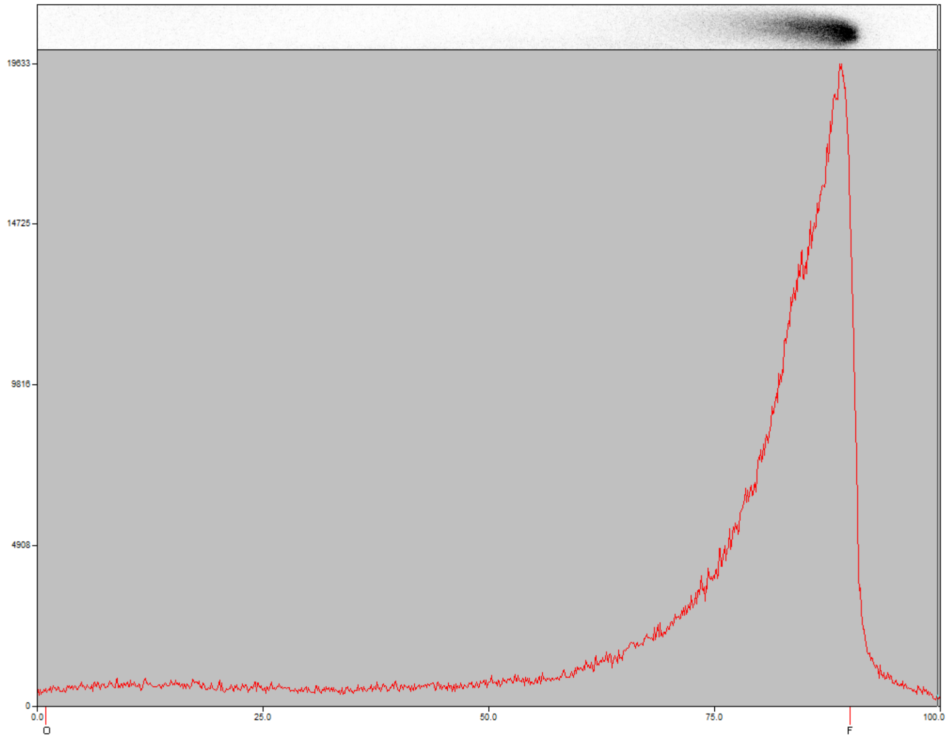

Figure S42. Radio-TLC chromatogram of [^{223}Ra]Ra-**macropa** using DGA sheets with NaOH 0.1 M as eluent.

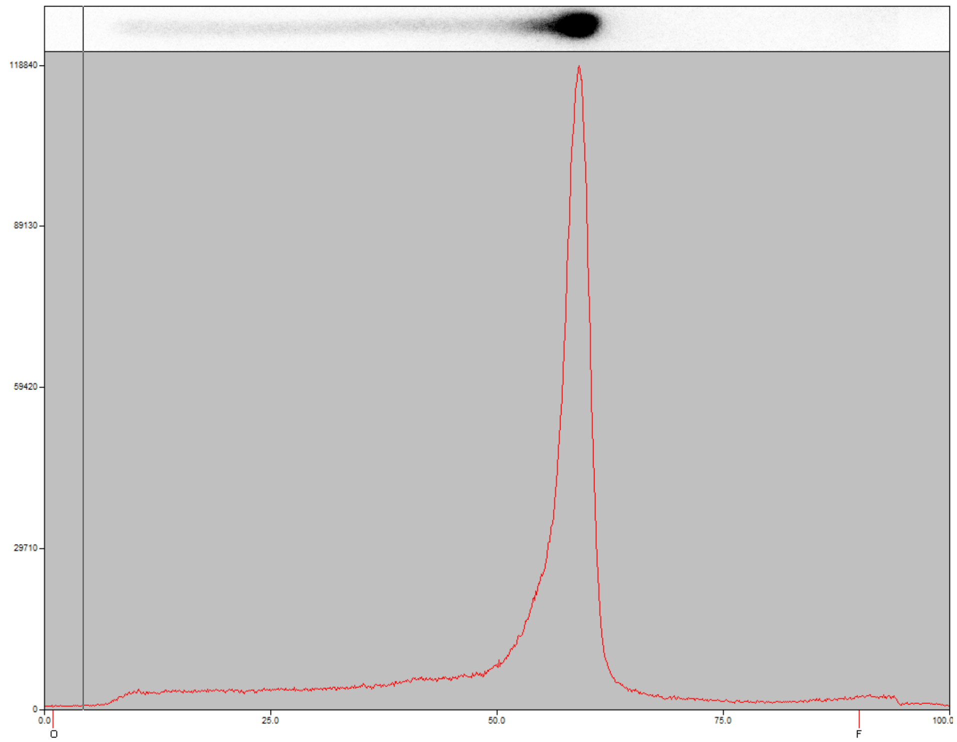

Figure S43. Radio-TLC chromatogram of [^{223}Ra]Ra-**phencropa** using iTLC-SG sheets with NaOH 0.1 M as eluent.

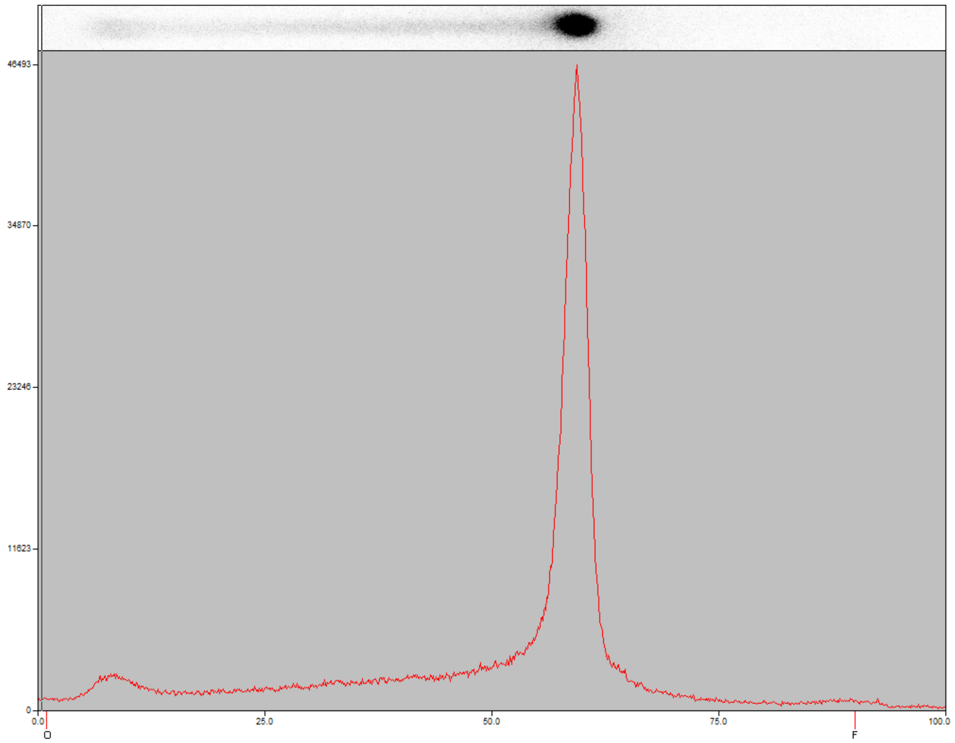

Figure S44. Radio-TLC chromatogram of [^{223}Ra]Ra-**bpycropa** using iTLC-SG sheets with NaOH 0.1 M as eluent.

5. X-ray Crystallography

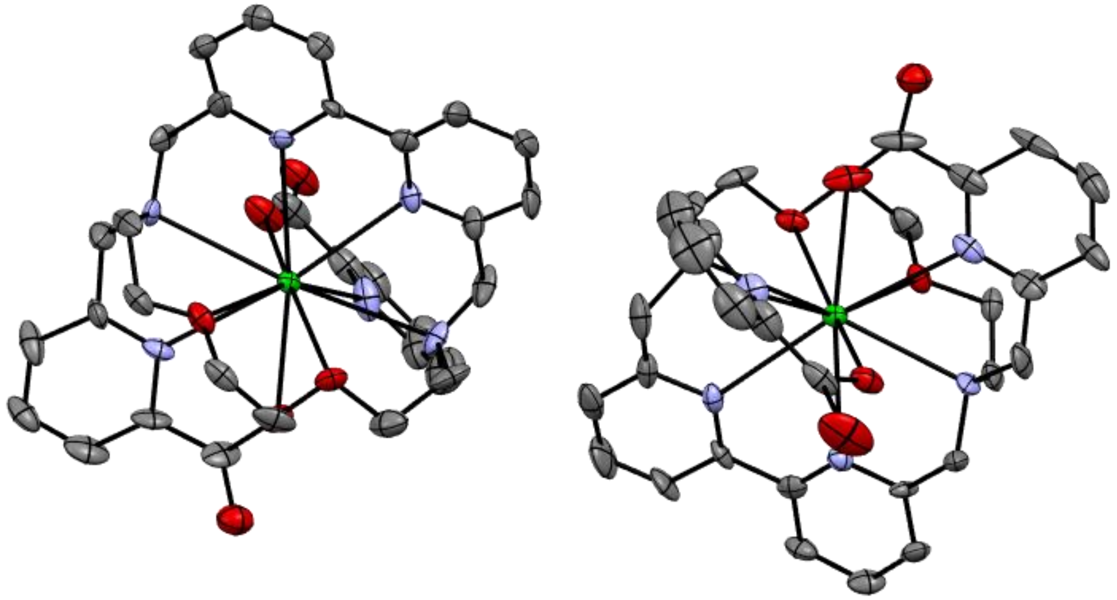

Figure S45. X-ray molecular structure (30% probability ellipsoids) of [Ba(**bpycropa**)] (**4**), showing the two crystallographically independent molecules (Ba1 and Ba2) of the asymmetric unit. Hydrogen atoms and disordered methanol and water molecules have been omitted for clarity

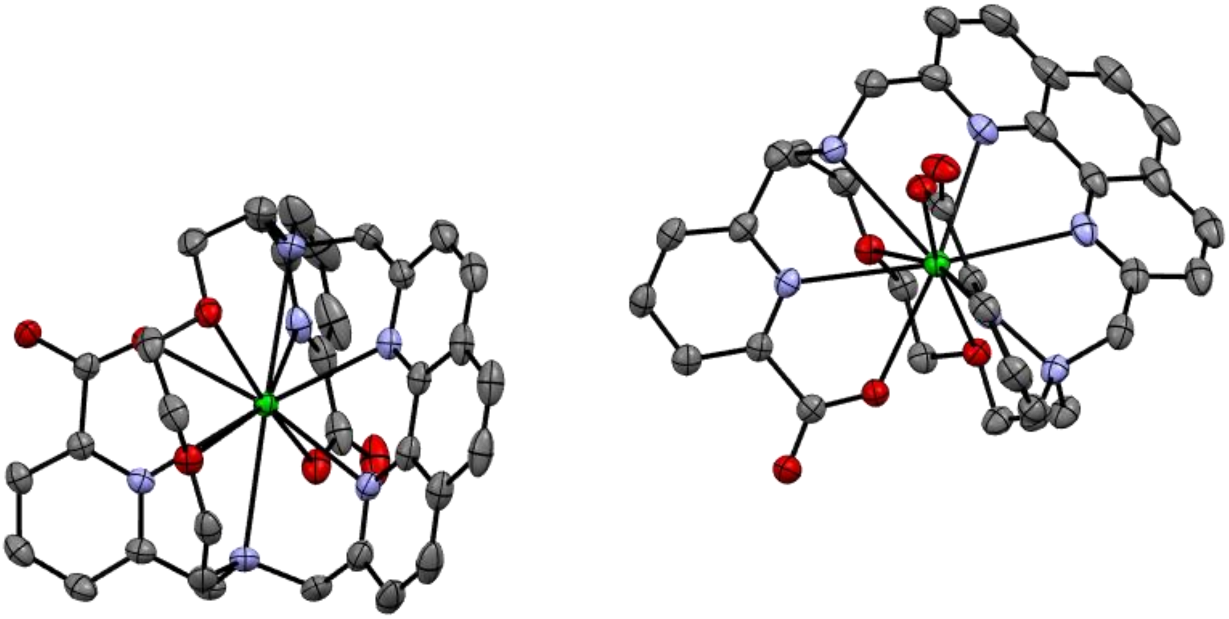

Figure S46. X-ray molecular structure (30% probability ellipsoids) of [Ba(**phencropa**)] (**5**), showing the two crystallographically independent molecules (Ba1 and Ba2) of the asymmetric unit. Hydrogen atoms and disordered methanol and water molecules have been omitted for clarity.

Table S1. Crystal data and data collection of [Ba(**bpycropa**)] (**4**) and [Ba(**phencropa**)] (**5**).

	[Ba(**bpycropa**)] (**4**)	[Ba(**phencropa**)] (**5**)
Formula	$C_{65}H_{72}Ba_2N_{12}O_{12} \cdot CH_3OH \cdot 6.4\ H_2O$	$C_{34}H_{32}BaN_6O_6 \cdot 0.5\ CH_3OH \cdot 3.1\ H_2O$
Formula weight (Da)	807.630	829.4
Temperature (K)	100(2)	100(2)
Wavelength (Å)	0.700	0.700
Crystal System	monoclinic	triclinic
Space Group	P 21	P –1
a (Å)	11.984(3)	14.591(8)
b (Å)	16.317(2)	14.856(2)
c (Å)	17.5630(18)	18.422(3)
α (°)	90	98.831(3)
β (°)	93.710(8)	97.314(12)
γ (°)	90	100.632(11)
V ($Å^3$)	3427.1(11)	3828(2)
Z	4	4
ρ (g cm^{-3})	1.628	1.468
F(000)	1716	1708
μ (mm^{-1})	1.172	1.049
θ_{min}, θ_{max} (°)	1.677, 25.943	1.417, 24.315
Resolution (Å)	0.74	0.71
Total refl. collctd.	40981	40301
Independent refl.	12363	12713
Obs. Refl. $F_o>4\sigma_{Fo}$	11965	11425
I/σ (all data)	38.88	14.41
I/σ (max resltn)	26.35	5.47
R_{merge} (all data)	2.9%	4.7%
R_{merge} (max resltn)	4.3%	19.7%
Completeness (all data)	0.968	0.974
Multiplicity (all data)	5.8	3.0
Multiplicity (max resltn)	5.5	2.5
Data/restraint/parameters	12363/381/924	12713/66/945
$R_{I>2\sigma I}$,$wR_{2,I>2\sigma I}$	0.0504,0.1514	0.0548,0.1569
R (all data), wR_2 (all data)	0.0517,0.1551	0.0590,0.1640
GooF	1.002	1.010
CCDC Nr.	2556698	2556697

6. ESI-MS spectra

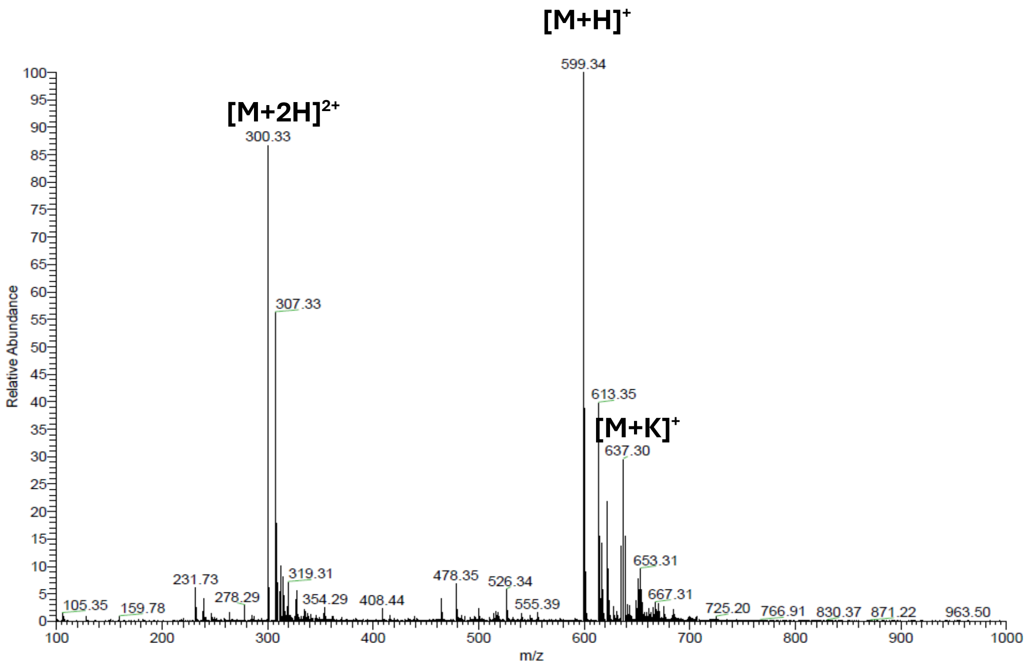

Figure S47. ESI-MS$^{(+)}$ spectrum of **bpycropa**.

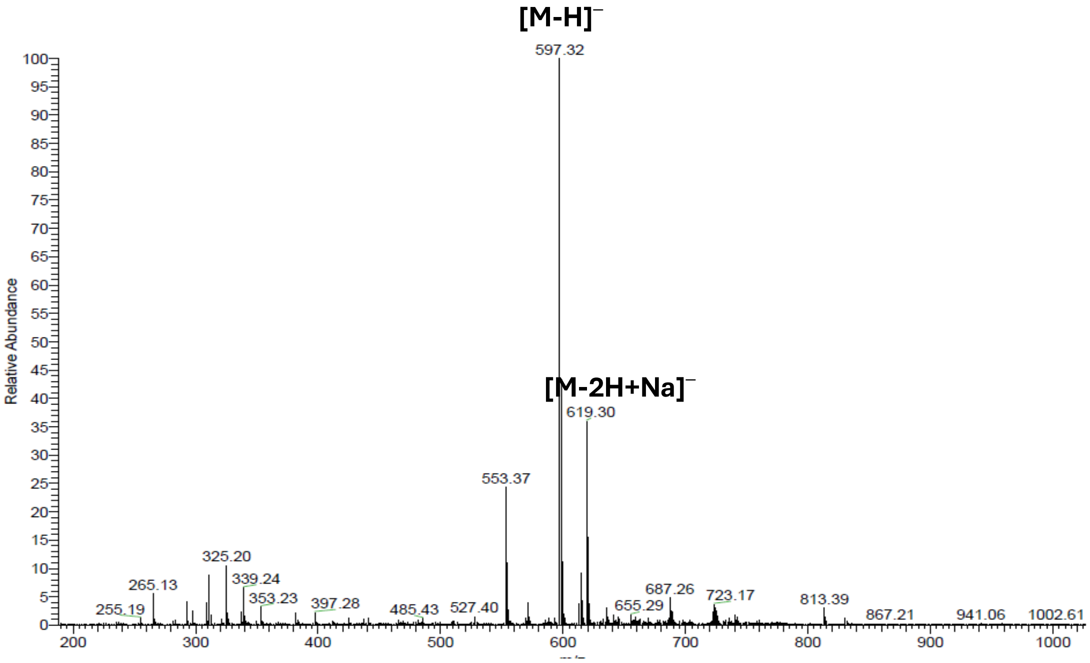

Figure S48. ESI-MS$^{(-)}$ spectrum of **bpycropa**.

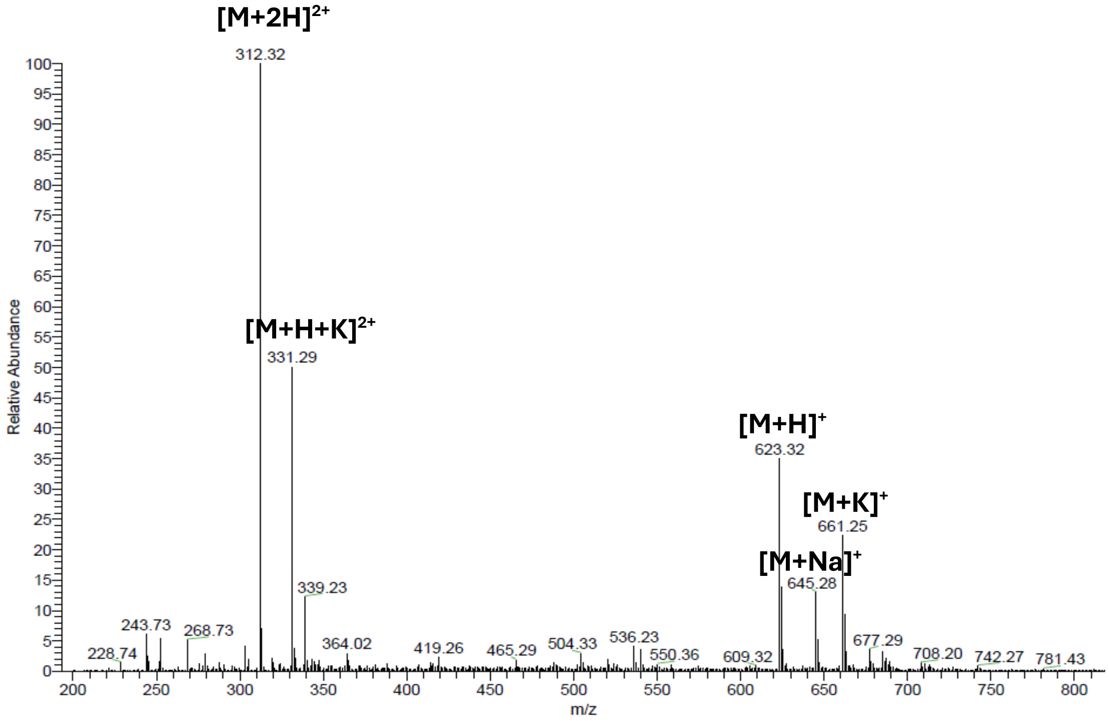

Figure S49. ESI-MS$^{(+)}$ spectrum of **phencropa**.

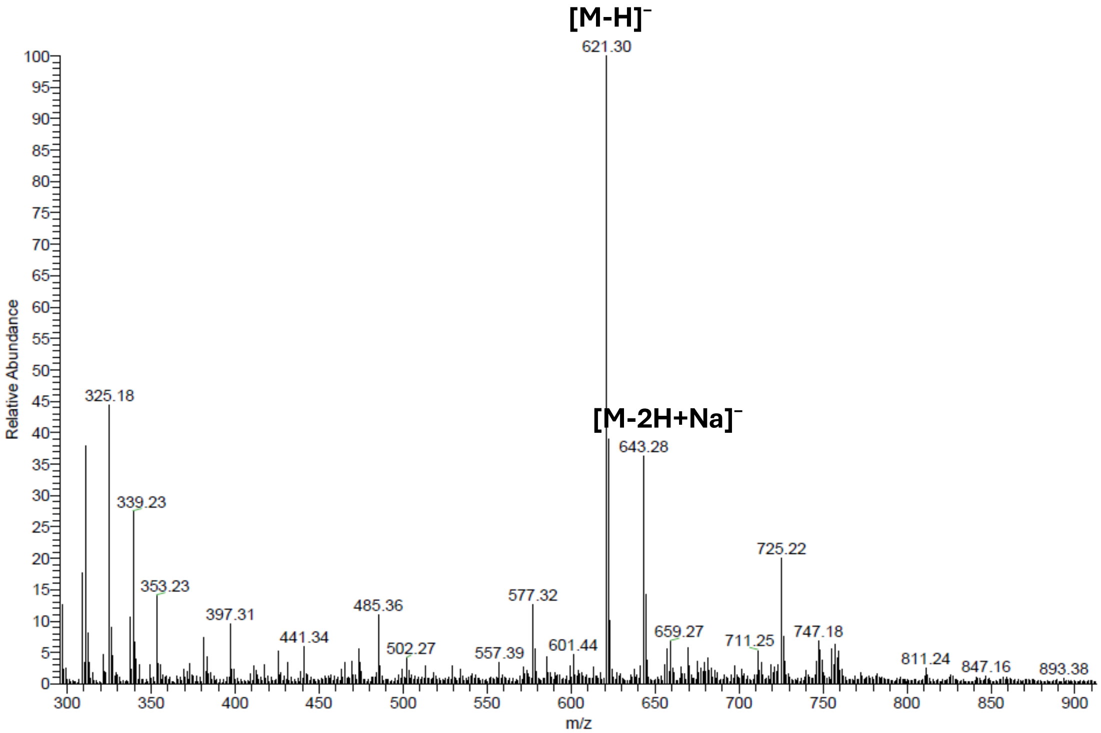

Figure S50. ESI-MS$^{(-)}$ spectrum of **phencropa**.

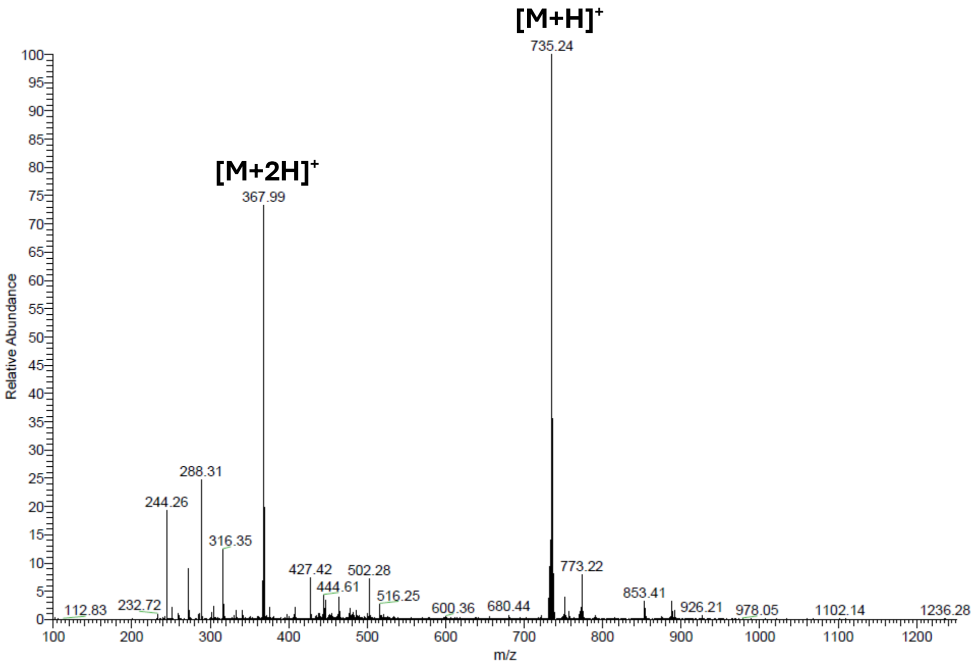

Figure S51. ESI-MS$^+$ spectrum of [Ba(**bpycropa**)] (**4**).

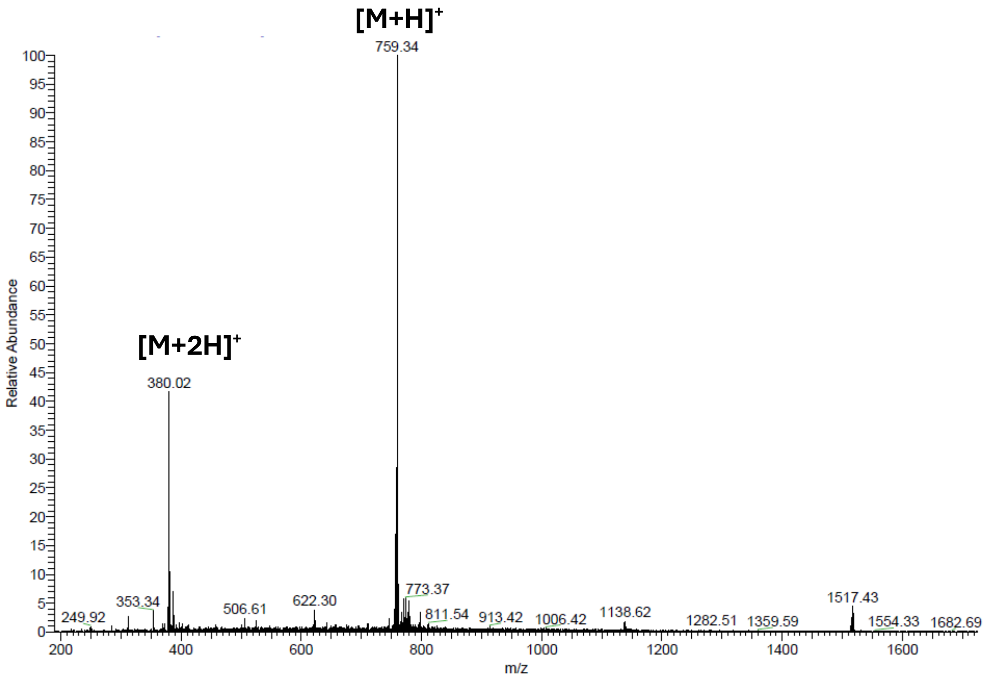

Figure S52. ESI-MS$^+$ spectrum of [Ba(**phencropa**)] (**5**).

7. HR-ESI-MS spectra

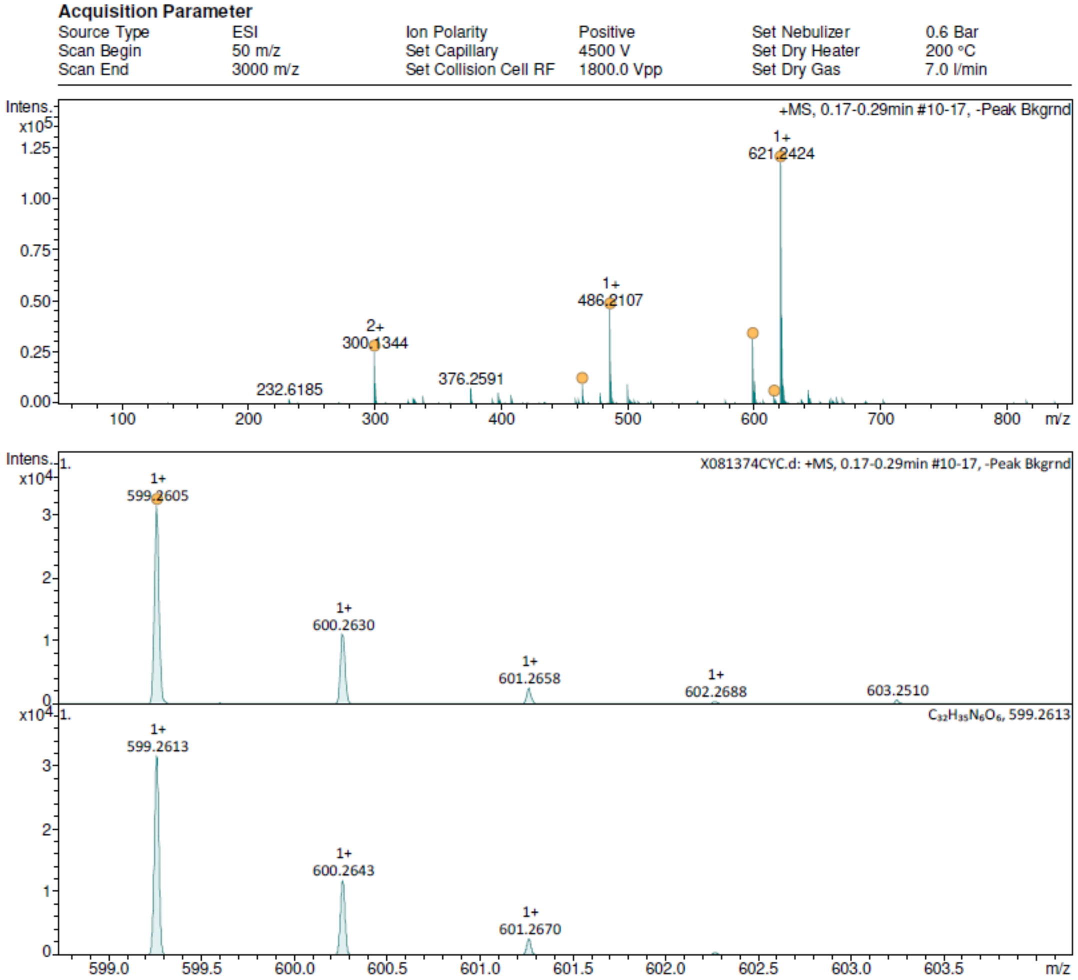

Meas. m/z	z	#	Ion Formula	m/z	err [ppm]	mSigma	rdb	e⁻ Conf
300.134387	2+	1	C32H36N6O6	300.134268	-0.4	19.2	19.0	even
464.228597	1+	1	C24H34NO8	464.227893	-1.5	11.4	9.0	even
	1+	2	C25H30N5O4	464.229231	1.4	21.0	14.0	even
486.210745	1+	1	C25H29N5NaO4	486.211175	0.9	10.2	14.0	even
	1+	2	C24H33NNaO8	486.209838	-1.9	21.4	9.0	even
599.260514	1+	1	C31H39N2O10	599.259922	-1.0	0.5	14.0	even
	1+	2	C32H35N6O6	599.261259	1.2	12.1	19.0	even
616.286019	1+	1	C32H38N7O6	616.287808	2.9	55.0	18.0	even
	1+	2	C31H42N3O10	616.286471	0.7	63.3	13.0	even
621.242360	1+	1	C32H34N6NaO6	621.243204	1.4	2.3	19.0	even
	1+	2	C31H38N2NaO10	621.241866	-0.8	9.6	14.0	even

Figure 53. HR-ESI-MS spectrum of **bpycropa**: *m/z* calcd for $C_{32}H_{34}N_6O_6$: $[M+H]^+$ 599.2613, found 599.2605.

Acquisition Parameter

Source Type	ESI	Ion Polarity	Positive	Set Nebulizer	0.6 Bar
Scan Begin	50 m/z	Set Capillary	4500 V	Set Dry Heater	200 °C
Scan End	3000 m/z	Set Collision Cell RF	1800.0 Vpp	Set Dry Gas	7.0 l/min

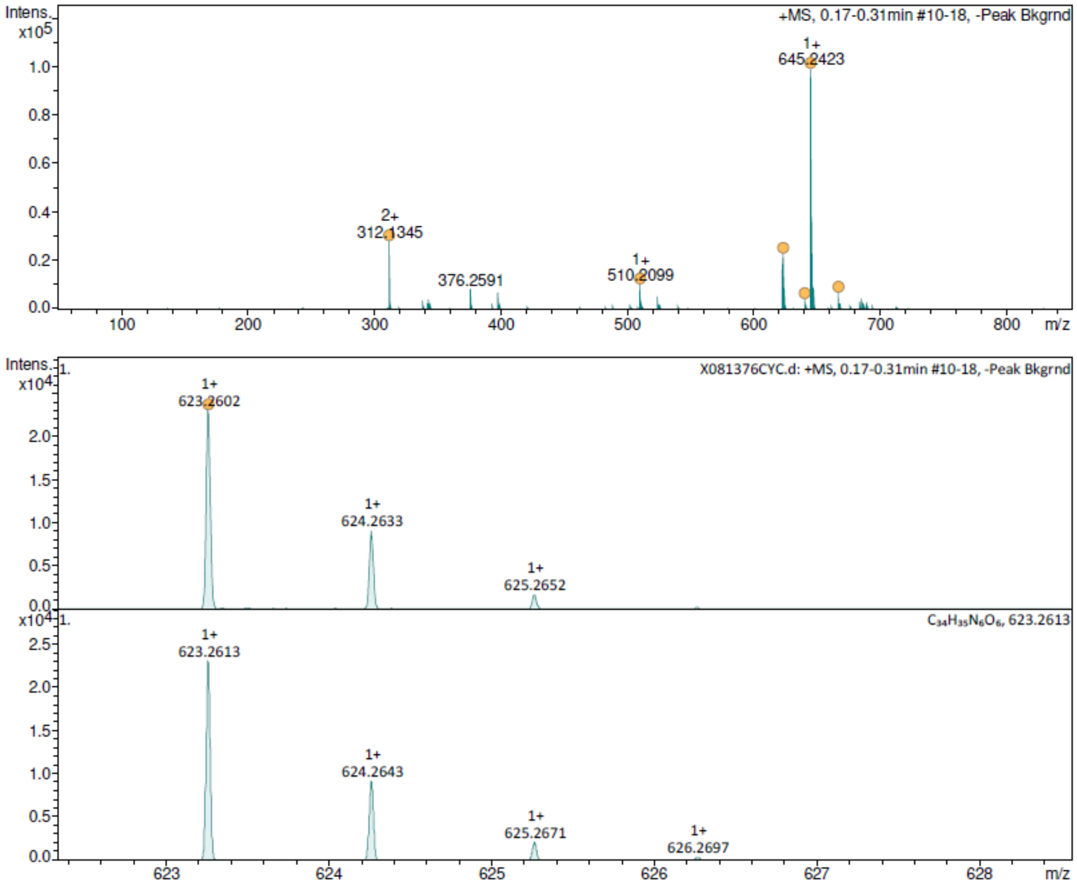

Meas. m/z	z	#	Ion Formula	m/z	err [ppm]	mSigma	rdb	e⁻ Conf
312.134543	2+	1	C34H36N6O6	312.134268	-0.9	23.3	21.0	even
510.209886	1+	1	C24H28N7O6	510.209558	-0.6	35.0	15.0	even
623.260177	1+	1	C34H35N6O6	623.261259	1.7	11.6	21.0	even
	1+	2	C33H39N2O10	623.259922	-0.4	15.8	16.0	even
640.286175	1+	1	C34H38N7O6	640.287808	2.6	39.9	20.0	even
	1+	2	C33H42N3O10	640.286471	0.5	50.8	15.0	even
645.242258	1+	1	C34H34N6NaO6	645.243204	1.5	4.1	21.0	even
	1+	2	C33H38N2NaO10	645.241866	-0.6	8.3	16.0	even
667.224300	1+	1	C33H37N2Na2O10	667.223810	-0.7	7.9	16.0	even
	1+	2	C34H33N6Na2O6	667.225148	1.3	14.9	21.0	even

Figure S54. HR-ESI-MS spectrum of **phencropa**. *m/z* calcd for $C_{34}H_{36}N_6O_6$: $[M+H]^+$ 623.2601, $[M+Na]^+$ 645.2421, $[M+2H]^{2+}$ 312.1345; found $[M+H]^+$ 623.2602, $[M+Na]^+$ 645.2423, $[M+2H]^{2+}$ 312.1345.

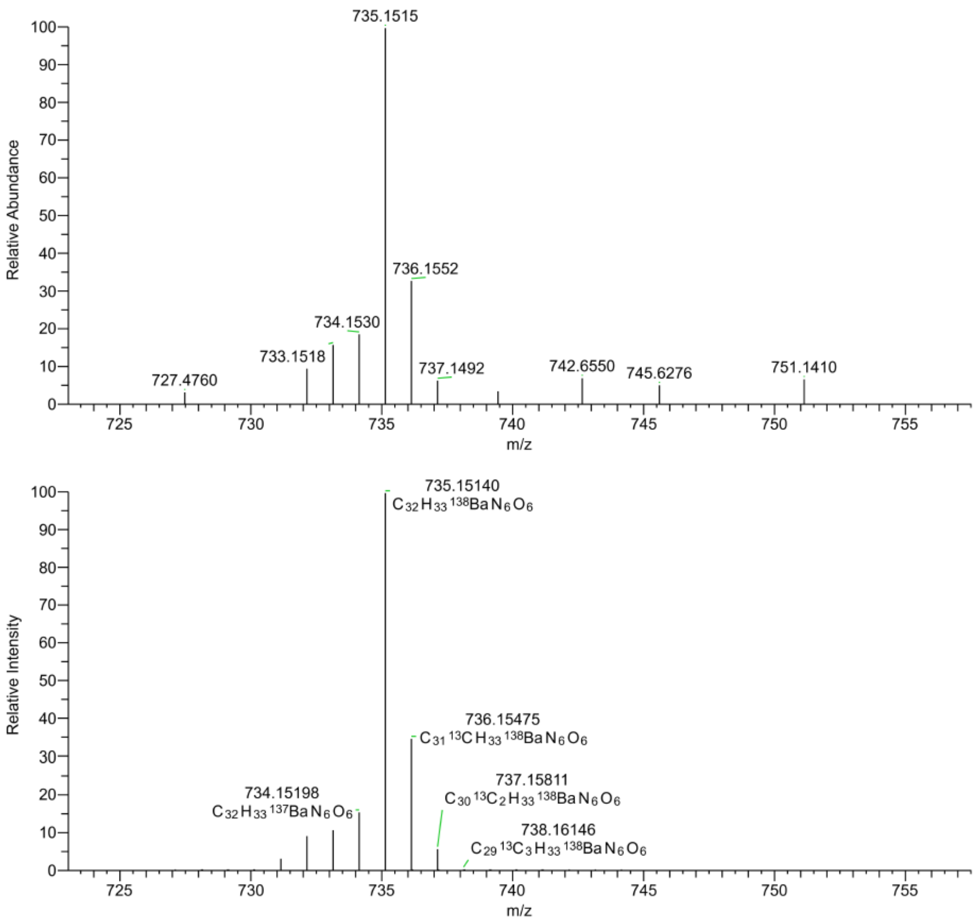

Figure S55. HR-ESI-MS spectrum of ($[M+H]^+$) from [Ba(**bpycropa**)] (**4**). Experimental spectrum (top), calculated spectrum (bottom).

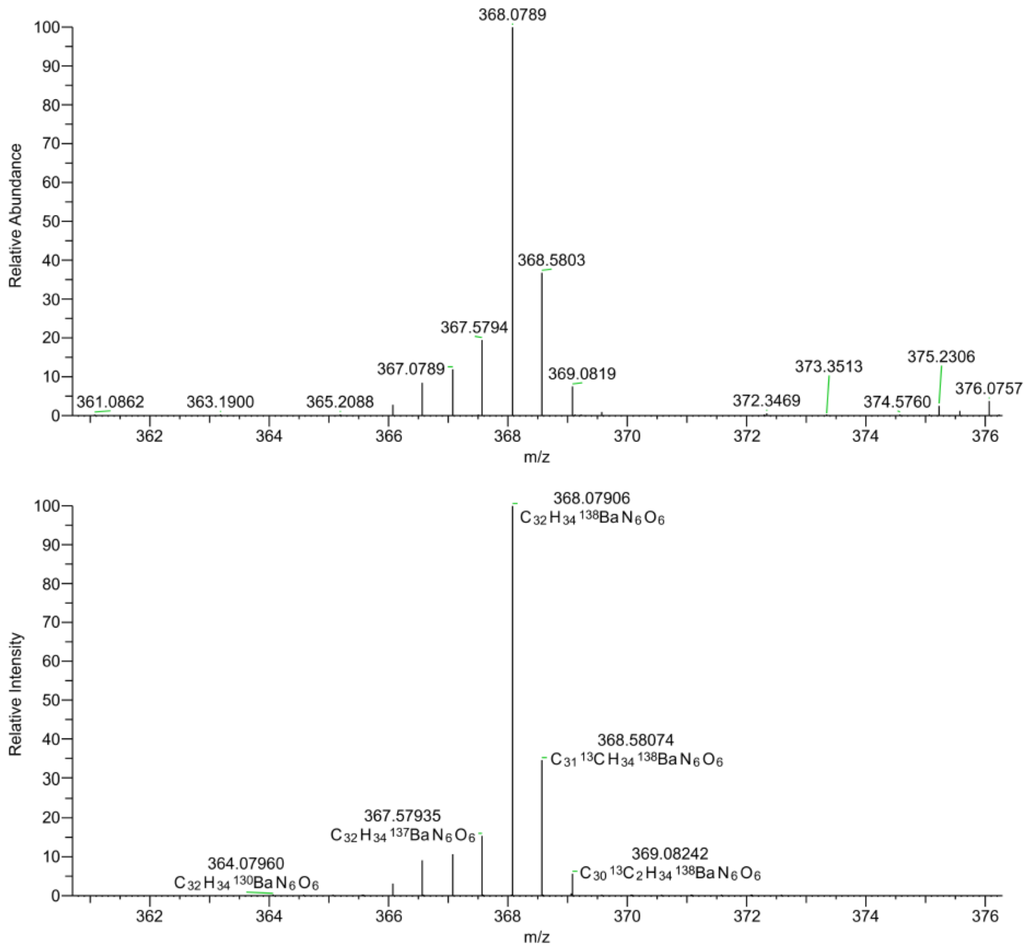

Figure S56. HR-ESI-MS spectrum of ([M+2H]$^{2+}$ from [Ba(**bpycropa**)] (**4**). Experimental spectrum (top), calculated spectrum (bottom).

Acquisition Parameter

Source Type	ESI	Ion Polarity	Positive	Set Nebulizer	0.6 Bar
Scan Begin	50 m/z	Set Capillary	4500 V	Set Dry Heater	200 °C
Scan End	3000 m/z	Set Collision Cell RF	1800.0 Vpp	Set Dry Gas	7.0 l/min

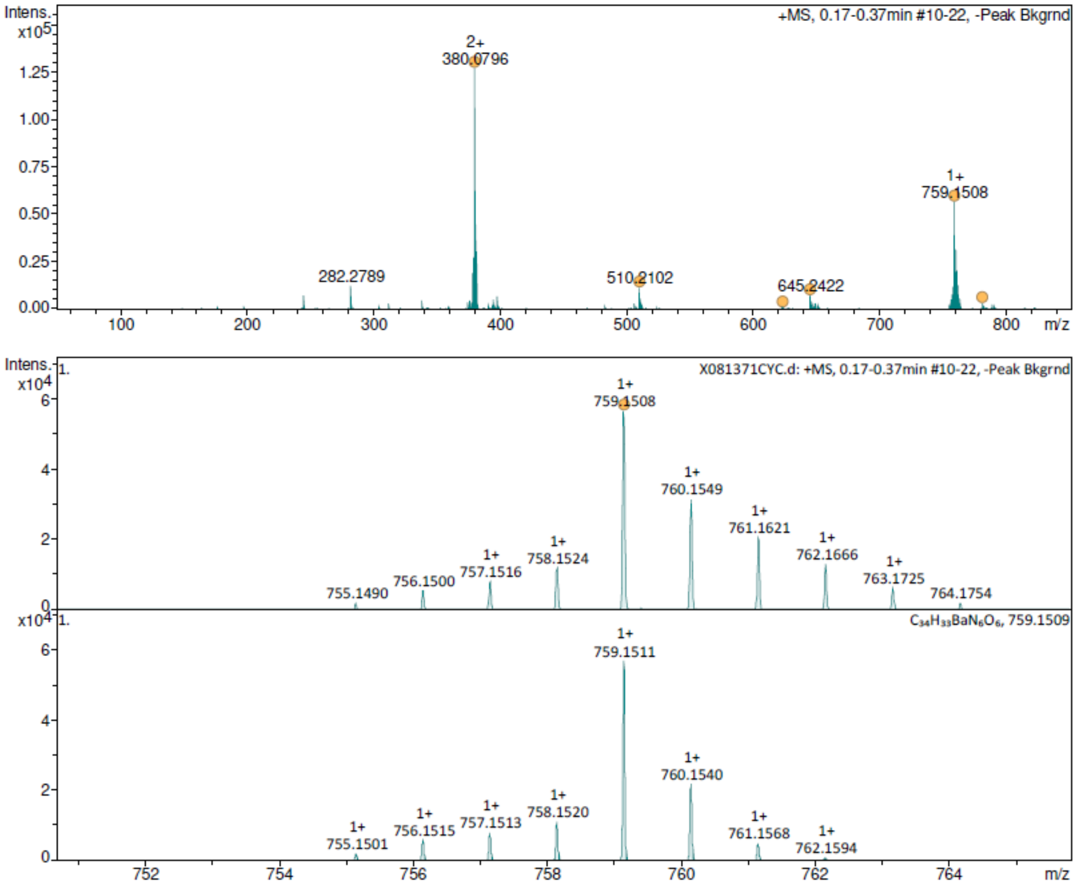

Meas. m/z	z	#	Ion Formula	m/z	err [ppm]	mSigma	rdb	e⁻ Conf
380.079614	2+	1	C34H34BaN6O6	380.079066	-1.1	137.4	22.0	even
	2+	2	C21H42BaN4O17	380.079323	-0.5	167.9	4.0	even
510.210168	1+	1	C27H29N5NaO4	510.211175	2.0	125.3	16.0	even
623.259775	1+	1	C34H35N6O6	623.261259	2.4	124.9	21.0	even
	1+	2	C33H39N2O10	623.259922	0.2	135.8	16.0	even
645.242173	1+	1	C34H34N6NaO6	645.243204	1.6	164.1	21.0	even
	1+	2	C33H38N2NaO10	645.241866	-0.5	171.1	16.0	even
759.150800	1+	1	C34H33BaN6O6	759.150856	0.4	137.6	22.0	even
	1+	2	C33H37BaN2O10	759.149519	-1.3	140.8	17.0	even
	1+	3	C21H41BaN4O17	759.151369	1.0	168.3	4.0	even
781.132674	1+	1	C34H32BaN6NaO6	781.132801	0.5	137.3	22.0	even
	1+	2	C33H36BaN2NaO10	781.131463	-1.2	139.6	17.0	even
	1+	3	C21H40BaN4NaO17	781.133314	1.0	162.8	4.0	even

Figure S57. HR-ESI-MS spectrum of [Ba(**phencropa**)] (**5**). *m/z* calcd for $C_{34}H_{36}N_6O_6$: $[M+H]^+$ 759.1508; found 759.1508.

8. DFT geometries

8.1 Sample input files

Geometry Optimization and Frequency Calculation

```
%mem=25gb
%chk=PBE0_BaBipyO2DPA_I3.chk
# rpbe1pbe/gen pseudo=cards scrf=(pcm,solvent=water) scf=tight integral=superfinegrid opt
freq=noraman EmpiricalDispersion=GD3BJ

PBE0_BaBipyO2DPA_I3

0  1
 Ba      0.55517800   -0.19276000   -0.62756900
 O      -1.09246300    2.21817000   -0.85693400
 O       1.33013600    2.13956900   -2.25791400
 N       0.31076500    0.77714800    2.12685400
 O       0.16782300   -2.43514300    0.75425300
 O      -6.26772200    1.06819400   -2.61016700
 N       2.78774200    1.08811300    0.89264900
 N      -2.00309800   -0.00800100    0.75953400
 N       3.35463600    0.03929600   -1.84663000
 N      -4.82055500   -0.42404400   -0.81398900
 C       1.25814700    1.51311400    2.71409200
 C      -0.76724100    0.42637300    2.82853400
 N       2.41180800   -2.42379700   -0.72866100
 C       2.44570100    1.88925500    1.90767700
 C       3.91283500    1.34123700    0.22224000
 C      -0.93279700    0.78618200    4.16038600
 C       0.62803100    3.34411700   -2.03082300
 C      -0.84076900    3.05148000   -1.96613800
 C       1.17051200    1.89793800    4.04904200
 C       0.05825100    1.52356500    4.78047200
 C      -2.63838500    1.30285100    0.68716000
 C      -2.47276700    1.98311800   -0.64478600
 C       4.72267000    2.43202800    0.51365000
 C      -4.13260200   -1.13770500    0.07537400
 C       3.33888000    1.04547800   -2.90685800
 C      -1.80648700   -0.41498300    2.13911000
 O       0.75553800   -4.36045500    1.72619900
 O      -8.17582800    0.39525700   -1.65338200
 C       4.34150000    0.34075800   -0.81449100
 C       2.69779200    2.35618200   -2.53587400
 C      -2.64118900   -1.04481000   -0.05778100
 C       3.18963600    3.01943500    2.23753900
 C       0.93471200   -3.41719900    0.94483900
 C      -6.93041300    0.43148100   -1.76844800
```

C	-6.15127900	-0.40712300	-0.74251500
C	4.34066600	3.29508400	1.52406700
C	-4.74459800	-1.88060600	1.07797500
C	3.51268900	-2.40261100	-1.47846000
C	-6.84081800	-1.11996500	0.23476900
C	3.65293400	-1.26097200	-2.44426700
C	4.27050700	-4.45972900	-0.53236400
C	2.21518600	-3.43605800	0.11489400
C	-6.12779200	-1.86852600	1.15482900
C	3.12368000	-4.47865800	0.24134800
C	4.47250100	-3.40318500	-1.40591800
H	-7.92096500	-1.07499000	0.24730600
H	-6.64129600	-2.43916100	1.92055600
H	-4.14862300	-2.46054500	1.77255200
H	-2.44330800	-0.83681100	-1.11256500
H	-2.15674700	-1.98908200	0.19533500
H	-1.44602300	-1.44787700	2.13056200
H	-2.73539400	-0.38374000	2.72612400
H	-2.89247000	1.38443300	-1.46099600
H	-3.01208300	2.93801900	-0.62018200
H	-1.82749800	0.48866700	4.69290800
H	-0.03353300	1.80802300	5.82177100
H	1.96662000	2.46102200	4.51574600
H	2.86721800	3.67926800	3.03098300
H	4.93347300	4.17147300	1.75642100
H	5.62608800	2.60288100	-0.05812700
H	2.91299700	-5.27520100	0.94156300
H	5.00340800	-5.25430100	-0.45433400
H	5.36157700	-3.34788800	-2.02197200
H	2.94559000	-1.41833000	-3.26420200
H	4.66062500	-1.27961800	-2.88561100
H	4.55180700	-0.58732800	-0.27569600
H	5.29002600	0.66843000	-1.26357000
H	-1.38756100	3.99699900	-1.86107600
H	-1.17275400	2.56500700	-2.89284800
H	0.81423200	4.04931300	-2.85019700
H	0.96699300	3.80673600	-1.09447900
H	2.79792900	3.03807200	-3.38981000
H	3.18585800	2.83155200	-1.67818100
H	4.35924000	1.25074000	-3.26747500
H	2.77785600	0.63050100	-3.74762800
H	-3.70910200	1.25172800	0.92564700
H	-2.17126700	1.93557800	1.44494100

Ba 0
S 5 1.00

114.075390 0.001868
52.874016 -0.008752
25.187082 0.029840
12.287174 -0.086380
5.950803 0.218765
S 1 1.00
2.807950 1.0
S 1 1.00
1.303956 1.0
S 1 1.00
0.565336 1.0
S 1 1.00
0.251764 1.0
S 1 1.00
0.104792 1.0
S 1 1.00
0.045562 1.0
S 1 1.00
0.020317 1.0
S 1 1.00
0.008330 1.0
P 4 1.00
51.225054 -0.000088
24.862306 0.000464
12.277196 -0.003409
5.958786 0.032871
P 1 1.00
2.810868 1.0
P 1 1.00
1.364893 1.0
P 1 1.00
0.642352 1.0
P 1 1.00
0.273064 1.0
P 1 1.00
0.106254 1.0
P 1 1.00
0.028185 1.0
P 1 1.00
0.010657 1.0
P 1 1.00
0.004633 1.0
D 1 1.00
1.303956 1.0
D 1 1.00
0.565336 1.0

D 1 1.00
0.251764 1.0
D 1 1.00
0.104792 1.0
D 1 1.00
0.045562 1.0
D 1 1.00
0.020317 1.0
F 1 1.00
1.364893 1.0
F 1 1.00
0.642352 1.0
F 1 1.00
0.273064 1.0
F 1 1.00
0.106254 1.0
G 1 1.00
0.565336 1.0
G 1 1.00
0.251764 1.0
****
C H N O 0
def2tzvp
****

Ba 0
ECP46MDF 5 46
H-Komponente
1
2 1. 0.
S-H
2
2 4.177931587 84.785457583
2 2.522632800 17.372709041
P-H
4
2 6.294119351 52.512225743
2 6.476457746 105.022668647
2 2.284326647 8.707014937
2 2.091555201 17.165458832
D-H
4
2 1.925291745 5.346535679
2 1.878534118 8.025720742
2 0.907088727 1.346295081
2 0.910060953 2.063710453

F-H
4
2 6.256321669 -20.003223472
2 6.134135837 -26.118214748
2 1.641382784 -2.344457989
2 1.599343316 -2.980867480
G-H
2
2 2.142381001 -3.316602759
2 2.159981109 -4.275647018

Transition State Geometry Optimization and Frequency Calculation

%mem=25gb
%chk=TS_PBE0_BaBipyO2DPA_I3_I4.chk
rpbe1pbe/gen pseudo=cards scrf=(pcm,solvent=water) scf=tight integral=superfinegrid opt=QST3 freq=noraman EmpiricalDispersion=GD3BJ

PBE0_BaBipyO2DPA_I3

0 1

Ba	-0.71117800	0.18284100	-0.72685300
O	0.79179400	-2.32202800	-1.14206700
O	-1.80801700	-2.09293600	-2.20279000
N	-0.11335900	-0.88837800	1.91381800
O	-0.03772300	2.37445600	0.63109200
O	6.89935900	-1.42291500	-1.97585400
N	-2.75008900	-1.05690000	1.04088400
N	2.08631700	-0.14300800	0.27046800
N	-3.64373600	0.10997700	-1.55538400
N	5.08666800	0.30979600	-0.84727900
C	-0.99170400	-1.62617100	2.59872800
C	1.07103400	-0.61169400	2.46057500
N	-2.44607000	2.51458600	-0.55711300
C	-2.30433400	-1.91283100	1.96742900
C	-3.97418200	-1.22702900	0.53924800
C	1.41969100	-1.05519200	3.73061500
C	-1.15860800	-3.33716000	-2.03633000
C	0.32115000	-3.14646400	-2.18511400
C	-0.71924200	-2.09079800	3.88267700
C	0.50483600	-1.79685600	4.45408700
C	2.63526700	-1.48597600	0.10229100
C	2.19670400	-2.15040300	-1.17460400
C	-4.79312000	-2.28293700	0.92032400
C	4.21187600	1.10503700	-0.22967300
C	-3.80802800	-0.87394000	-2.62381700
C	2.03086700	0.23619800	1.67309700

O	-0.35198400	4.35317300	1.62245400
O	8.58624500	-0.23233900	-1.11133300
C	-4.49193500	-0.17536900	-0.40251300
C	-3.20648900	-2.22667400	-2.34807600
C	2.75630400	0.87917000	-0.54459300
C	-3.05186900	-3.01740600	2.36834000
C	-0.70360600	3.40968400	0.90221700
C	7.37651700	-0.49494000	-1.29481200
C	6.38610100	0.44045600	-0.58297500
C	-4.31222900	-3.20358000	1.83271400
C	4.60665300	2.07888600	0.68143200
C	-3.64614100	2.54781200	-1.13400400
C	6.85926400	1.39187600	0.31651100
C	-3.96749500	1.43292400	-2.08774300
C	-4.18006000	4.60322100	-0.04312400
C	-2.08904100	3.49753900	0.26789200
C	5.95645700	2.22172900	0.95735000
C	-2.92983500	4.56664400	0.54837900
C	-4.54856400	3.57588500	-0.89719900
H	7.92445300	1.46176200	0.48843300
H	6.29785800	2.97318800	1.66053700
H	3.86747200	2.71326000	1.15635700
H	2.67728100	0.58514300	-1.59359100
H	2.19691400	1.80744400	-0.40630200
H	1.67912800	1.27201800	1.71678100
H	3.01538800	0.19331300	2.15915200
H	2.48359400	-1.57017700	-2.06100000
H	2.68746800	-3.12867300	-1.24913500
H	2.39508000	-0.81765100	4.13627700
H	0.74101500	-2.14530700	5.45229600
H	-1.45818200	-2.65255700	4.43665700
H	-2.65120900	-3.72658500	3.07906900
H	-4.91193100	-4.05687700	2.12543100
H	-5.78061000	-2.38480500	0.48813700
H	-2.58871100	5.33864400	1.22416600
H	-4.86478200	5.41799200	0.16153800
H	-5.52207200	3.56384000	-1.37166200
H	-3.37462800	1.57823100	-2.99581200
H	-5.02500500	1.49976700	-2.38398900
H	-4.57797000	0.74564300	0.18093800
H	-5.50897800	-0.44468300	-0.72169500
H	0.81166000	-4.12725100	-2.14251600
H	0.54632300	-2.69390900	-3.16007200
H	-1.50006100	-4.04489000	-2.80149600
H	-1.39525700	-3.75598900	-1.04938600
H	-3.42867500	-2.87809100	-3.20275100

H	-3.63434000	-2.69877200	-1.45745300
H	-4.87314400	-1.01625100	-2.86631700
H	-3.32530800	-0.47204900	-3.51799200
H	3.73125100	-1.48732500	0.14086700
H	2.27897800	-2.09914600	0.93252300

PBE0_BaBipyO2DPA_I4

0 1

Ba	0.85370400	0.17368500	-0.52749300
O	-0.36506100	-0.20369300	2.02919100
O	2.38000000	0.49828500	1.89405800
N	-0.00650400	-2.68815300	-0.55007000
O	-0.37489400	2.42580300	0.20524400
O	-6.48338400	0.88343400	2.28681100
N	2.70799800	-2.21674100	-0.59019900
N	-2.03161400	-0.64496000	-0.33650500
N	3.88068700	0.54129200	-0.60875900
N	-4.93992400	0.67409900	0.00869400
C	0.82640600	-3.68383700	-0.23368700
C	-1.29343200	-2.96065100	-0.76294600
N	2.13860700	2.77771200	-0.68896100
C	2.26390100	-3.35560900	-0.05196200
C	4.01041300	-1.93685500	-0.53924400
C	-1.80689100	-4.24729000	-0.65886400
C	1.73850100	0.08321400	3.08413500
C	0.30188400	0.50997000	3.04932800
C	0.38755000	-5.00150600	-0.12541400
C	-0.94860700	-5.28268900	-0.33833300
C	-2.41348700	-0.90978600	1.05018800
C	-1.76427200	0.03266500	2.02723100
C	4.93050800	-2.78256600	0.06464500
C	-4.20440100	0.39468900	-1.06918100
C	4.43077600	0.82379600	0.71851200
C	-2.19491600	-1.82621600	-1.16797700
O	-0.63299500	4.62665400	0.50042400
O	-8.31416900	1.03872900	1.00628900
C	4.45116200	-0.66359100	-1.20308100
C	3.74204400	0.11720800	1.86021500
C	-2.71091200	0.52165000	-0.91942200
C	3.11936400	-4.22985500	0.61703300
C	0.01724700	3.62264000	0.17888400
C	-7.09091100	0.85844000	1.19892100
C	-6.26525600	0.55672700	-0.05934100
C	4.46886500	-3.93769700	0.67093500
C	-4.77373900	-0.01753800	-2.26927500

C	3.40482400	2.91723300	-1.07605500
C	-6.90958800	0.16297700	-1.22912400
C	4.12074800	1.66834500	-1.50868200
C	3.32023600	5.27343600	-0.70300200
C	1.45135100	3.84789500	-0.29430200
C	-6.15191000	-0.13387500	-2.34767900
C	2.00748600	5.12047200	-0.29273600
C	4.03650800	4.15340700	-1.09440000
H	-7.98919400	0.10118000	-1.23556900
H	-6.62625000	-0.44869900	-3.27052200
H	-4.14502500	-0.23559100	-3.12476000
H	-2.47875700	1.38802900	-0.30036400
H	-2.26339000	0.69509800	-1.90182200
H	-1.95077100	-1.54625800	-2.19911900
H	-3.23127600	-2.19128300	-1.17951700
H	-1.95672200	1.08160500	1.77661100
H	-2.16737200	-0.16165100	3.02857500
H	-2.86120300	-4.42481400	-0.82933800
H	-1.31354200	-6.29959600	-0.25941700
H	1.07939700	-5.80106500	0.09771700
H	2.73964700	-5.12215800	1.09433600
H	5.15252300	-4.60420900	1.18258100
H	5.98118400	-2.52193600	0.07757100
H	1.40507200	5.95602900	0.03606700
H	3.78765600	6.25127500	-0.70636900
H	5.07228400	4.23012600	-1.40176000
H	5.19461900	1.88162500	-1.61471800
H	3.75454800	1.38403700	-2.50059700
H	4.12359100	-0.70368700	-2.24547100
H	5.55023900	-0.61864400	-1.20765000
H	-0.15537800	0.29008500	4.02194700
H	0.22517900	1.58913000	2.86560400
H	2.22968100	0.54069000	3.95160700
H	1.80451000	-1.00797900	3.18635500
H	4.23435300	0.42366300	2.79181100
H	3.82001300	-0.97272000	1.79188600
H	5.50470800	0.58748200	0.75825000
H	4.33470300	1.89550300	0.90040300
H	-3.50017000	-0.86619600	1.18764900
H	-2.09174000	-1.92304600	1.30017200

TS_PBE0_BaBipyO2DPA_I3_I4

0 1

Ba	-0.71117800	0.18284100	-0.72685300
O	0.79179400	-2.32202800	-1.14206700

O	-1.80801700	-2.09293600	-2.20279000
N	-0.11335900	-0.88837800	1.91381800
O	0.49366760	1.34284409	-0.13541923
O	6.89935900	-1.42291500	-1.97585400
N	-2.75008900	-1.05690000	1.04088400
N	2.08631700	-0.14300800	0.27046800
N	-3.64373600	0.10997700	-1.55538400
N	5.08666800	0.30979600	-0.84727900
C	-0.99170400	-1.62617100	2.59872800
C	1.07103400	-0.61169400	2.46057500
N	-1.88628912	2.14720654	-1.09479917
C	-2.30433400	-1.91283100	1.96742900
C	-3.97418200	-1.22702900	0.53924800
C	1.41969100	-1.05519200	3.73061500
C	-1.15860800	-3.33716000	-2.03633000
C	0.32115000	-3.14646400	-2.18511400
C	-0.71924200	-2.09079800	3.88267700
C	0.50483600	-1.79685600	4.45408700
C	2.63526700	-1.48597600	0.10229100
C	2.19670400	-2.15040300	-1.17460400
C	-4.79312000	-2.28293700	0.92032400
C	4.21187600	1.10503700	-0.22967300
C	-3.80802800	-0.87394000	-2.62381700
C	2.03086700	0.23619800	1.67309700
O	1.11906653	3.45668086	0.23532303
O	8.58624500	-0.23233900	-1.11133300
C	-4.49193500	-0.17536900	-0.40251300
C	-3.20648900	-2.22667400	-2.34807600
C	2.75630400	0.87917000	-0.54459300
C	-3.05186900	-3.01740600	2.36834000
C	0.31474751	2.59047591	-0.13252307
C	7.37651700	-0.49494000	-1.29481200
C	6.38610100	0.44045600	-0.58297500
C	-4.31222900	-3.20358000	1.83271400
C	4.60665300	2.07888600	0.68143200
C	-3.08240745	2.51942768	-1.54738062
C	6.85926400	1.39187600	0.31651100
C	-3.96749500	1.43292400	-2.08774300
C	-2.60963978	4.81366309	-1.08611122
C	-1.04195047	3.07157547	-0.63989931
C	5.95645700	2.22172900	0.95735000
C	-1.36613082	4.42184056	-0.62303800
C	-3.48624229	3.84767803	-1.55419184
H	7.92445300	1.46176200	0.48843300
H	6.29785800	2.97318800	1.66053700
H	3.86747200	2.71326000	1.15635700

H	2.67728100	0.58514300	-1.59359100
H	2.19691400	1.80744400	-0.40630200
H	1.67912800	1.27201800	1.71678100
H	3.01538800	0.19331300	2.15915200
H	2.48359400	-1.57017700	-2.06100000
H	2.68746800	-3.12867300	-1.24913500
H	2.39508000	-0.81765100	4.13627700
H	0.74101500	-2.14530700	5.45229600
H	-1.45818200	-2.65255700	4.43665700
H	-2.65120900	-3.72658500	3.07906900
H	-4.91193100	-4.05687700	2.12543100
H	-5.78061000	-2.38480500	0.48813700
H	-0.64214103	5.13015093	-0.24465611
H	-2.89854924	5.85819594	-1.07805039
H	-4.47191538	4.11364159	-1.91570254
H	-3.83675739	1.39369032	-3.17335946
H	-5.02016437	1.69442746	-1.90318887
H	-4.57797000	0.74564300	0.18093800
H	-5.50897800	-0.44468300	-0.72169500
H	0.81166000	-4.12725100	-2.14251600
H	0.54632300	-2.69390900	-3.16007200
H	-1.50006100	-4.04489000	-2.80149600
H	-1.39525700	-3.75598900	-1.04938600
H	-3.42867500	-2.87809100	-3.20275100
H	-3.63434000	-2.69877200	-1.45745300
H	-4.87314400	-1.01625100	-2.86631700
H	-3.32530800	-0.47204900	-3.51799200
H	3.73125100	-1.48732500	0.14086700
H	2.27897800	-2.09914600	0.93252300

Ba 0
S 5 1.00
114.075390 0.001868
52.874016 -0.008752
25.187082 0.029840
12.287174 -0.086380
5.950803 0.218765
S 1 1.00
2.807950 1.0
S 1 1.00
1.303956 1.0
S 1 1.00
0.565336 1.0
S 1 1.00
0.251764 1.0
S 1 1.00

0.104792 1.0
S 1 1.00
0.045562 1.0
S 1 1.00
0.020317 1.0
S 1 1.00
0.008330 1.0
P 4 1.00
51.225054 -0.000088
24.862306 0.000464
12.277196 -0.003409
5.958786 0.032871
P 1 1.00
2.810868 1.0
P 1 1.00
1.364893 1.0
P 1 1.00
0.642352 1.0
P 1 1.00
0.273064 1.0
P 1 1.00
0.106254 1.0
P 1 1.00
0.028185 1.0
P 1 1.00
0.010657 1.0
P 1 1.00
0.004633 1.0
D 1 1.00
1.303956 1.0
D 1 1.00
0.565336 1.0
D 1 1.00
0.251764 1.0
D 1 1.00
0.104792 1.0
D 1 1.00
0.045562 1.0
D 1 1.00
0.020317 1.0
F 1 1.00
1.364893 1.0
F 1 1.00
0.642352 1.0
F 1 1.00
0.273064 1.0

F 1 1.00
0.106254 1.0
G 1 1.00
0.565336 1.0
G 1 1.00
0.251764 1.0
****
C H N O 0
def2tzvp
****

Ba 0
ECP46MDF 5 46
H-Komponente
1
2 1. 0.
S-H
2
2 4.177931587 84.785457583
2 2.522632800 17.372709041
P-H
4
2 6.294119351 52.512225743
2 6.476457746 105.022668647
2 2.284326647 8.707014937
2 2.091555201 17.165458832
D-H
4
2 1.925291745 5.346535679
2 1.878534118 8.025720742
2 0.907088727 1.346295081
2 0.910060953 2.063710453
F-H
4
2 6.256321669 -20.003223472
2 6.134135837 -26.118214748
2 1.641382784 -2.344457989
2 1.599343316 -2.980867480
G-H
2
2 2.142381001 -3.316602759
2 2.159981109 -4.275647018

8.2 DFT optimized geometries

Δ(δλδ)-[Ba(**bpycropa**)], 0 imaginary frequencies

Center Number	Atomic Number	Coordinates (Angstroms) X	Y	Z
1	56	-0.073288	0.003959	0.052818
2	8	0.246096	-2.317850	1.819965
3	8	0.999190	0.275470	2.759134
4	7	2.217427	-1.499590	-1.185054
5	8	-0.926777	0.228448	-2.507830
6	8	-2.056931	0.751557	1.759410
7	7	2.740771	1.044645	-0.282324
8	7	-0.484299	-2.813529	-0.955421
9	7	0.806877	2.694603	1.083112
10	7	-2.647578	-1.327076	0.147573
11	6	3.465378	-1.200410	-0.815855
12	6	1.926322	-2.746107	-1.557962
13	7	-1.078474	2.459305	-1.002115
14	6	3.768892	0.223598	-0.513984
15	6	2.978557	2.332686	-0.038439
16	6	2.876890	-3.758090	-1.571222
17	6	0.573831	-0.776103	3.598922
18	6	0.988604	-2.087045	2.998286
19	6	4.460168	-2.175513	-0.752337
20	6	4.159089	-3.467441	-1.139910
21	6	-0.411367	-3.827233	0.095481
22	6	0.582206	-3.539925	1.196444
23	6	4.264903	2.857625	-0.009912
24	6	-2.896274	-2.384079	-0.622619
25	6	1.355425	2.599189	2.434471
26	6	0.514308	-3.006349	-2.001652
27	8	-2.246493	1.135545	-4.069760
28	8	-4.134792	0.762119	2.586231
29	6	1.795728	3.238995	0.165362
30	6	0.689817	1.552199	3.289025
31	6	-1.809062	-2.801851	-1.571821
32	6	5.084998	0.681082	-0.521179
33	6	-1.623106	1.155864	-2.997742
34	6	-3.233360	0.318798	1.857134
35	6	-3.584179	-0.886112	0.984647
36	6	5.332221	2.015827	-0.261885
37	6	-4.114204	-3.050761	-0.581776
38	6	-1.096932	3.558905	-0.253683
39	6	-4.831988	-1.489747	1.075234
40	6	-0.405249	3.503062	1.080845

41	6	-2.421623	4.699409	-1.880336
42	6	-1.703195	2.445046	-2.178886
43	6	-5.098146	-2.589659	0.278260
44	6	-2.396352	3.549954	-2.652970
45	6	-1.756567	4.710836	-0.665485
46	1	-5.560131	-1.090647	1.767766
47	1	-6.058219	-3.090166	0.330655
48	1	-4.281092	-3.916838	-1.210647
49	1	-1.777145	-2.071004	-2.385959
50	1	-2.061603	-3.780376	-2.007702
51	1	0.282478	-2.296881	-2.800425
52	1	0.451274	-4.020084	-2.425813
53	1	0.523937	-4.358721	1.925732
54	1	1.614265	-3.499841	0.832261
55	1	2.603497	-4.756124	-1.889493
56	1	4.917977	-4.239665	-1.103160
57	1	5.454034	-1.935950	-0.401822
58	1	5.905441	0.014826	-0.745867
59	1	6.347375	2.394053	-0.259900
60	1	4.417296	3.908137	0.203369
61	1	-2.891447	3.490037	-3.612366
62	1	-2.946184	5.583395	-2.224378
63	1	-1.742833	5.597761	-0.043668
64	1	-1.103616	3.054846	1.794335
65	1	-0.198263	4.530369	1.419784
66	1	1.314595	3.384575	-0.805851
67	1	2.153494	4.226483	0.495128
68	1	2.063599	-2.074126	2.772636
69	1	0.800998	-2.889129	3.723417
70	1	-0.516503	-0.743341	3.721628
71	1	1.036437	-0.677028	4.589340
72	1	-0.398165	1.677698	3.324093
73	1	1.081956	1.631567	4.310984
74	1	2.412925	2.337967	2.356893
75	1	1.302611	3.570188	2.950835
76	1	-1.395989	-3.894659	0.562062
77	1	-0.188696	-4.819684	-0.326009

Zero-point correction = 0.613732 Hartree; Thermal correction to Energy = 0.652600; Thermal correction to Enthalpy = 0.653545; Thermal correction to Gibbs Free Energy = 0.541822; Sum of electronic and zero-point Energies = -2042.223094; Sum of electronic and thermal Energies = -2042.184226; Sum of electronic and thermal Enthalpies = -2042.183282; Sum of electronic and thermal Free Energies = -2042.295004.

Δ(δXδ)-[Ba(**bpycropa**)], 1 imaginary frequency

Center Number	Atomic Number	Coordinates (Angstroms) X	Y	Z
1	56	-0.079790	0.048802	-0.046536
2	8	0.190172	-2.236410	1.748078
3	8	0.304444	0.054409	2.839002
4	7	2.125921	-1.775775	-1.061221
5	8	-0.723286	0.489689	-2.625876
6	8	-2.170318	1.159334	1.339504
7	7	2.902236	0.624057	0.051125
8	7	-0.711331	-2.744872	-1.017086
9	7	1.039250	2.473515	1.277976
10	7	-2.799019	-1.045567	-0.062276
11	6	3.375948	-1.658686	-0.606136
12	6	1.712432	-2.952822	-1.532616
13	7	-0.537293	2.677668	-1.054482
14	6	3.830406	-0.314277	-0.161472
15	6	3.287279	1.842259	0.429436
16	6	2.537777	-4.068131	-1.581732
17	6	-0.617887	-0.777663	3.507447
18	6	-0.681641	-2.169609	2.856323
19	6	4.243533	-2.750520	-0.569241
20	6	3.819919	-3.964854	-1.073320
21	6	-0.756166	-3.732642	0.060440
22	6	0.271650	-3.519692	1.156407
23	6	4.622260	2.173507	0.629081
24	6	-3.090416	-2.130388	-0.777061
25	6	1.347750	2.191682	2.678238
26	6	0.298018	-3.033881	-2.030802
27	8	-1.453052	1.728039	-4.339566
28	8	-4.204564	1.216157	2.268217
29	6	2.236148	2.906524	0.574823
30	6	0.313625	1.356926	3.389932
31	6	-2.008597	-2.646748	-1.681122
32	6	5.189901	-0.046517	-0.012817
33	6	-1.060808	1.572363	-3.173558
34	6	-3.338180	0.734326	1.520373
35	6	-3.727232	-0.513701	0.730425
36	6	5.586993	1.212536	0.395091
37	6	-4.338797	-2.735781	-0.720642
38	6	-0.452171	3.733043	-0.248398
39	6	-5.001189	-1.056530	0.839941
40	6	0.002423	3.491884	1.165556
41	6	-1.279652	5.169543	-1.967202

42	6	-0.981219	2.827068	-2.301541
43	6	-5.309478	-2.185549	0.101492
44	6	-1.367420	4.064605	-2.797770
45	6	-0.810718	5.006471	-0.674026
46	1	-5.716807	-0.585926	1.499901
47	1	-6.292201	-2.638035	0.167764
48	1	-4.539035	-3.624108	-1.307216
49	1	-1.896546	-1.940962	-2.510418
50	1	-2.317713	-3.612303	-2.109290
51	1	0.176585	-2.291337	-2.824875
52	1	0.134693	-4.025963	-2.478431
53	1	0.133338	-4.297243	1.916505
54	1	1.288100	-3.616340	0.778033
55	1	2.164888	-5.002171	-1.982308
56	1	4.481237	-4.822743	-1.060865
57	1	5.234333	-2.661408	-0.148113
58	1	5.934084	-0.799443	-0.227648
59	1	6.638311	1.440080	0.523709
60	1	4.890441	3.171218	0.953078
61	1	-1.722191	4.136722	-3.816657
62	1	-1.567311	6.151707	-2.324430
63	1	-0.719211	5.850852	-0.001626
64	1	-0.868549	3.147699	1.729445
65	1	0.334079	4.445439	1.605746
66	1	1.942825	3.217252	-0.432299
67	1	2.686062	3.787659	1.057876
68	1	-0.392049	-2.934601	3.585108
69	1	-1.711032	-2.380456	2.544532
70	1	-1.607221	-0.308874	3.482855
71	1	-0.319807	-0.875241	4.558361
72	1	-0.690038	1.789447	3.310544
73	1	0.580333	1.307283	4.453849
74	1	2.296210	1.650568	2.711836
75	1	1.491211	3.126424	3.243707
76	1	-1.749271	-3.683123	0.511687
77	1	-0.641989	-4.756388	-0.330744

Zero-point correction = 0.613896; Thermal correction to Energy = 0.652128; Thermal correction to Enthalpy = 0.653072; Thermal correction to Gibbs Free Energy = 0.542464; Sum of electronic and zero-point Energies = -2042.210049; Sum of electronic and thermal Energies = -2042.171817; Sum of electronic and thermal Enthalpies = -2042.170873; Sum of electronic and thermal Free Energies = -2042.281481.

Δ(δδδ)-[Ba(**bpycropa**)], 0 imaginary frequencies

Center Number	Atomic Number	Coordinates (Angstroms) X	Y	Z
1	56	-0.028585	0.003766	-0.011898
2	8	0.958009	-2.262840	1.835985
3	8	0.633507	0.241652	2.757825
4	7	2.426899	-1.372976	-1.081897
5	8	-1.036730	0.222673	-2.508190
6	8	-2.152917	0.590267	1.636093
7	7	2.697762	1.243592	-0.199431
8	7	-0.158855	-2.895323	-0.861091
9	7	0.609578	2.677644	1.160982
10	7	-2.487344	-1.563823	0.066403
11	6	3.638803	-0.922939	-0.743721
12	6	2.276688	-2.658276	-1.401550
13	7	-1.249733	2.411676	-0.945133
14	6	3.793831	0.532618	-0.483322
15	6	2.814224	2.550890	0.031347
16	6	3.338015	-3.554814	-1.397538
17	6	0.289895	-0.830925	3.608393
18	6	-0.071574	-2.034679	2.777872
19	6	4.739444	-1.774869	-0.664748
20	6	4.584696	-3.105684	-1.002837
21	6	-0.109259	-3.829441	0.261363
22	6	0.980418	-3.561603	1.282202
23	6	4.038936	3.206893	-0.008399
24	6	-2.620796	-2.635918	-0.710474
25	6	1.161799	2.545126	2.506778
26	6	0.908091	-3.108329	-1.833916
27	8	-2.320466	1.162778	-4.080239
28	8	-4.275438	0.479799	2.336058
29	6	1.562523	3.344159	0.288216
30	6	0.476547	1.508734	3.360634
31	6	-1.438901	-3.015019	-1.556377
32	6	5.050804	1.127062	-0.570797
33	6	-1.737258	1.153187	-2.985578
34	6	-3.302701	0.087662	1.670215
35	6	-3.521359	-1.145302	0.794660
36	6	5.171585	2.481867	-0.325663
37	6	-3.811892	-3.346914	-0.782835
38	6	-1.341149	3.473756	-0.148804
39	6	-4.749296	-1.792535	0.767218
40	6	-0.651845	3.405810	1.186563
41	6	-2.753724	4.588352	-1.718919

42	6	-1.884614	2.403284	-2.116619
43	6	-4.893581	-2.912072	-0.034336
44	6	-2.655658	3.476960	-2.539704
45	6	-2.082687	4.593252	-0.507447
46	1	-5.557723	-1.410007	1.374792
47	1	-5.835828	-3.446546	-0.072176
48	1	-3.883472	-4.224652	-1.413520
49	1	-1.412031	-2.328127	-2.408509
50	1	-1.588228	-4.030639	-1.953586
51	1	0.648348	-2.531534	-2.726173
52	1	0.958761	-4.165000	-2.136924
53	1	0.883402	-4.310494	2.078814
54	1	1.971054	-3.677875	0.845920
55	1	3.175028	-4.589327	-1.672401
56	1	5.427304	-3.784718	-0.953401
57	1	5.701975	-1.409760	-0.336101
58	1	5.921631	0.548926	-0.844690
59	1	6.139716	2.964541	-0.383580
60	1	4.092023	4.268029	0.200327
61	1	-3.154931	3.422044	-3.497231
62	1	-3.341431	5.447361	-2.021705
63	1	-2.128041	5.450196	0.153669
64	1	-1.325797	2.880821	1.869470
65	1	-0.512582	4.428184	1.571945
66	1	1.075788	3.517031	-0.676276
67	1	1.843712	4.333506	0.681084
68	1	-0.188722	-2.900926	3.440603
69	1	-1.029469	-1.864059	2.271280
70	1	-0.576294	-0.570441	4.227985
71	1	1.135409	-1.055000	4.270990
72	1	-0.591789	1.715742	3.490880
73	1	0.949702	1.517374	4.350578
74	1	2.210517	2.255667	2.411901
75	1	1.140144	3.509718	3.039039
76	1	-1.076196	-3.786382	0.766671
77	1	0.012859	-4.866464	-0.093725

Zero-point correction = 0.613867 Hartree; Thermal correction to Energy = 0.652762; Thermal correction to Enthalpy = 0.653706; Thermal correction to Gibbs Free Energy = 0.541585; Sum of electronic and zero-point Energies = -2042.215806; Sum of electronic and thermal Energies = -2042.176911; Sum of electronic and thermal Enthalpies = -2042.175967; Sum of electronic and thermal Free Energies = -2042.288088.

Δ(Xδδ)-[Ba(**bpycropa**)], 1 imaginary frequency

Center Number	Atomic Number	Coordinates (Angstroms) X	Y	Z
1	56	-0.051667	-0.006175	-0.016893
2	8	0.903457	-2.309644	1.466722
3	8	1.756366	0.309290	2.189826
4	7	2.267389	-1.284432	-1.365093
5	8	-1.270722	0.280879	-2.422352
6	8	-1.823759	0.380082	2.025462
7	7	2.555709	1.341142	-0.554279
8	7	-0.330618	-2.888283	-0.966801
9	7	0.659912	2.734709	1.068610
10	7	-2.465401	-1.549851	0.263553
11	6	3.466723	-0.845577	-0.982029
12	6	2.064352	-2.593709	-1.516463
13	7	-1.486499	2.366338	-0.733023
14	6	3.642232	0.622816	-0.843152
15	6	2.674317	2.650432	-0.346976
16	6	3.057957	-3.529146	-1.265798
17	6	2.253659	-0.859507	2.801328
18	6	1.270195	-1.999250	2.800900
19	6	4.512417	-1.722906	-0.700700
20	6	4.297734	-3.081721	-0.838981
21	6	-0.241289	-3.960456	0.025411
22	6	0.444619	-3.641081	1.367180
23	6	3.892675	3.308442	-0.469207
24	6	-2.736269	-2.524454	-0.601074
25	6	1.405519	2.636620	2.327137
26	6	0.698027	-2.994920	-1.997629
27	8	-2.958010	1.053257	-3.671757
28	8	-3.868537	0.302443	2.929916
29	6	1.430894	3.409580	0.032800
30	6	1.210969	1.313538	3.030382
31	6	-1.653832	-2.868325	-1.586534
32	6	4.895270	1.209908	-0.995282
33	6	-2.169742	1.111657	-2.715853
34	6	-2.997350	-0.062539	2.125118
35	6	-3.384965	-1.172720	1.148327
36	6	5.014377	2.576223	-0.816256
37	6	-3.966332	-3.169411	-0.613397
38	6	-1.527441	3.408617	0.091641
39	6	-4.643042	-1.759353	1.192628
40	6	-0.623252	3.385106	1.293631
41	6	-3.254158	4.426209	-1.207088

42	6	-2.293598	2.323841	-1.792459
43	6	-4.935072	-2.772465	0.295098
44	6	-3.204474	3.335743	-2.060179
45	6	-2.397915	4.471844	-0.119564
46	1	-5.358538	-1.416702	1.927307
47	1	-5.904538	-3.257225	0.307311
48	1	-4.154604	-3.969368	-1.319190
49	1	-1.645619	-2.090104	-2.356642
50	1	-1.894083	-3.824315	-2.075704
51	1	0.424821	-2.314927	-2.807414
52	1	0.738579	-4.012492	-2.414211
53	1	-0.270149	-3.830501	2.177018
54	1	1.296147	-4.315256	1.520687
55	1	2.858978	-4.585293	-1.399312
56	1	5.090534	-3.785565	-0.615772
57	1	5.468851	-1.351291	-0.358423
58	1	5.757110	0.613773	-1.263494
59	1	5.975427	3.062323	-0.933960
60	1	3.954331	4.374452	-0.288862
61	1	-3.844145	3.253183	-2.928126
62	1	-3.946355	5.239564	-1.392219
63	1	-2.398698	5.317514	0.557492
64	1	-1.137509	2.825029	2.082120
65	1	-0.489970	4.416429	1.656919
66	1	0.797946	3.499847	-0.854497
67	1	1.711798	4.430965	0.331242
68	1	1.756372	-2.863087	3.271113
69	1	0.370418	-1.757545	3.381466
70	1	2.567305	-0.656388	3.831177
71	1	3.136700	-1.156100	2.227554
72	1	0.150974	1.114985	3.225649
73	1	1.746807	1.333918	3.986018
74	1	2.470912	2.739882	2.112957
75	1	1.136346	3.455381	3.008667
76	1	-1.257704	-4.277948	0.257688
77	1	0.251850	-4.833357	-0.419595

Zero-point correction= 0.613333 Hartree; Thermal correction to Energy= 0.651737; thermal correction to Enthalpy= 0.652681; thermal correction to Gibbs Free Energy= 0.541445; sum of electronic and zero-point Energies= -2042.205191; Sum of electronic and thermal Energies= -2042.166788; Sum of electronic and thermal Enthalpies= -2042.165843; Sum of electronic and thermal Free Energies= -2042.277079.

Δ(λδδ)-[Ba(**bpycropa**)], 0 imaginary frequencies

Center Number	Atomic Number	Coordinates (Angstroms) X	Y	Z
1	56	-0.006900	-0.010128	0.032813
2	8	-0.353290	-2.313200	1.850464
3	8	0.929652	0.021639	2.789969
4	7	2.036253	-1.837142	-1.151759
5	8	-0.682804	0.361512	-2.558502
6	8	-1.942397	1.139820	1.593424
7	7	2.935571	0.565737	-0.151735
8	7	-0.843452	-2.723596	-0.990979
9	7	1.225244	2.480812	1.165429
10	7	-2.802897	-0.846954	-0.008725
11	6	3.298607	-1.755594	-0.724720
12	6	1.549480	-3.017499	-1.536863
13	7	-0.533664	2.581667	-1.034036
14	6	3.822372	-0.407174	-0.381004
15	6	3.372925	1.788450	0.145312
16	6	2.308787	-4.179281	-1.498276
17	6	0.538676	-1.041127	3.641466
18	6	-0.666527	-1.779425	3.122333
19	6	4.109410	-2.885636	-0.623800
20	6	3.605035	-4.109963	-1.017633
21	6	-0.862654	-3.763396	0.037397
22	6	-1.273534	-3.292089	1.412812
23	6	4.726488	2.091145	0.233557
24	6	-3.179152	-1.909738	-0.717521
25	6	1.654367	2.270574	2.547225
26	6	0.132116	-3.034298	-2.036216
27	8	-1.703875	1.496533	-4.193536
28	8	-4.040902	1.607924	2.211492
29	6	2.350912	2.875396	0.332820
30	6	0.777405	1.325324	3.330102
31	6	-2.145769	-2.523281	-1.618083
32	6	5.195040	-0.173482	-0.336002
33	6	-1.166703	1.400378	-3.079721
34	6	-3.184408	0.947808	1.602247
35	6	-3.684293	-0.239336	0.782228
36	6	5.649007	1.093191	-0.019915
37	6	-4.473235	-2.409969	-0.660736
38	6	-0.417203	3.663128	-0.268051
39	6	-5.001680	-0.668905	0.883969
40	6	0.168667	3.482596	1.105480
41	6	-1.430587	5.019906	-1.951862

42	6	-1.078618	2.679647	-2.245782
43	6	-5.398888	-1.773802	0.151362
44	6	-1.548339	3.887888	-2.741515
45	6	-0.850334	4.911603	-0.698870
46	1	-5.679334	-0.136213	1.536943
47	1	-6.415581	-2.143815	0.217207
48	1	-4.742954	-3.285920	-1.237946
49	1	-1.993269	-1.832644	-2.453436
50	1	-2.536442	-3.466268	-2.029803
51	1	0.033084	-2.266336	-2.807327
52	1	-0.075683	-4.008380	-2.504158
53	1	-2.290517	-2.889427	1.428530
54	1	-1.248577	-4.153850	2.092864
55	1	1.880747	-5.118931	-1.824453
56	1	4.216297	-5.001539	-0.946879
57	1	5.114384	-2.816727	-0.232094
58	1	5.901386	-0.959467	-0.562175
59	1	6.711832	1.298210	0.024970
60	1	5.041913	3.094434	0.491129
61	1	-1.986582	3.917776	-3.729557
62	1	-1.778861	5.981326	-2.311573
63	1	-0.729025	5.779328	-0.061786
64	1	-0.639700	3.151663	1.764254
65	1	0.524923	4.457240	1.474792
66	1	1.964985	3.140497	-0.655683
67	1	2.850773	3.772587	0.728914
68	1	-0.909383	-2.587481	3.824588
69	1	-1.533436	-1.111495	3.045080
70	1	0.321072	-0.670526	4.647581
71	1	1.382233	-1.735388	3.709618
72	1	-0.276635	1.620872	3.295956
73	1	1.111329	1.334196	4.373705
74	1	2.660427	1.845843	2.531060
75	1	1.718105	3.228204	3.087124
76	1	-1.513925	-4.600178	-0.257629
77	1	0.148120	-4.166149	0.128327

Zero-point correction= 0.613797 Hartree; Thermal correction to Energy= 0.652717; Thermal correction to Enthalpy= 0.653661; Thermal correction to Gibbs Free Energy= 0.541378; Sum of electronic and zero-point Energies= -2042.218121; Sum of electronic and thermal Energies= -2042.179201; Sum of electronic and thermal Enthalpies= -2042.178257; Sum of electronic and thermal Free Energies= -2042.290540

Δ(λδX)-[Ba(**bpycropa**)], 1 imaginary frequency

Center Number	Atomic Number	Coordinates (Angstroms) X	Y	Z
1	56	-0.003072	-0.018205	0.018333
2	8	-0.356357	-2.151575	1.960357
3	8	0.562334	0.407292	2.807732
4	7	2.183202	-1.852743	-1.087290
5	8	-0.760196	0.414128	-2.543581
6	8	-2.120108	1.107562	1.363849
7	7	2.954148	0.672802	-0.272643
8	7	-0.648327	-2.873278	-0.856192
9	7	1.283599	2.505874	1.122853
10	7	-2.766975	-1.050772	-0.108589
11	6	3.441071	-1.652593	-0.689534
12	6	1.770080	-3.096699	-1.330909
13	7	-0.658743	2.564017	-0.931575
14	6	3.887670	-0.246951	-0.526004
15	6	3.319590	1.946668	-0.139670
16	6	2.594672	-4.200349	-1.157432
17	6	0.284584	-0.654665	3.698347
18	6	-0.798113	-1.542361	3.158094
19	6	4.320303	-2.710715	-0.465485
20	6	3.886079	-4.001127	-0.699863
21	6	-0.638688	-3.805584	0.270917
22	6	-1.161545	-3.247598	1.573000
23	6	4.642307	2.356079	-0.259508
24	6	-3.044755	-2.192177	-0.735411
25	6	1.946554	2.415634	2.426105
26	6	0.365803	-3.251009	-1.840145
27	8	-1.944913	1.548598	-4.066101
28	8	-4.278546	1.519557	1.787746
29	6	2.235356	2.955312	0.115296
30	6	1.588541	1.221566	3.328754
31	6	-1.938625	-2.817426	-1.534848
32	6	5.231932	0.085729	-0.673239
33	6	-1.352391	1.434903	-2.982084
34	6	-3.346614	0.852861	1.306039
35	6	-3.727473	-0.431681	0.574872
36	6	5.610278	1.408555	-0.538043
37	6	-4.308089	-2.767349	-0.696280
38	6	-0.607002	3.592061	-0.091018
39	6	-5.021990	-0.931235	0.647973
40	6	0.137928	3.407060	1.203195
41	6	-1.970682	4.902512	-1.548838

42	6	-1.340018	2.663509	-2.071216
43	6	-5.313406	-2.121411	0.005433
44	6	-2.017803	3.823866	-2.417275
45	6	-1.249709	4.792256	-0.371209
46	1	-5.764444	-0.382711	1.211164
47	1	-6.308701	-2.547846	0.055154
48	1	-4.492897	-3.706847	-1.202622
49	1	-1.802324	-2.204505	-2.431636
50	1	-2.253566	-3.818662	-1.866496
51	1	0.246626	-2.591430	-2.703231
52	1	0.217798	-4.283359	-2.191674
53	1	-2.208873	-2.940439	1.504430
54	1	-1.097839	-4.037269	2.333203
55	1	2.221469	-5.194822	-1.367920
56	1	4.547321	-4.841926	-0.528371
57	1	5.322096	-2.529079	-0.101104
58	1	5.966551	-0.670656	-0.912723
59	1	6.648910	1.695709	-0.649850
60	1	4.899579	3.400558	-0.135449
61	1	-2.559508	3.860957	-3.352353
62	1	-2.484598	5.826024	-1.789753
63	1	-1.182235	5.621830	0.322249
64	1	-0.564265	2.983041	1.928005
65	1	0.442473	4.397770	1.575876
66	1	1.695731	3.111818	-0.821695
67	1	2.693790	3.918330	0.386841
68	1	-1.014901	-2.307424	3.913736
69	1	-1.712706	-0.967824	2.970133
70	1	-0.042508	-0.254946	4.667258
71	1	1.197381	-1.242679	3.865879
72	1	1.281983	1.589184	4.315945
73	1	2.483622	0.604280	3.479867
74	1	3.021067	2.364117	2.249776
75	1	1.779446	3.344467	2.985280
76	1	-1.201740	-4.720899	0.031335
77	1	0.396175	-4.106912	0.446037

Zero-point correction= 0.613700 Hartree; Thermal correction to Energy= 0.652022; Thermal correction to Enthalpy= 0.652966; Thermal correction to Gibbs Free Energy= 0.541881; Sum of electronic and zero-point Energies= -2042.205456; Sum of electronic and thermal Energies= -2042.167133; Sum of electronic and thermal Enthalpies= -2042.166189; Sum of electronic and thermal Free Energies= -2042.277274.

Δ(λδλ)-[Ba(**bpycropa**)], 0 imaginary frequencies

Center Number	Atomic Number	Coordinates (Angstroms) X	Y	Z
1	56	-0.006900	-0.010128	0.032813
2	8	-0.353290	-2.313200	1.850464
3	8	0.929652	0.021639	2.789969
4	7	2.036253	-1.837142	-1.151759
5	8	-0.682804	0.361512	-2.558502
6	8	-1.942397	1.139820	1.593424
7	7	2.935571	0.565737	-0.151735
8	7	-0.843452	-2.723596	-0.990979
9	7	1.225244	2.480812	1.165429
10	7	-2.802897	-0.846954	-0.008725
11	6	3.298607	-1.755594	-0.724720
12	6	1.549480	-3.017499	-1.536863
13	7	-0.533664	2.581667	-1.034036
14	6	3.822372	-0.407174	-0.381004
15	6	3.372925	1.788450	0.145312
16	6	2.308787	-4.179281	-1.498276
17	6	0.538676	-1.041127	3.641466
18	6	-0.666527	-1.779425	3.122333
19	6	4.109410	-2.885636	-0.623800
20	6	3.605035	-4.109963	-1.017633
21	6	-0.862654	-3.763396	0.037397
22	6	-1.273534	-3.292089	1.412812
23	6	4.726488	2.091145	0.233557
24	6	-3.179152	-1.909738	-0.717521
25	6	1.654367	2.270574	2.547225
26	6	0.132116	-3.034298	-2.036216
27	8	-1.703875	1.496533	-4.193536
28	8	-4.040902	1.607924	2.211492
29	6	2.350912	2.875396	0.332820
30	6	0.777405	1.325324	3.330102
31	6	-2.145769	-2.523281	-1.618083
32	6	5.195040	-0.173482	-0.336002
33	6	-1.166703	1.400378	-3.079721
34	6	-3.184408	0.947808	1.602247
35	6	-3.684293	-0.239336	0.782228
36	6	5.649007	1.093191	-0.019915
37	6	-4.473235	-2.409969	-0.660736
38	6	-0.417203	3.663128	-0.268051
39	6	-5.001680	-0.668905	0.883969
40	6	0.168667	3.482596	1.105480
41	6	-1.430587	5.019906	-1.951862

42	6	-1.078618	2.679647	-2.245782
43	6	-5.398888	-1.773802	0.151362
44	6	-1.548339	3.887888	-2.741515
45	6	-0.850334	4.911603	-0.698870
46	1	-5.679334	-0.136213	1.536943
47	1	-6.415581	-2.143815	0.217207
48	1	-4.742954	-3.285920	-1.237946
49	1	-1.993269	-1.832644	-2.453436
50	1	-2.536442	-3.466268	-2.029803
51	1	0.033084	-2.266336	-2.807327
52	1	-0.075683	-4.008380	-2.504158
53	1	-2.290517	-2.889427	1.428530
54	1	-1.248577	-4.153850	2.092864
55	1	1.880747	-5.118931	-1.824453
56	1	4.216297	-5.001539	-0.946879
57	1	5.114384	-2.816727	-0.232094
58	1	5.901386	-0.959467	-0.562175
59	1	6.711832	1.298210	0.024970
60	1	5.041913	3.094434	0.491129
61	1	-1.986582	3.917776	-3.729557
62	1	-1.778861	5.981326	-2.311573
63	1	-0.729025	5.779328	-0.061786
64	1	-0.639700	3.151663	1.764254
65	1	0.524923	4.457240	1.474792
66	1	1.964985	3.140497	-0.655683
67	1	2.850773	3.772587	0.728914
68	1	-0.909383	-2.587481	3.824588
69	1	-1.533436	-1.111495	3.045080
70	1	0.321072	-0.670526	4.647581
71	1	1.382233	-1.735388	3.709618
72	1	-0.276635	1.620872	3.295956
73	1	1.111329	1.334196	4.373705
74	1	2.660427	1.845843	2.531060
75	1	1.718105	3.228204	3.087124
76	1	-1.513925	-4.600178	-0.257629
77	1	0.148120	-4.166149	0.128327

Zero-point correction= 0.613797 Hartree; Thermal correction to Energy= 0.652717; Thermal correction to Enthalpy= 0.653661; Thermal correction to Gibbs Free Energy= 0.541378; Sum of electronic and zero-point Energies= -2042.218121; Sum of electronic and thermal Energies= -2042.179201; Sum of electronic and thermal Enthalpies= -2042.178257; Sum of electronic and thermal Free Energies= -2042.290540.

(λδλ)-[Ba(**bpycropa**)], arm dissociation, 1 imaginary frequency

Center Number	Atomic Number	Coordinates (Angstroms) X	Y	Z
1	56	0.354137	-0.464411	-0.217465
2	8	-1.332342	1.709176	-1.318636
3	8	0.715709	0.592778	-2.907015
4	7	0.620134	1.890770	1.463449
5	8	0.367393	-1.520226	2.228084
6	8	-4.904476	-1.822858	-2.741938
7	7	2.766178	1.275717	-0.197825
8	7	-1.972579	0.646502	1.345995
9	7	2.804637	-1.183245	-1.871710
10	7	-4.439796	-1.004117	-0.158068
11	6	1.608657	2.774852	1.304836
12	6	-0.275927	2.082635	2.432597
13	7	2.270287	-2.498810	0.607578
14	6	2.590798	2.535454	0.218109
15	6	3.716822	1.020061	-1.097455
16	6	-0.207550	3.169250	3.296339
17	6	0.117072	1.840148	-3.197445
18	6	-1.304732	1.826345	-2.723704
19	6	1.752446	3.876597	2.144300
20	6	0.830003	4.071607	3.155042
21	6	-2.626710	1.771886	0.683921
22	6	-2.658651	1.670050	-0.819420
23	6	4.513062	2.020122	-1.642726
24	6	-4.215984	-0.523846	1.064105
25	6	2.475385	-0.965424	-3.278614
26	6	-1.361016	1.057834	2.604057
27	8	1.273910	-2.538172	4.000484
28	8	-7.108862	-1.601640	-2.421525
29	6	3.973853	-0.418690	-1.450108
30	6	1.930128	0.399945	-3.601119
31	6	-2.796990	-0.573388	1.556947
32	6	3.322929	3.594576	-0.312515
33	6	1.226205	-2.245050	2.798451
34	6	-5.911068	-1.524518	-2.069731
35	6	-5.678070	-0.988127	-0.648953
36	6	4.295377	3.329763	-1.258151
37	6	-5.235075	-0.011888	1.860060
38	6	3.195464	-2.978932	-0.220915
39	6	-6.750778	-0.489768	0.084700
40	6	3.053653	-2.609688	-1.669352
41	6	4.272099	-4.144470	1.562362

42	6	2.317018	-2.819511	1.898999
43	6	-6.525086	0.005844	1.356708
44	6	3.305892	-3.645173	2.417856
45	6	4.220395	-3.805143	0.219464
46	1	-7.736675	-0.506933	-0.358744
47	1	-7.342219	0.397739	1.951798
48	1	-5.016264	0.361947	2.853372
49	1	-2.321566	-1.431077	1.073899
50	1	-2.817148	-0.789855	2.627597
51	1	-0.904626	0.168467	3.047994
52	1	-2.105732	1.448297	3.313454
53	1	-3.152965	0.751397	-1.152299
54	1	-3.223810	2.524482	-1.213972
55	1	-0.959812	3.296050	4.064612
56	1	0.919956	4.919921	3.822773
57	1	2.582912	4.558209	2.025657
58	1	3.128144	4.611230	-0.000981
59	1	4.875764	4.136526	-1.689073
60	1	5.273830	1.768735	-2.370974
61	1	3.297604	-3.873842	3.474660
62	1	5.061750	-4.786453	1.935057
63	1	4.963614	-4.168632	-0.479492
64	1	2.199610	-3.156456	-2.080365
65	1	3.943075	-2.945582	-2.222848
66	1	4.369823	-0.890773	-0.546426
67	1	4.769124	-0.469698	-2.207606
68	1	-1.793868	2.758533	-3.034789
69	1	-1.843921	0.988327	-3.185538
70	1	0.123327	2.017150	-4.279654
71	1	0.679751	2.648909	-2.713057
72	1	1.749996	0.449594	-4.682421
73	1	2.637865	1.197355	-3.351105
74	1	3.354146	-1.140821	-3.919672
75	1	1.719577	-1.703882	-3.557698
76	1	-3.649882	1.935279	1.044865
77	1	-2.063443	2.672576	0.935598

Zero-point correction= 0.613528 Hartree; Thermal correction to Energy= 0.651943; Thermal correction to Enthalpy= 0.652887; Thermal correction to Gibbs Free Energy= 0.541176; Sum of electronic and zero-point Energies= -2042.190675; Sum of electronic and thermal Energies= -2042.152260; Sum of electronic and thermal Enthalpies= -2042.151315; Sum of electronic and thermal Free Energies= -2042.263027.

(λδλ)-[Ba(**bpycropa**)], arm dissociation, 0 imaginary frequencies

Center Number	Atomic Number	Coordinates (Angstroms) X	Y	Z
1	56	-0.711178	0.182841	-0.726853
2	8	0.791794	-2.322028	-1.142067
3	8	-1.808017	-2.092936	-2.202790
4	7	-0.113359	-0.888378	1.913818
5	8	-0.037723	2.374456	0.631092
6	8	6.899359	-1.422915	-1.975854
7	7	-2.750089	-1.056900	1.040884
8	7	2.086317	-0.143008	0.270468
9	7	-3.643736	0.109977	-1.555384
10	7	5.086668	0.309796	-0.847279
11	6	-0.991704	-1.626171	2.598728
12	6	1.071034	-0.611694	2.460575
13	7	-2.446070	2.514586	-0.557113
14	6	-2.304334	-1.912831	1.967429
15	6	-3.974182	-1.227029	0.539248
16	6	1.419691	-1.055192	3.730615
17	6	-1.158608	-3.337160	-2.036330
18	6	0.321150	-3.146464	-2.185114
19	6	-0.719242	-2.090798	3.882677
20	6	0.504836	-1.796856	4.454087
21	6	2.635267	-1.485976	0.102291
22	6	2.196704	-2.150403	-1.174604
23	6	-4.793120	-2.282937	0.920324
24	6	4.211876	1.105037	-0.229673
25	6	-3.808028	-0.873940	-2.623817
26	6	2.030867	0.236198	1.673097
27	8	-0.351984	4.353173	1.622454
28	8	8.586245	-0.232339	-1.111333
29	6	-4.491935	-0.175369	-0.402513
30	6	-3.206489	-2.226674	-2.348076
31	6	2.756304	0.879170	-0.544593
32	6	-3.051869	-3.017406	2.368340
33	6	-0.703606	3.409684	0.902217
34	6	7.376517	-0.494940	-1.294812
35	6	6.386101	0.440456	-0.582975
36	6	-4.312229	-3.203580	1.832714
37	6	4.606653	2.078886	0.681432
38	6	-3.646141	2.547812	-1.134004
39	6	6.859264	1.391876	0.316511
40	6	-3.967495	1.432924	-2.087743
41	6	-4.180060	4.603221	-0.043124

42	6	-2.089041	3.497539	0.267892
43	6	5.956457	2.221729	0.957350
44	6	-2.929835	4.566644	0.548379
45	6	-4.548564	3.575885	-0.897199
46	1	7.924453	1.461762	0.488433
47	1	6.297858	2.973188	1.660537
48	1	3.867472	2.713260	1.156357
49	1	2.677281	0.585143	-1.593591
50	1	2.196914	1.807444	-0.406302
51	1	1.679128	1.272018	1.716781
52	1	3.015388	0.193313	2.159152
53	1	2.483594	-1.570177	-2.061000
54	1	2.687468	-3.128673	-1.249135
55	1	2.395080	-0.817651	4.136277
56	1	0.741015	-2.145307	5.452296
57	1	-1.458182	-2.652557	4.436657
58	1	-2.651209	-3.726585	3.079069
59	1	-4.911931	-4.056877	2.125431
60	1	-5.780610	-2.384805	0.488137
61	1	-2.588711	5.338644	1.224166
62	1	-4.864782	5.417992	0.161538
63	1	-5.522072	3.563840	-1.371662
64	1	-3.374628	1.578231	-2.995812
65	1	-5.025005	1.499767	-2.383989
66	1	-4.577970	0.745643	0.180938
67	1	-5.508978	-0.444683	-0.721695
68	1	0.811660	-4.127251	-2.142516
69	1	0.546323	-2.693909	-3.160072
70	1	-1.500061	-4.044890	-2.801496
71	1	-1.395257	-3.755989	-1.049386
72	1	-3.428675	-2.878091	-3.202751
73	1	-3.634340	-2.698772	-1.457453
74	1	-4.873144	-1.016251	-2.866317
75	1	-3.325308	-0.472049	-3.517992
76	1	3.731251	-1.487325	0.140867
77	1	2.278978	-2.099146	0.932523

Zero-point correction= 0.613896 Hartree; Thermal correction to Energy= 0.653040; Thermal correction to Enthalpy= 0.653985; Thermal correction to Gibbs Free Energy= 0.539408; Sum of electronic and zero-point Energies= -2042.199246; Sum of electronic and thermal Energies= -2042.160101; Sum of electronic and thermal Enthalpies= -2042.159156; Sum of electronic and thermal Free Energies= -2042.273733.

(λδλ)-[Ba(**bpycropa**)], coordinated picolinate flip, 1 imaginary frequency

Center Number	Atomic Number	Coordinates (Angstroms) X	Y	Z
1	56	0.899760	0.344812	0.314984
2	8	-0.411683	-1.812390	1.870083
3	8	2.235169	-1.033673	2.457705
4	7	0.057165	-1.696005	-1.548839
5	8	-0.148774	2.063132	-1.415201
6	8	-6.757641	-1.056192	2.382698
7	7	2.762599	-1.544362	-0.952865
8	7	-1.987097	-0.371196	-0.092630
9	7	3.942139	0.703851	0.707151
10	7	-4.977786	0.345265	0.825806
11	6	0.875752	-2.672134	-1.948097
12	6	-1.182656	-1.641879	-2.034626
13	7	2.250200	2.786043	-0.390461
14	6	2.241400	-2.704073	-1.369609
15	6	4.022179	-1.519330	-0.517242
16	6	-1.645673	-2.555642	-2.973285
17	6	1.687445	-2.265660	2.887571
18	6	0.211803	-2.107543	3.100381
19	6	0.486057	-3.618721	-2.891077
20	6	-0.792177	-3.550313	-3.414442
21	6	-2.428075	-1.564202	0.627385
22	6	-1.816184	-1.680877	1.997737
23	6	4.801098	-2.668258	-0.440601
24	6	-4.120203	0.959332	0.008958
25	6	4.169140	0.331312	2.108118
26	6	-2.080043	-0.543704	-1.535146
27	8	-0.577701	4.173919	-2.011117
28	8	-8.467010	-0.153591	1.253585
29	6	4.641666	-0.177056	-0.229359
30	6	3.644295	-1.010345	2.543162
31	6	-2.655994	0.860867	0.348198
32	6	2.952288	-3.898665	-1.300375
33	6	0.111145	3.296129	-1.475593
34	6	-7.252467	-0.345691	1.486811
35	6	-6.279979	0.379492	0.544409
36	6	4.248301	-3.877358	-0.820029
37	6	-4.536186	1.645812	-1.127233
38	6	3.399028	3.136718	0.176292
39	6	-6.773688	1.047254	-0.572855
40	6	4.418598	2.081674	0.522330
41	6	2.872079	5.459337	-0.038377

42	6	1.412046	3.741367	-0.812187
43	6	-5.888803	1.689046	-1.420780
44	6	1.691569	5.089646	-0.663828
45	6	3.739478	4.471666	0.386035
46	1	-7.840819	1.050828	-0.746501
47	1	-6.245749	2.218171	-2.297366
48	1	-3.807960	2.138142	-1.761337
49	1	-2.540010	0.938248	1.431121
50	1	-2.119241	1.693408	-0.112282
51	1	-1.771102	0.400763	-1.995768
52	1	-3.105569	-0.753326	-1.869044
53	1	-2.052503	-0.813974	2.628375
54	1	-2.227004	-2.569128	2.493599
55	1	-2.661304	-2.484433	-3.341619
56	1	-1.119964	-4.271001	-4.153867
57	1	1.174685	-4.381626	-3.226866
58	1	2.495659	-4.830789	-1.603157
59	1	4.821227	-4.793712	-0.746412
60	1	5.818994	-2.608520	-0.076379
61	1	0.980444	5.816675	-1.030684
62	1	3.113395	6.504891	0.114370
63	1	4.675978	4.721701	0.870585
64	1	4.959622	2.414511	1.412986
65	1	5.164535	2.102603	-0.279439
66	1	4.674502	0.340606	-1.191129
67	1	5.684190	-0.333301	0.084605
68	1	-0.188767	-3.041991	3.513826
69	1	0.021113	-1.304713	3.825123
70	1	2.146858	-2.568773	3.835951
71	1	1.889670	-3.045551	2.142118
72	1	3.951176	-1.164639	3.585389
73	1	4.070418	-1.829903	1.955439
74	1	5.246620	0.344613	2.338036
75	1	3.696775	1.096767	2.729118
76	1	-3.519579	-1.600026	0.724834
77	1	-2.121674	-2.439996	0.052246

Zero-point correction= 0.613529 Hartree; Thermal correction to Energy= 0.652002; Thermal correction to Enthalpy= 0.652946; Thermal correction to Gibbs Free Energy= 0.540018; Sum of electronic and zero-point Energies= -2042.188522; Sum of electronic and thermal Energies= -2042.150050; Sum of electronic and thermal Enthalpies= -2042.149105; Sum of electronic and thermal Free Energies= -2042.262033.

(λδλ)-[Ba(**bpycropa**)], coordinated picolinate flip, 0 imaginary frequencies

Center Number	Atomic Number	Coordinates (Angstroms) X	Y	Z
1	56	0.853704	0.173685	-0.527493
2	8	-0.365061	-0.203693	2.029191
3	8	2.380000	0.498285	1.894058
4	7	-0.006504	-2.688153	-0.550070
5	8	-0.374894	2.425803	0.205244
6	8	-6.483384	0.883434	2.286811
7	7	2.707998	-2.216741	-0.590199
8	7	-2.031614	-0.644960	-0.336505
9	7	3.880687	0.541292	-0.608759
10	7	-4.939924	0.674099	0.008694
11	6	0.826406	-3.683837	-0.233687
12	6	-1.293432	-2.960651	-0.762946
13	7	2.138607	2.777712	-0.688961
14	6	2.263901	-3.355609	-0.051962
15	6	4.010413	-1.936855	-0.539244
16	6	-1.806891	-4.247290	-0.658864
17	6	1.738501	0.083214	3.084135
18	6	0.301884	0.509970	3.049328
19	6	0.387550	-5.001506	-0.125414
20	6	-0.948607	-5.282689	-0.338333
21	6	-2.413487	-0.909786	1.050188
22	6	-1.764272	0.032665	2.027231
23	6	4.930508	-2.782566	0.064645
24	6	-4.204401	0.394689	-1.069181
25	6	4.430776	0.823796	0.718512
26	6	-2.194916	-1.826216	-1.167977
27	8	-0.632995	4.626654	0.500424
28	8	-8.314169	1.038729	1.006289
29	6	4.451162	-0.663591	-1.203081
30	6	3.742044	0.117208	1.860215
31	6	-2.710912	0.521650	-0.919422
32	6	3.119364	-4.229855	0.617033
33	6	0.017247	3.622640	0.178884
34	6	-7.090911	0.858440	1.198921
35	6	-6.265256	0.556727	-0.059341
36	6	4.468865	-3.937697	0.670935
37	6	-4.773739	-0.017538	-2.269275
38	6	3.404824	2.917233	-1.076055
39	6	-6.909588	0.162977	-1.229124
40	6	4.120748	1.668345	-1.508682
41	6	3.320236	5.273436	-0.703002

42	6	1.451351	3.847895	-0.294302
43	6	-6.151910	-0.133875	-2.347679
44	6	2.007486	5.120472	-0.292736
45	6	4.036508	4.153407	-1.094400
46	1	-7.989194	0.101180	-1.235569
47	1	-6.626250	-0.448699	-3.270522
48	1	-4.145025	-0.235591	-3.124760
49	1	-2.478757	1.388029	-0.300364
50	1	-2.263390	0.695098	-1.901822
51	1	-1.950771	-1.546258	-2.199119
52	1	-3.231276	-2.191283	-1.179517
53	1	-1.956722	1.081605	1.776611
54	1	-2.167372	-0.161651	3.028575
55	1	-2.861203	-4.424814	-0.829338
56	1	-1.313542	-6.299596	-0.259417
57	1	1.079397	-5.801065	0.097717
58	1	2.739647	-5.122158	1.094336
59	1	5.152523	-4.604209	1.182581
60	1	5.981184	-2.521936	0.077571
61	1	1.405072	5.956029	0.036067
62	1	3.787656	6.251275	-0.706369
63	1	5.072284	4.230126	-1.401760
64	1	5.194619	1.881625	-1.614718
65	1	3.754548	1.384037	-2.500597
66	1	4.123591	-0.703687	-2.245471
67	1	5.550239	-0.618644	-1.207650
68	1	-0.155378	0.290085	4.021947
69	1	0.225179	1.589130	2.865604
70	1	2.229681	0.540690	3.951607
71	1	1.804510	-1.007979	3.186355
72	1	4.234353	0.423663	2.791811
73	1	3.820013	-0.972720	1.791886
74	1	5.504708	0.587482	0.758250
75	1	4.334703	1.895503	0.900403
76	1	-3.500170	-0.866196	1.187649
77	1	-2.091740	-1.923046	1.300172

Zero-point correction= 0.613919 Hartree; Thermal correction to Energy= 0.653148; Thermal correction to Enthalpy= 0.654092; Thermal correction to Gibbs Free Energy= 0.539042; Sum of electronic and zero-point Energies= -2042.196941; Sum of electronic and thermal Energies= -2042.157712; Sum of electronic and thermal Enthalpies= -2042.156768; Sum of electronic and thermal Free Energies= -2042.271819

(λδλ)-[Ba(**bpycropa**)], dissociated arm rotation, 1 imaginary frequency

Center Number	Atomic Number	Coordinates (Angstroms) X	Y	Z
1	56	0.657851	0.235897	-0.311428
2	8	0.367696	-0.744069	2.388499
3	8	2.835905	0.422649	1.581227
4	7	0.252708	-2.705971	-0.471641
5	8	-0.556015	2.097583	1.158505
6	8	-6.277973	2.060586	-2.483075
7	7	2.720010	-1.760754	-1.209238
8	7	-1.947833	-1.108681	0.627501
9	7	3.428315	1.137433	-1.169457
10	7	-4.988177	0.526997	-0.577694
11	6	1.276345	-3.563564	-0.512191
12	6	-0.985240	-3.176126	-0.322034
13	7	1.479584	3.011562	-0.343448
14	6	2.636150	-3.010915	-0.746146
15	6	3.911995	-1.267135	-1.544340
16	6	-1.253300	-4.532650	-0.192162
17	6	2.672601	-0.317976	2.774311
18	6	1.275503	-0.135630	3.283751
19	6	1.083004	-4.939318	-0.401273
20	6	-0.198842	-5.426269	-0.234212
21	6	-1.844296	-1.556052	2.014054
22	6	-0.967846	-0.667836	2.857505
23	6	5.082875	-2.002709	-1.422385
24	6	-4.359164	-0.313467	0.244143
25	6	4.324463	1.354302	-0.033044
26	6	-2.119095	-2.188226	-0.332333
27	8	-0.971520	4.151535	1.937982
28	8	-8.247867	1.412776	-1.639350
29	6	3.934742	0.130345	-2.096282
30	6	4.165383	0.373829	1.102598
31	6	-2.881295	-0.013876	0.405627
32	6	3.774823	-3.788235	-0.538372
33	6	-0.344516	3.336286	1.247615
34	6	-6.999222	1.389309	-1.720518
35	6	-6.300492	0.404769	-0.771164
36	6	5.010158	-3.276432	-0.887145
37	6	-5.030833	-1.346877	0.889683
38	6	2.519407	3.427131	-1.063125
39	6	-7.042538	-0.596920	-0.153132
40	6	3.221500	2.389856	-1.894850
41	6	2.235769	5.664806	-0.283153

42	6	0.815637	3.880181	0.416579
43	6	-6.392270	-1.490457	0.677673
44	6	1.161837	5.223953	0.470386
45	6	2.933657	4.752046	-1.058235
46	1	-8.105431	-0.656419	-0.341899
47	1	-6.937015	-2.292751	1.162755
48	1	-4.512118	-2.034993	1.541860
49	1	-2.745860	0.709518	1.213411
50	1	-2.600890	0.516299	-0.508075
51	1	-2.174299	-1.734008	-1.328282
52	1	-3.056469	-2.740627	-0.192818
53	1	-1.301620	0.375838	2.832508
54	1	-1.010615	-1.020060	3.895554
55	1	-2.272937	-4.873135	-0.063463
56	1	-0.372356	-6.491817	-0.145378
57	1	1.914266	-5.626037	-0.468201
58	1	3.704487	-4.777513	-0.109497
59	1	5.906870	-3.866147	-0.740502
60	1	6.030519	-1.568675	-1.714900
61	1	0.590201	5.888882	1.103011
62	1	2.536863	6.705683	-0.259116
63	1	3.792163	5.055694	-1.644640
64	1	4.169661	2.803516	-2.268872
65	1	2.605283	2.169423	-2.772394
66	1	3.295243	0.147706	-2.983405
67	1	4.956123	0.372774	-2.424802
68	1	1.195127	-0.602852	4.273110
69	1	1.044971	0.932554	3.386058
70	1	3.378058	0.038656	3.534639
71	1	2.875177	-1.381185	2.588693
72	1	4.856183	0.670268	1.902189
73	1	4.418890	-0.651432	0.815851
74	1	5.377190	1.349530	-0.354114
75	1	4.119031	2.347800	0.368717
76	1	-2.817441	-1.626462	2.512983
77	1	-1.414325	-2.559696	2.019545

Zero-point correction= 0.613192 Hartree; Thermal correction to Energy= 0.651869; Thermal correction to Enthalpy= 0.652813; Thermal correction to Gibbs Free Energy= 0.539697; Sum of electronic and zero-point Energies= -2042.192168; Sum of electronic and thermal Energies= -2042.153490; Sum of electronic and thermal Enthalpies= -2042.152546; Sum of electronic and thermal Free Energies= -2042.265663.

(λδλ)-[Ba(**bpycropa**)], dissociated arm rotation, 0 imaginary frequencies

Center Number	Atomic Number	Coordinates (Angstroms) X	Y	Z
1	56	-0.801493	-0.177015	-0.418304
2	8	-0.043217	0.098531	2.340367
3	8	-2.786346	-0.198022	1.674802
4	7	0.451686	2.521515	-0.240810
5	8	-0.058982	-2.560090	0.546635
6	8	6.634186	0.668627	-2.572313
7	7	-2.244626	2.430630	-0.800063
8	7	2.093963	0.219270	0.344760
9	7	-3.776328	-0.130221	-1.062367
10	7	4.969775	-0.612498	-0.793068
11	6	-0.277079	3.630445	-0.083931
12	6	1.781256	2.605317	-0.198651
13	7	-2.386446	-2.585700	-0.802470
14	6	-1.754551	3.511511	-0.186677
15	6	-3.557849	2.342235	-1.013416
16	6	2.441413	3.809824	0.011776
17	6	-2.330613	0.166999	2.962541
18	6	-0.996291	-0.467296	3.215821
19	6	0.314930	4.875607	0.115020
20	6	1.693285	4.961950	0.166434
21	6	2.236693	0.453506	1.778268
22	6	1.273742	-0.352897	2.608960
23	6	-4.445926	3.328948	-0.608320
24	6	4.176508	-1.178377	0.117626
25	6	-4.604000	-0.296918	0.133380
26	6	2.577734	1.350484	-0.432225
27	8	-0.185059	-4.762270	0.909868
28	8	8.416324	-0.229706	-1.558656
29	6	-4.040632	1.127470	-1.753911
30	6	-4.058209	0.348078	1.383554
31	6	2.691218	-1.049665	-0.097137
32	6	-2.589660	4.517288	0.297919
33	6	-0.609037	-3.689774	0.457364
34	6	7.190427	-0.016077	-1.692423
35	6	6.290833	-0.677891	-0.636322
36	6	-3.950809	4.422701	0.079671
37	6	4.680293	-1.844471	1.230031
38	6	-3.560688	-2.560793	-1.429086
39	6	6.870749	-1.325078	0.452082
40	6	-3.998599	-1.237006	-1.990953
41	6	-3.876960	-4.896433	-1.051298

42	6	-1.943157	-3.729196	-0.283707
43	6	6.053651	-1.915999	1.398442
44	6	-2.660162	-4.913536	-0.392340
45	6	-4.341824	-3.699526	-1.572832
46	1	7.949067	-1.354255	0.525084
47	1	6.477587	-2.428821	2.254611
48	1	4.004795	-2.302911	1.942894
49	1	2.163586	-1.858074	0.410416
50	1	2.487256	-1.147406	-1.166897
51	1	2.501161	1.082983	-1.491421
52	1	3.637279	1.565624	-0.247597
53	1	1.340118	-1.425876	2.396237
54	1	1.503976	-0.196816	3.669976
55	1	3.523210	3.833872	0.048940
56	1	2.176429	5.919253	0.320369
57	1	-0.286058	5.769219	0.203933
58	1	-2.186877	5.360043	0.841666
59	1	-4.617836	5.194648	0.443781
60	1	-5.504517	3.222199	-0.808164
61	1	-2.254282	-5.814956	0.045723
62	1	-4.465528	-5.801350	-1.148070
63	1	-5.298643	-3.642552	-2.077185
64	1	-5.052470	-1.302963	-2.299385
65	1	-3.416682	-1.033632	-2.895685
66	1	-3.514299	1.093655	-2.711803
67	1	-5.112472	1.240336	-1.974116
68	1	-0.707642	-0.279240	4.257348
69	1	-1.051797	-1.552384	3.061454
70	1	-3.042377	-0.178577	3.722159
71	1	-2.250118	1.259421	3.037881
72	1	-4.751248	0.128090	2.205443
73	1	-3.983873	1.437143	1.300660
74	1	-5.624171	0.078692	-0.037362
75	1	-4.688772	-1.366024	0.335065
76	1	3.260632	0.257999	2.125933
77	1	2.036636	1.509409	1.971651

Zero-point correction= 0.613623 Hartree; Thermal correction to Energy= 0.652952; Thermal correction to Enthalpy= 0.653896; Thermal correction to Gibbs Free Energy= 0.538032; Sum of electronic and zero-point Energies= -2042.197765; Sum of electronic and thermal Energies= -2042.158436; Sum of electronic and thermal Enthalpies= -2042.157492; Sum of electronic and thermal Free Energies= -2042.273356.

(λδλ)-[Ba(**bpycropa**)], dissociated arm coordination, 1 imaginary frequency

Center Number	Atomic Number	Coordinates (Angstroms) X	Y	Z
1	56	-0.454584	-0.207688	-0.082181
2	8	-0.514811	0.737834	2.661769
3	8	-2.929607	-0.056994	1.390848
4	7	0.451438	2.614042	-0.143642
5	8	0.034750	-2.215287	1.612517
6	8	4.054028	-1.177931	-3.383231
7	7	-1.979553	2.087281	-1.306058
8	7	2.056904	0.692190	1.314719
9	7	-3.140989	-0.659409	-1.448609
10	7	4.103442	-0.869244	-0.641804
11	6	-0.376925	3.634228	-0.382343
12	6	1.711324	2.865464	0.209015
13	7	-1.693236	-2.814089	-0.354348
14	6	-1.758169	3.314011	-0.825519
15	6	-3.172844	1.796977	-1.824249
16	6	2.198144	4.159648	0.344572
17	6	-2.885110	0.625551	2.628220
18	6	-1.636135	0.244317	3.363584
19	6	0.042706	4.958658	-0.278820
20	6	1.347884	5.220486	0.092352
21	6	1.805310	1.235231	2.644463
22	6	0.696759	0.511334	3.361656
23	6	-4.209170	2.719617	-1.869832
24	6	4.136856	-0.585465	0.660466
25	6	-4.233943	-0.757010	-0.481195
26	6	2.627010	1.692253	0.423678
27	8	0.073085	-4.342048	2.301707
28	8	6.281260	-1.404100	-3.386043
29	6	-3.341162	0.422177	-2.406722
30	6	-4.134556	0.186254	0.692037
31	6	2.813891	-0.584806	1.372444
32	6	-2.766273	4.275901	-0.786190
33	6	-0.307676	-3.426193	1.559161
34	6	5.177021	-1.183470	-2.841553
35	6	5.238144	-0.872987	-1.338219
36	6	-4.004843	3.971921	-1.318383
37	6	5.319740	-0.290178	1.327584
38	6	-2.603409	-3.064734	-1.292127
39	6	6.465015	-0.594061	-0.740870
40	6	-2.997143	-1.916655	-2.179149
41	6	-2.743645	-5.352263	-0.625535

42	6	-1.309180	-3.790211	0.466298
43	6	6.504496	-0.293985	0.608802
44	6	-1.807010	-5.081642	0.357120
45	6	-3.159504	-4.326127	-1.458655
46	1	7.358997	-0.621838	-1.348446
47	1	7.445246	-0.068537	1.098429
48	1	5.308064	-0.064446	2.387210
49	1	2.977530	-0.832241	2.423322
50	1	2.207329	-1.393472	0.959236
51	1	2.811066	1.221959	-0.546045
52	1	3.596955	2.068666	0.778086
53	1	0.886307	-0.566833	3.421403
54	1	0.610569	0.905915	4.381697
55	1	3.226186	4.322939	0.642172
56	1	1.697534	6.242140	0.179070
57	1	-0.627538	5.774869	-0.507384
58	1	-2.591631	5.246714	-0.344714
59	1	-4.801849	4.705392	-1.302351
60	1	-5.161378	2.445509	-2.305937
61	1	-1.459044	-5.838984	1.046005
62	1	-3.158706	-6.347605	-0.733738
63	1	-3.909010	-4.493403	-2.222490
64	1	-3.917337	-2.177965	-2.722816
65	1	-2.215884	-1.775422	-2.933312
66	1	-2.592469	0.306293	-3.195327
67	1	-4.329675	0.349777	-2.883780
68	1	-1.672216	0.681444	4.369357
69	1	-1.567422	-0.846501	3.460153
70	1	-3.754849	0.349998	3.237186
71	1	-2.912221	1.710505	2.460828
72	1	-4.989168	-0.008248	1.352396
73	1	-4.185015	1.238309	0.394228
74	1	-5.207787	-0.602668	-0.970685
75	1	-4.235926	-1.771583	-0.079844
76	1	2.709789	1.215959	3.270084
77	1	1.508958	2.281272	2.543240

Zero-point correction= 0.613448 Hartree; Thermal correction to Energy= 0.651919; Thermal correction to Enthalpy= 0.652863; Thermal correction to Gibbs Free Energy= 0.540816; Sum of electronic and zero-point Energies= -2042.191180; Sum of electronic and thermal Energies= -2042.152709; Sum of electronic and thermal Enthalpies= -2042.151765; Sum of electronic and thermal Free Energies= -2042.263812.

Λ(λδλ)-[Ba(**bpycropa**)], dissociated arm coordination, 0 imaginary frequencies

Center Number	Atomic Number	Coordinates (Angstroms) X	Y	Z
1	56	-0.073427	-0.004342	0.052666
2	8	0.998730	-0.275771	2.759040
3	8	0.246906	2.317822	1.819455
4	7	2.740616	-1.044516	-0.281719
5	8	-2.057122	-0.751354	1.759602
6	8	-0.928186	-0.228292	-2.507796
7	7	2.217348	1.499713	-1.184645
8	7	0.806531	-2.694726	1.083202
9	7	-0.484070	2.813668	-0.955553
10	7	-1.078681	-2.459527	-1.002446
11	6	3.768784	-0.223473	-0.513340
12	6	2.978382	-2.332574	-0.037904
13	7	-2.647308	1.327825	0.148176
14	6	3.465339	1.200484	-0.815643
15	6	1.926419	2.746065	-1.558261
16	6	4.264733	-2.857541	-0.009282
17	6	0.989378	2.086745	2.997744
18	6	0.573979	0.776129	3.598663
19	6	5.084878	-0.680987	-0.520340
20	6	5.332079	-2.015741	-0.261035
21	6	1.354772	-2.599451	2.434720
22	6	0.689192	-1.552344	3.289143
23	6	2.877142	3.757871	-1.572295
24	6	-1.096963	-3.559161	-0.254017
25	6	-0.410746	3.827406	0.095342
26	6	1.795638	-3.239074	0.165758
27	8	-4.134473	-0.760453	2.587704
28	8	-2.246478	-1.136263	-4.070412
29	6	0.514383	3.006305	-2.001884
30	6	0.582963	3.539934	1.196141
31	6	-0.405601	-3.503183	1.080642
32	6	4.460300	2.175486	-0.752840
33	6	-3.233231	-0.317857	1.857921
34	6	-1.623629	-1.156154	-2.998057
35	6	-1.703319	-2.445413	-2.179257
36	6	4.159370	3.467250	-1.141036
37	6	-1.756230	-4.711250	-0.665924
38	6	-2.896061	2.384614	-0.622275
39	6	-2.396139	-3.550508	-2.653437
40	6	-1.808950	2.802314	-1.571637
41	6	-5.098013	2.590223	0.278415

42	6	-3.583945	0.886957	0.985273
43	6	-2.421173	-4.699977	-1.880845
44	6	-4.831782	1.490550	1.075704
45	6	-4.114084	3.051173	-0.581697
46	1	-2.891165	-3.490678	-3.612874
47	1	-2.945476	-5.584097	-2.224935
48	1	-1.742304	-5.598206	-0.044153
49	1	-0.198714	-4.530455	1.419733
50	1	-1.104155	-3.054914	1.793931
51	1	1.314758	-3.384965	-0.805541
52	1	2.153577	-4.226408	0.495787
53	1	-0.398797	-1.677725	3.324209
54	1	1.081323	-1.631629	4.311117
55	1	4.417076	-3.908076	0.203924
56	1	6.347244	-2.393940	-0.258905
57	1	5.905396	-0.014796	-0.744917
58	1	5.454204	1.935982	-0.402420
59	1	4.918393	4.239368	-1.104840
60	1	2.603846	4.755773	-1.891066
61	1	-5.559921	1.091594	1.768317
62	1	-6.058132	3.090659	0.330653
63	1	-4.281039	3.917033	-1.210852
64	1	-2.061450	3.780942	-2.007324
65	1	-1.777359	2.071590	-2.385898
66	1	0.282465	2.296771	-2.800585
67	1	0.451407	4.019992	-2.426194
68	1	1.036690	0.677076	4.589044
69	1	-0.516355	0.743931	3.721566
70	1	0.802278	2.889042	3.722777
71	1	2.064350	2.073205	2.772018
72	1	0.524794	4.358648	1.925548
73	1	1.614993	3.499979	0.831823
74	1	-0.187938	4.819774	-0.326264
75	1	-1.395290	3.895057	0.562050
76	1	1.301644	-3.570473	2.950997
77	1	2.412330	-2.338404	2.357361

Zero-point correction= 0.613730 Hartree; Thermal correction to Energy= 0.652600; Thermal correction to Enthalpy= 0.653544; Thermal correction to Gibbs Free Energy= 0.541806; Sum of electronic and zero-point Energies= -2042.223096; Sum of electronic and thermal Energies= -2042.184226; Sum of electronic and thermal Enthalpies= -2042.183282; Sum of electronic and thermal Free Energies= -2042.295020.
